# Skin cancer: understanding the journey of transformation from conventional to advanced treatment approaches

**DOI:** 10.1186/s12943-023-01854-3

**Published:** 2023-10-06

**Authors:** Nazeer Hasan, Arif Nadaf, Mohammad Imran, Umme Jiba, Afsana Sheikh, Waleed H. Almalki, Salem Salman Almujri, Yousuf Hussain Mohammed, Prashant Kesharwani, Farhan Jalees Ahmad

**Affiliations:** 1 Department of Pharmaceutics, School of Pharmaceutical Education and Research, Jamia Hamdard, New Delhi, 110062 India; 2 Frazer Institute, Faculty of Medicine, University of Queensland, Brisbane, 4102 Australia; 3 Department of Pharmacology and Toxicology, Faculty of Pharmacy, Umm Al-Qura University, 24381 Makkah, Saudi Arabia; 4 Department of Pharmacology, College of Pharmacy, King Khalid University, 61421 Asir-Abha, Saudi Arabia; 5 Center for Global Health Research, Saveetha Medical College and Hospitals, Saveetha Institute of Medical and Technical Sciences, Saveetha University, Kuthambakkam, India

**Keywords:** Skin cancer, Melanoma, Non-melanoma, Etiology, Phytocompounds, Toxicity, Targeted therapy, Combination therapy, Nanotechnology

## Abstract

Skin cancer is a global threat to the healthcare system and is estimated to incline tremendously in the next 20 years, if not diagnosed at an early stage. Even though it is curable at an early stage, novel drug identification, clinical success, and drug resistance is another major challenge. To bridge the gap and bring effective treatment, it is important to understand the etiology of skin carcinoma, the mechanism of cell proliferation, factors affecting cell growth, and the mechanism of drug resistance. The current article focusses on understanding the structural diversity of skin cancers, treatments available till date including phytocompounds, chemotherapy, radiotherapy, photothermal therapy, surgery, combination therapy, molecular targets associated with cancer growth and metastasis, and special emphasis on nanotechnology-based approaches for downregulating the deleterious disease. A detailed analysis with respect to types of nanoparticles and their scope in overcoming multidrug resistance as well as associated clinical trials has been discussed.

**Graphical Abstract:**

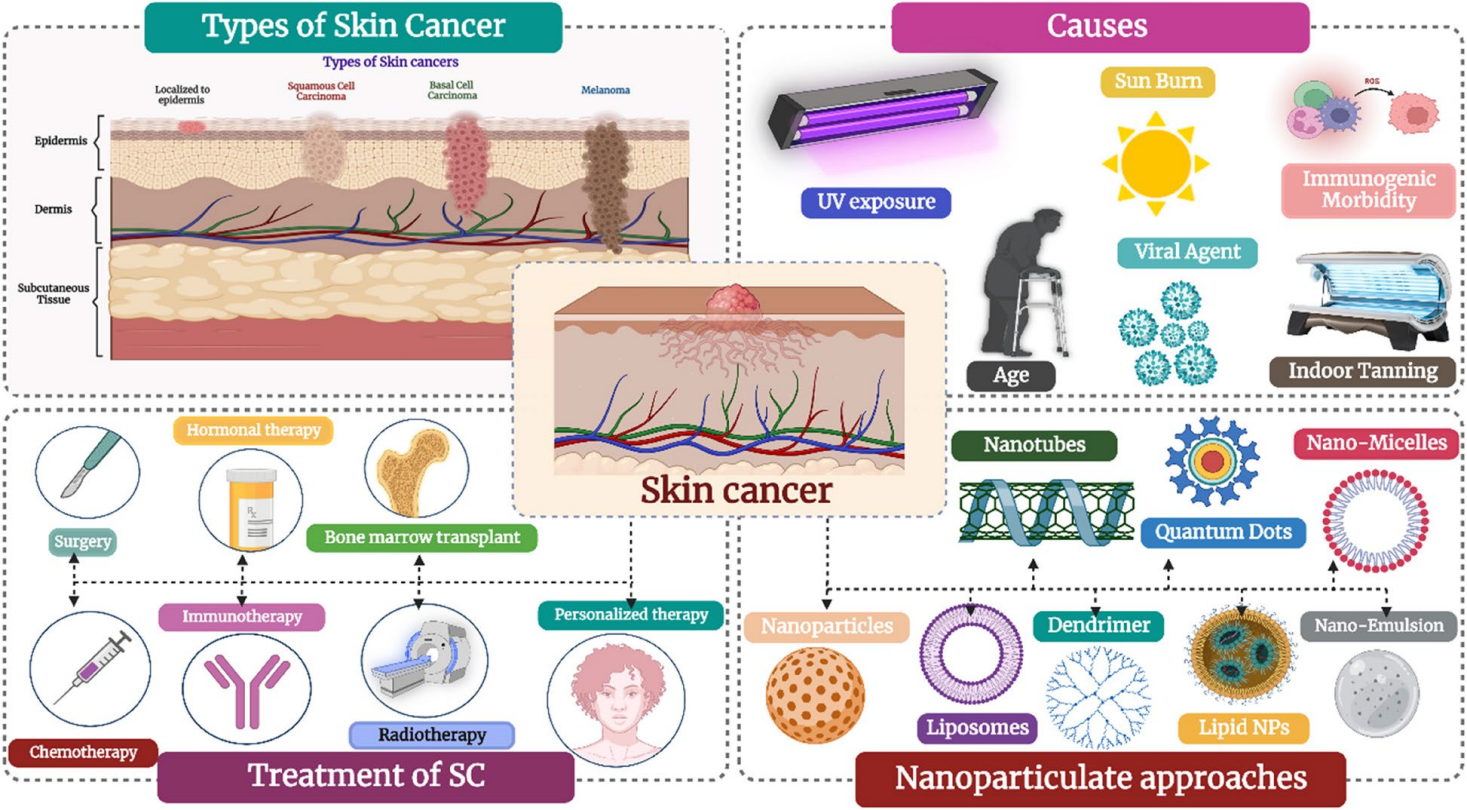

## Introduction

Skin cancer (SC), is one of the most devastating cancers of the present decade, and the fifth commonest form of cancer [1–3]. It is further predicted to surpass heart disease as the main cause of mortality and the biggest obstacle to extending life expectancy in the next decades. According to the annual status report from the International Agency for Research on Cancer, there were approximately 9.6 million cancer-related fatalities and 18.1 million new instances of cancer globally in 2018. As per the statistics presented by the American Cancer Society, the new cases of melanoma among all cancers are estimated to be 6% in males and 4% in females in the year 2023. Furthermore, it is expected that the number of new cases would continue to rise over the next 20 years [4–8].

The etiology primarily lies beside the abnormal skin cell proliferation facilitated by the unrepaired DNA of skin cells which owes to DNA mutations or genetic defects. The skin's tissue is divided into two layers: the top epidermis, which is made up of epithelial cells and pigmented melanocytes, and the bottom dermis, which comprises a layer of connective tissue that holds blood vessels, hair follicles, and sweat glands [9–13]. Cancer cells that emerge from mutations in skin melanocytes are termed malignant melanoma. Non-melanoma skin cancer (NMSC), which develops from the epidermis, is the kind of skin cancer that is most often seen worldwide. Based on the kind of cells involved, it is further classified as Squamous cell carcinoma (SCC) and basal cell carcinoma (BCC). In around 25% of the cases that were recorded, a mole transformed into a case of Multiple Myeloma (MM) grew and metastasize. A significant risk of recurrence is associated with this MM kind of skin cancer. NMSC rarely migrates into the deeper tissues of the epidermis if unnoticed. It can only be easily eliminated when it is found at an early stage. Thus, SC must get significant funding for technology and treatment since it negatively affects the social and psychological well-being of an individual [14–17].

Till date, the treatment includes surgery, chemotherapy, and radiation therapy. However, such treatments do not cure ailments, are painful for the patients, and have several negative consequences. They often affect normal, healthy cells as well without causing sufficient toxicity to the cancer cells [3, 18–21]. Since there are tumor-ablative and function-reserving oncologic treatments, the use of phototherapies, such as photodynamic therapy (PDT) and photothermal therapy (PTT), in clinical cancer therapy offers great potential. Safe phototherapeutic compounds may be triggered by light irradiation throughout phototherapies, selectively eliminating cancer cells without producing negative side effects [22–26].

Nanotechnology is one of the cutting-edge technologies used to develop a possible controlled-release drug delivery carrier with a great potential for delivering drugs efficiently. Nanoparticles provide target- and site-specific delivery because of their ability to pass through physiological barriers. The nanoparticles also enhance the effectiveness of drugs at low dosages and facilitate the management of chronic conditions by reducing adverse effects [27–31].

The novel tactics appeal to a wide range of industries, from materials engineering to biomedicine, since it allows for the design of functioning systems at the nanoscale. Nanotechnology in the field of medical science provides a platform for highly specialized medical treatments for the prevention, diagnosis, and treatment of illnesses, including cancer. Advancements in the field of nanotechnology in medicine over the last two decades have made it possible to incorporate a variety of therapeutic, sensing, and targeting agents into nanoparticles [32–37]. It is a revolutionary approach to designing, preparing, and using systems, gadgets, and structures by sculpting and modifying their dimensions at the nanoscale.

According to the National Nanotechnology Initiative (NNI), a government-sponsored US research and development initiative, the production of carriers, devices, or systems with sizes ranging from 1 to 100 nm, with the potential to reach 1000 nm, is referred to as nanotechnology [8, 35].

Traditional medicine has been practiced by the world since ancient times utilizing natural bioactive components. Due to their wide range of therapeutic benefits in reversing the progression of a disease, natural dietary phytochemicals have generated a lot of attention in this area. These bioactive phytochemicals found in a wide range of foods and beverages, are less toxic and effective as medicines. They can be incorporated into the host system's natural defense against parasites, viruses, and other external stimuli [38].

In certain circumstances, phytochemicals may especially help with the treatment of skin cancer. First, both the patient and the practitioner have easy access to precancerous and cancerous skin lesions. This assists in the design of topical treatments that can be administered just to the suspected malignant region of change while causing little harm to healthy skin. It may be necessary to provide a phytochemical orally to cure different internal organ tumors, which would have an impact across the body. Second, both doctors and patients can quickly assess if a skin lesion therapy was successful. Skin biopsies are very minimally invasive, although most malignancies need invasive pathological proof. Future investigations on the efficiency of phytochemicals in treating skin cancer may therefore be made simpler. The majority of local adverse effects may be readily identified using topical treatments, which may help patients experience less pain and lower their risk of developing more severe or long-lasting side effects.

Several phytochemicals, including epigallocatechin-3-gallate, capsaicin, silymarin, indole-3-carbinol, proanthocyanins, curcumin, resveratrol, luteolin, apigenin, and genistein may be found in fresh fruits, vegetables, roots, and herbs. They are thought to support cancer chemoprevention and treatment in a variety of ways [39–41].

The ongoing research prompted advanced knowledge in the therapeutic regimen, however, the condition is still fatal. In a way, current therapy limitations indicate the need for innovative therapeutics. Because conventional therapies have several limitations, effective alternatives must be found. Among the various therapeutic techniques, nanotechnology offers extraordinary potential on the molecular level through targeted interactions with cancer cells and suppression of their activity. Numerous compounds have already entered medical practice and have become the norm. The most exciting is combination therapy, which combines herbal treatment with the conventional drug into a unique formulation [42].

In this study, we provide an impression of the tremendous development of natural chemicals with synthetic drugs with excellent effectiveness and safety in skin cancer combination therapy accomplished by flexible nanotechnologies, which focus on solid-resistant skin tumor mechanisms. The section below discusses types of skin cancer, factors, and etiology associated with its progression, current therapeutic regimen, and the scope of nanotechnology in overcoming the treatment barrier.

## Skin cancer

Skin cancer is the commonest form of cancer and approximately one out of five people suffer from skin cancer anywhere in their lifetime. Low mortality cases were observed in the case of SC due to early detection and proper treatment. Cancer is the uncontrolled and unorderly growth of normal cells, and the capacity of losing the controlled growth of normal cells is termed contact inhibition of proliferation [43]. There are two main types of skin cancer which are specified as non-melanoma skin cancer and melanoma skin cancer and the former is further categorized into basal cell carcinoma (BCC) and squamous cell carcinoma (SCC). Non-melanoma skin cancer (NMSC) or keratinocyte skin cancer (KSC) is the most common form of skin cancer [44].

### Non-melanoma skin cancer

The most common cancer diagnosed in Australia, New Zealand, and North America is NMSC. Globally, according to Globocan's estimate, there were 1,042,056 new cases of NMSC in 2018, and 65,155 deaths, or almost 6% of deaths, were related to NMSC (mostly SCC). These numbers mirror those from Spain, according to reports [45]. NMSC skin cancer is a kind of skin cancer in which cells other than melanoma cells are affected by the cancer. The rate of occurrence of NMSC is increasing by 10% per year. The most common reason for NMSC occurrence is Ultraviolet (UV) rays have a high risk in persons with lighter shade. NMSC can also be developed due to Genetic mutation such as a mutation in certain gene families named CYP450, GST(Glutathione S-transferase), p53 [46]. There are several distinctive forms of NMSC which are caused by viruses such as verrucous carcinoma, Bowenoid papulosis, epidermodysplasia verruciformis, squamous cell carcinoma, Kaposi sarcoma, and the most common as well as high occurrence rate named Merkel cell carcinoma [47].

#### Basal cell carcinoma (BCC)

BCC is the most common type of malignancy of skin found in humans. The biggest risk is being exposed to sunlight. Due to its low fatality rate, BCC is not typically included in cancer registries; nonetheless, after analyzing data from insurance registries and official statistics in the United States (US), BCC incidence has been projected to reach 4.3 million cases annually [48]. The mortality rate is low but the morbidity rate is high due to local destruction of tissues [49]. Of all skin cancer patients, about 50% are suffering from BCC. BCCs are significantly more prevalent among Caucasians [50]. Generally, the cells in the epidermis concluded as keratinocytes in which basal cells are found in the bottom layer of epidermal cells are also a type of keratinocytes. It is the least destructive form of cancer that appears as light pinkish- or flesh-colored pearls same as bump or pinkish patches of skin. The major threat of having BCC is in the superficial layers including the face, hands, neck, legs, abdomen, or areas that are in direct exposure to UV rays emerging from the sun. It can expand throughout the body surface or can escalate to nerves or bones. The main causative agent for BCC includes (i) exposure to sun for a long time, (ii) immunity not able to cope with the cancer-inducing agents, (iii) beta human papilloma virus (HPV) and (iv) Human immunodeficiency virus (HIV) [51]. Cell growth is regulated by the patched/hedgehog intracellular signaling system, and continuous activation of this pathway results in the formation of BCC. The mutations that lead to abnormal hedgehog pathway activation and tumor formation are inactivating mutations of PTCH1 or activating mutations of SMOm. In a tiny subset of BCCs, a loss-of-function mutation in SUFU, an antagonistic modulator of the hedgehog pathway, also has been discovered. UV-specific abnormalities in the p53 tumor suppressor gene, which are present in half of BCCs, are another prevalent mutation [52].

**Fig. 1:**
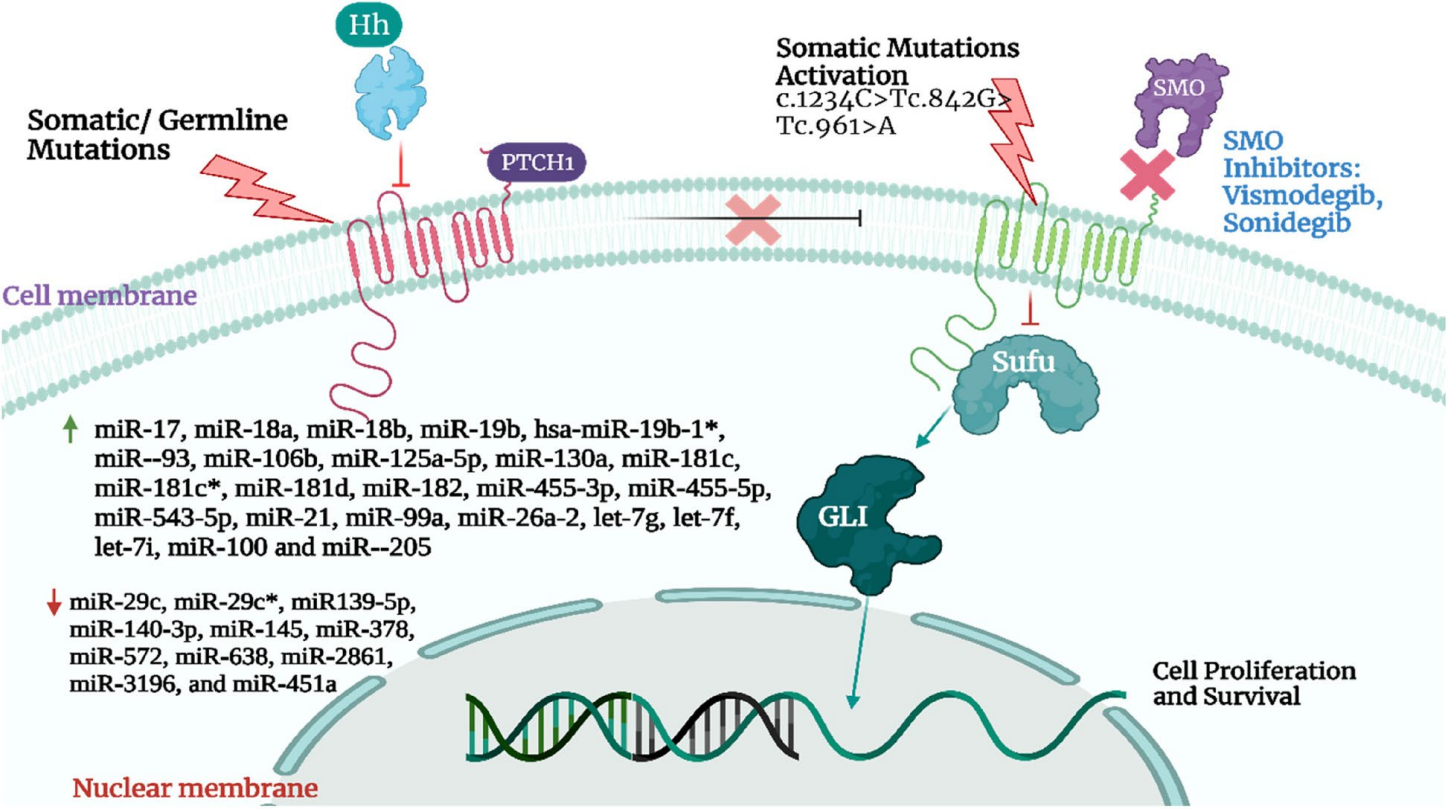
Illustration of different molecular pathways involved in Basal cell carcinoma. Adapted with permission from [50]

Generally, it can be treated by radiation therapies or topical treatment including 5-fluorouracil or any combination [53]. Treatments are directed to local control only as it is very low metastatic in nature. The length of follow-up and the proportion of high-risk and recurrent cancers should both be taken into account when comparing the cure rates for treatments based on various research. For instance, BCCs' sluggish rate of growth makes it common to detect recurrences five years after the first diagnosis. A randomized controlled study with surgical excision found that the recurrence rate was 3% at 2.5 years and 12% at 10 years, respectively, and that 56% of recurrences happened more than 5 years after treatment [54, 55]. Figure 1 represents different molecular pathways involved in Basal cell carcinoma. The physiological parameters of various kinds of skin cancer are mentioned in the section below.

#### Squamous cell carcinoma

**Fig. 2:**
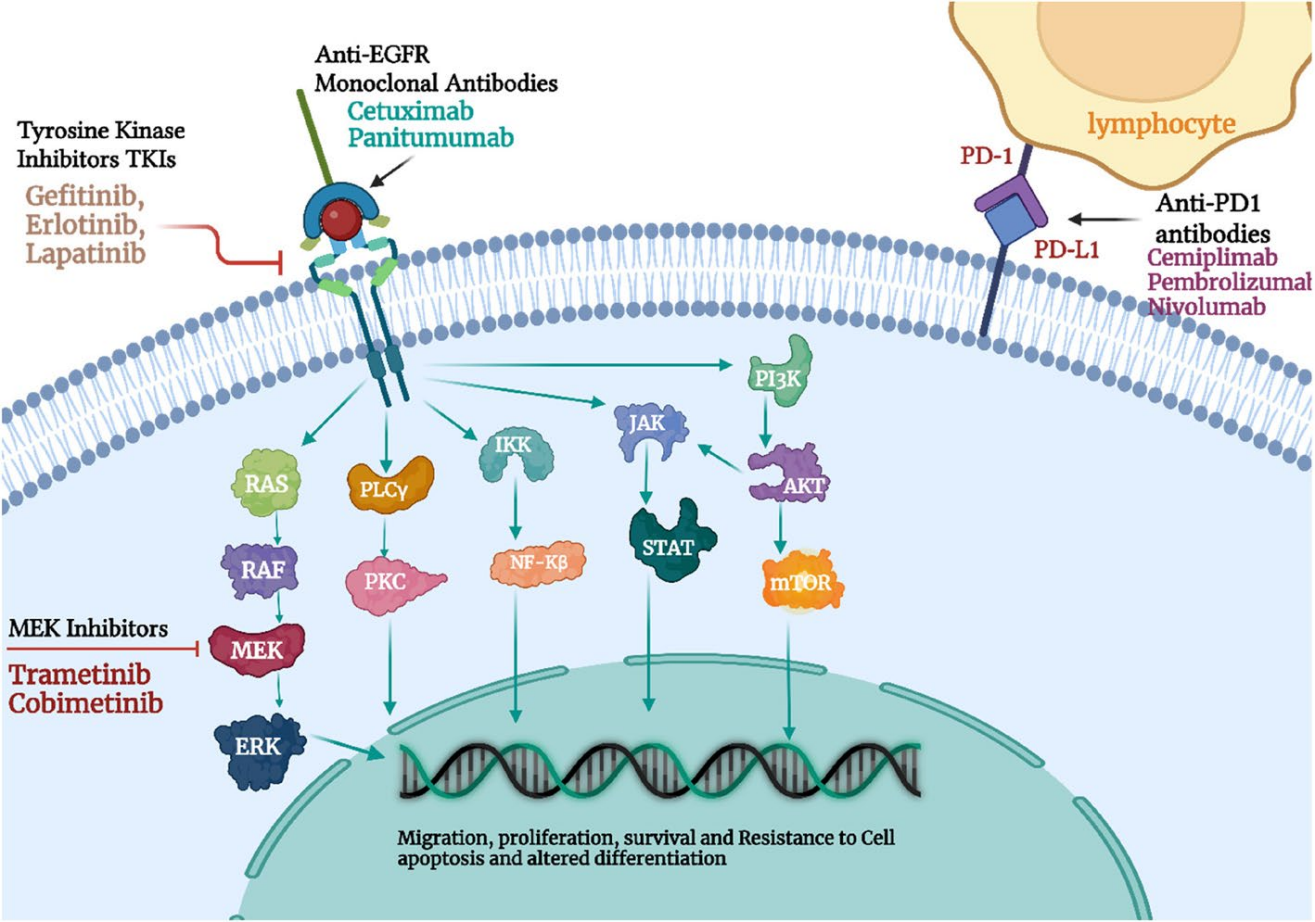
Representation of therapeutic landscapes involved in Cutaneous cell carcinoma. Adapted with permission from [56]

With an estimated 1 million cases each year in the US, cutaneous squamous cell carcinoma (CSCC) is the second most common malignancy in people. This estimate represents an increase in the number. Throughout the last three decades, the number of CSCCs has climbed from 50 to 300%, and by 2030, their frequency in European countries will be twice as high as it is now. It is the second most frequent type of skin cancer which is counted as 2.5 lakh people get effected with SCC in the US every year. In comparison to BCC, SCC has more occurrence risk as well as significant mortality rates (0.3–3.7%). According to estimates, the Caucasian population’s lifetime chance of having a CSCC ranges from 7 to 11% (9% to 14% for men and 4% to 9% for women) [56]. Although it typically displays benign clinical behavior, it has the potential to spread locally and metastatically. For CSCC, ten-year survival following surgery is above 90%, although it rapidly decreases when metastases appear. Lymph node metastases occur about 4% of the time, and mortality is close to 2% [56]. Squamous cell carcinoma is the secondary kind of skin cancer with the occurrence of 99% in all NMSCs patients and occurs in the keratinocyte cells of the outer/upper layer of the epidermis and seems like scaly patches of red firm bumps [57]. It is due to overexposure to UV rays and normally occurs in light-shady people. There are several factors that are responsible for SCC occurrence including light complexion, aging, burn scars, chronic skin ulcers, immune suppression, chemical carcinogens and majorly caused by UV rays [46]. Several molecular pathways have been linked to the development of CSCC. Early occurrences in CSCC, such as ultraviolet-induced P53 mutations, cause significant genomic instability. As we will see later, CSCC has the highest mutational burden of any solid tumor, which may have therapeutic implications. Thereafter, oncogenes like RAS and other suppressor genes like CDKN2A and NOTCH undergo additional genetic alterations. Epidermal growth factor receptor (EGFR) upregulation is eventually caused by the activation of several signaling pathways, including the Nuclear factor kappa B (NF-kB), Mitogen-activated protein kinase (MAPK), and phosphoinositide 3 kinase (PI3K)/Akt/mammalian (or mechanistic) target of rapamycin mTOR pathways. Alterations to the epigenome may also occur [56, 58–63]. The pathways are represented in Fig. 2. The mainstay of treating CSCC is surgery, though radiation is occasionally used as well. But some individuals with locally progressed and metastatic CSCC might gain from systemic therapies [64].

### Melanoma skin cancer

Melanoma are aggressive malignant tumors that arise from melanocytes. Melanocytes are located in the epidermis' basal layer. A buildup of genetic alterations that activate oncogenes, deactivate tumor suppressor genes and hinder DNA repair when organisms are exposed to ultraviolet radiation. This mechanism may result in unchecked melanocyte growth and ultimately malignancy [65]. An estimated 70,000 people in the US were diagnosed with malignant melanoma in 2007 in which most of them were cured but even so, there is approximately the count of 1000 people that died each year from melanoma skin cancer. Immensely 75% of mortality cases were of malignant melanoma alone in all skin cancer-suffering patients [66, 67]. It is an odd but more deliberate kind of cancer in comparison to NMSC. So, the early spotting of the disease is much more important to cure the disease properly with less damage [68]. For detecting melanoma carcinoma physically, we can follow the ABCD rule which includes firstly, *Asymmetry* – both sides of the wound don’t look alike, Secondly, *Border-irregularity* – edges of the skin cells suffering from melanoma are not well ordered or systematic or even, Thirdly, *Color* – this carcinoma is an amalgam of colors, including brown, black, tan, blue or red, and at last, *Diameter* – the diameter of the cancerous area is generally greater than 6 mm (approx. a size of pencil eraser), but variation in size may be possible [66].

**Fig. 3:**
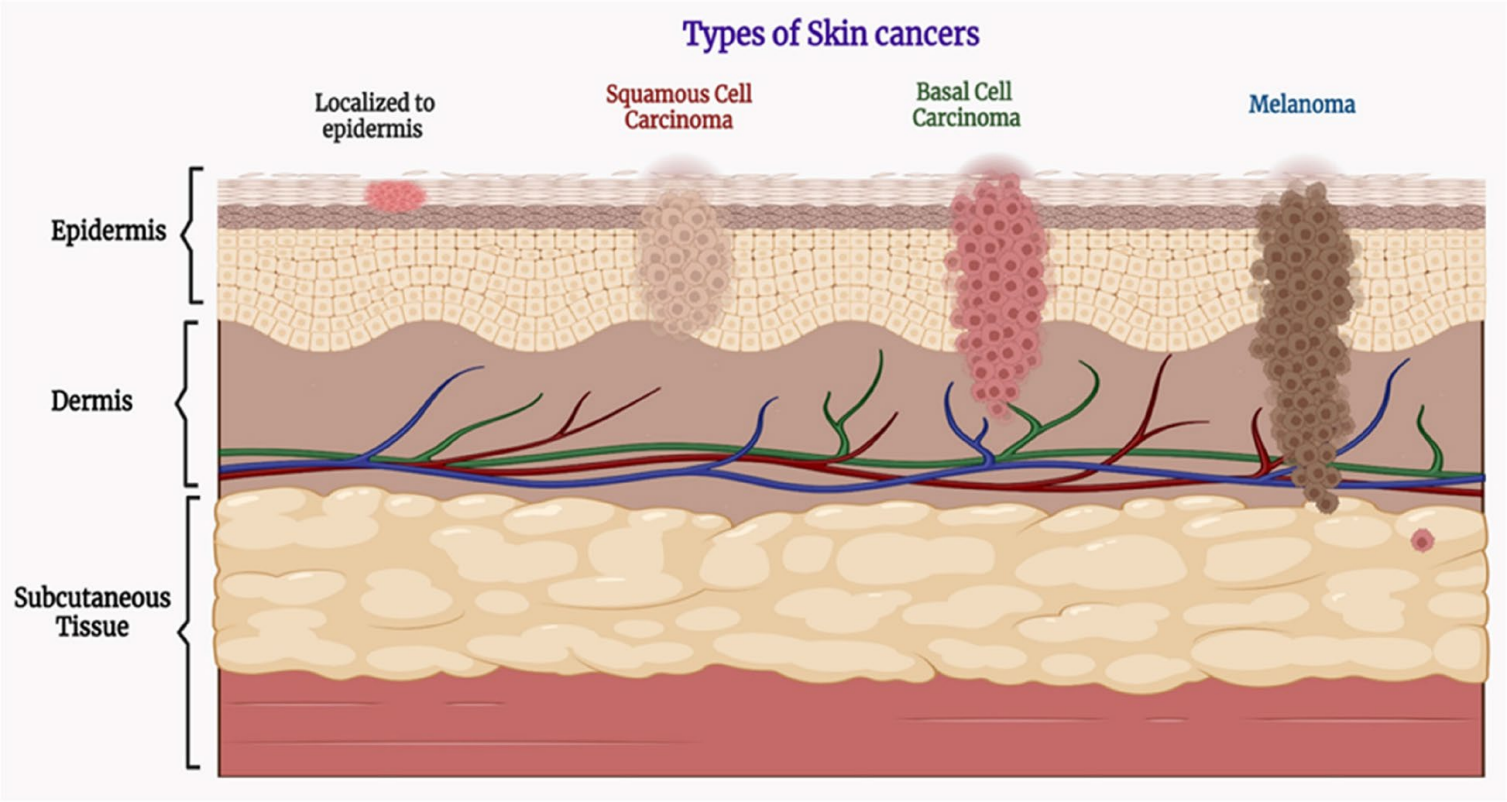
Illustrates different types of Skin cancers

Figure 3 represents different types of skin cancer.

The prognosis for individuals with cutaneous melanoma depends on the depth and location of the initial tumor, as well as whether or not they have localized or distant metastatic disease. For their distinctive histologic characteristics, the four main subtypes of invasive cutaneous melanoma are categorized as follows: superficial, nodular, lentigo malign, and acral lentiginous. Superficial melanomas are the commonest of subtypes accounting for about 75% of melanomas. It appears as a mole-like pigmented skin lesion that has changed in size, shape, or color. In women, it is manifested on the legs while in men it typically manifests on the trunk [69, 70]. Nodular melanoma accounts for about 15- 30% of melanoma cases. It is an aggressive, vertically developing melanoma. As it lacks a radial growth phase, it expands in depth more rapidly than in width. Because of this, it could take more time for someone to become suspicious about the lesion. Nodules that are pedunculated or polypoid in shape are the typical presentation of nodular melanoma. Older people's heads and necks are more likely to develop nodular melanomas, which are hard, symmetrical, evenly pigmented papules or nodules that can ulcerate and bleed [71].

The second most prevalent subtype of melanoma is lentigo malign (LM). It often appears as a small, flat, tan, irregular-bordered, asymmetric macule on sun-exposed areas. With time, the macule grows larger and starts to change color. Hutchinson's freckle, also known as lentigo malign, begins as a pigmented macule that grows slowly and may stay in place for many years. These melanomas are substantially more prevalent in adults over the age of 60 and are commonly referred to as “senile freckles". These may progress quickly once they become invasive. They frequently have poor definition and variable pigmentation. The way LM is managed is always changing. If available, the first course of treatment is surgery, followed by radiation, and in patients where surgery is not an option imiquimod cream is used [72, 73].

Acral lentiginous melanomas (ALM) are a rare subtype of melanoma. It makes up about 2 to 3% of all melanoma diagnoses, making it the least prevalent melanoma subtype. It generally occurs on soles, toes, palms, fingers, and nail beds. Unlike other types of melanomas, it is not linked to sun exposure. It is the most prevalent subtype of melanoma diagnosed in people of Asian or African heritage and tends to be more advanced at presentation due to delays in detection. Due to the complexity and functional significance of the hands and feet, surgical treatment is challenging, and repair is frequently required after resection. The prognosis for people with this type varies with disease stage and is typically poorer than with other melanoma subtypes. Patients with advanced ALM are being investigated for response to newer therapeutic modalities, including immunotherapies and targeted drugs, with some encouraging early findings [74, 75].

## Mechanism of skin cancer progression

Skin cancer progression involves a complex interplay of genetic, molecular, and environmental factors that lead to the transformation of normal skin cells into cancerous ones. it can happen because of many reasons including direct exposure to UV radiation on exposed areas of skin, immunogenic skin problems, DNA damage due to various reasons, skin color, sun tanning bed, and many more. Several etiologic agents that cause skin cancer were described below.

### UV rays

Ultraviolet radiation is the predominant root-cause of skin cancer. It is responsible for more than 80% of skin cancer cases [76]. There are three types of UV radiation: UV-A, UV-B, and UV-C. UV-A and UV-B make up the majority of the components of sunlight (90% and 10% respectively). The atmosphere primarily absorbs UV-C rays. As UV-A has a longer wavelength (320–400 nm), it can enter the dermis and produce free radicals there. The epidermis' stratum basale is reached by UV-B, which has a shorter wavelength (290–320 nm) and induces the synthesis of thymine dimers. Both UV-A and UV-B influence the development of cancer, but UV-A is regarded to be more important. UV light damages cells induces apoptosis, and hinders DNA repair processes, all of which result in DNA mutations [77]. UV radiation can destruct the DNA and cause genetic mutations, which further damage the skin cells and cause skin cancer [78]. UV exposure causes DNA damage that results in mutation and lowers the host immune system's capacity to detect and eliminate cancerous cells, which together create a two-fold mechanism that causes carcinogenesis. There are various factors that can guide the quantity of UV radiation reach to the earth's surface and can control its harmful effect counting as ozone depletion control, gratitude, latitude, less use of products having CFC (chlorofluorocarbon), keeping control of climatic change and many more. UV radiation is a remarkable and universal physical cancer-inducing agent in our natural surroundings [79]. UV rays are a major etiological aspect of basal cell carcinoma [80].

### Skin color

Skin cancer is the most prevalent malignancy in the USA, accounting for 35–45% of all neoplasms in Caucasians, 4–5% in Hispanics, 2–4% in Asians, and 1–2% in Blacks [81]. It is experimentally proved that white Caucasian skin is more perilous or unsafe than pigmented skin for skin cancer [79] for both melanoma and non-melanoma. The greater melanin in the epidermis, which screens twofold as much UV light as that in Caucasians' epidermis, is principally responsible for the lower incidence of skin malignancies in darker-skinned races. Darker-skinned populations have large and more melanized melanosomes which absorb and disperse energy higher efficiently than Caucasians' smaller, melanosomes [82]. Skin cancer in non-White people frequently manifests at a later stage, which makes the prognosis poorer than in White individuals. Skin cancer patients of color experience higher morbidity and fatality rates than white patients, which may be attributed to a lack of awareness, more advanced diagnosis, and socioeconomic issues including barriers to care [83]. NMSC has much more incidence rates in comparison to MSC in Caucasian skin cancer patients [84]. In dark-skinned persons, squamous cell carcinoma, a kind of NMSC is most common, whereas in Caucasians basal cell carcinoma, another class of NMSC is most common [80, 85]. All racial groupings generally receive the same treatment [81].

### Immunogenic morbidity

Immunosuppression has a strong probability of causing skin cancer these days specially NMSCs. White solid organ transplant recipients (SOTRs) are 50% more likely to develop skin cancer. The average time from transplantation to diagnosis is three to eight years, and more than 90% of tumors are BCC or SCC. Multiple tumors are frequently discovered in SOTRs, and these tumors typically exhibit greater aggressiveness than those found in the entire population [86]. Melanomas account for a larger percentage of skin malignancies in the pediatric SOTR group than in adult receivers (12% vs. 5% of all skin cancers, respectively) [87]. When an organ transplant takes place in any individual, the chances for the occurrence of NMSCs gradually increase, in HIV patients, or patients having chronic lymphocytic leukemia, etc. the chances of skin cancer are much more higher [88]. Immunosuppressive medicines accelerate cutaneous carcinogenesis. SCC and BCC occurrences are 65-fold to 250-fold and tenfold higher in transplant recipients than in the general population, respectively, and the incidence of skin cancer keeps increasing years later [89]. It is important to note that T-cell immunosuppression is highly elevated in skin cancers. Neoepitopes, tumor-associated antigens, and/or viral oncoproteins are assumed to be the sources of the skin malignancies' antigenicity. Tumor cells interact with immune system elements that are functioning to impede the proliferation and metastasis of melanoma throughout the melanomagenesis process. Although Breslow thickness and lymph node metastasis are still regarded as poor prognostic indicators, melanoma cells' tendency to infiltrate distant organs likewise relies on how they interact with other cells in the tumor microenvironment (TME) and the manner in which the immune system responds. While the location, composition, and density of TILs around melanoma cells favorably associated with survival and a lower risk of metastasis, their properties affect the prognosis [90].

In this scenario, the dominant immune cell groups in close proximity to melanoma cells include both CD8 + and CD4 + T-cells. However, recent research indicates that additional molecules might also have a potential correlation with prognosis. These molecules encompass the reduction of p16 expression, the transition between M2/M1 polarization in macrophages, as well as the levels of immune checkpoints, such as V-domain Ig suppressor of T-cell activation (VISTA) and PD-1 [91].

Melanomas have an abnormal overexpression of tumor-associated antigens (such as MART1/MLANA, MAGE antigens, and NY-ESO1), which makes them vulnerable to T-cell death as T cells that recognize such antigens are able to bypass negative thymic screening. The majority of skin malignancies, including BCC, malignant melanoma (MM), cSCC, and virus-negative MCC (VN-MCC), also have very high tumor mutational burdens (TMB), which are primarily triggered by UV-signature mutations and contribute to the development of novel tumor-associated epitopes, according to high-throughput sequencing techniques. The three solid tumors with the greatest TMB among those studied thus far are cSCC, VN-MCC, and melanoma. The notion that neoantigens significantly contribute to immunogenicity in the majority of malignancies is supported by the finding that TMB has proven a reliable predictor of immunotherapy response, both within and across tumor types [63, 92–94].

The immune system can also help in accelerating, modifying, and curing skin cancer [95]. The risk factor of cutaneous squamous cell carcinoma is enhanced rapidly in organ transplant patients including kidney, liver, heart, and diabetic nephropathy, etc. [96]. In one cohort study, it was found that during follow-up of patients with transplantation, 1,610 transplant recipients acquired 3,406 cancers. They included 668 patients with 2,231 SSC and 1,036 patients with 1,175 cancers other than SCC. Regardless of the kind of graft, the risk of cancer excluding SCC tripled after 20 years, whereas the risk of SCC remained consistent. Part of this increase in risk was caused by a subgroup of patients who were generating new SCCs at an accelerated rate [96].

### Indoor tanning

Indoor tanning is the crucial cause for the occurrence of non-melanoma and melanoma skin cancer together due to increased utilization of indoor tanning which is the major source of artificial UV radiation these days [97]. The use of indoor tanning is increasing in the mountain regions due to several health sequel, and among youth the use of indoor tanning beds is also on the rage, which takes up the cause of death-dealing melanoma and eye problems [98]. One of the systematic reviews from the International Agency for Research on Cancer clearly displayed a link between tanning bed use and a significantly higher risk of melanoma. This also led to the ban on artificial tanning beds for commercial purposes in Australia [99]. According to later research, banning sunbeds would prevent one out of six melanomas among Australians between 18 and 29 years of age [100].

### Age

Age is also a factor behind the occurrence of skin cancer i.e., the older population has more risk of occurring skin cancer than younger and middle age group persons. Aging is the culmination of all the changes that occur to people through time, including psychological, social, and physical changes. Aging is one of the most significant identified risk factors for the majority of human diseases, including cancer. Cells are more likely to experience somatic mutations after several generations of replication, which could result in unchecked cell growth [101]. As evidence, the incidence rate in women greater than 40 years of ag shows a linear increase in BCC incidence rates [84].

### Viral agent

There is various evidence that reveals that viruses also have a compelling or paramount role in the occurrence of skin cancer. In 1908, an experiment done by Ellerman and Bang, who were Danish scientists, demonstrated that the Rous sarcoma virus is the cause of the occurrence of leukemia in chickens. Afterward, there were many other observational authentication claims that viruses can also be the causal agent for skin cancer [102]. It has been established that skin infections mediated by β-HPVs have also been connected to cutaneous squamous cell carcinomas [103]. A viral origin is suggested by the greater frequency of keratinocyte carcinomas in people with compromised immune systems. The causal significance of HPV in the emergence of skin cancer is demonstrated through a transgenic mouse model. Skin cancer appears spontaneously in mice that express the HPV8 region and the human keratin-14 promoter simultaneously [76]. Although a meta-analysis appears to have proved that β-HPV infection is a risk factor for the development of SCC in healthy individuals, the role of β-HPV in the development of cSCC in the general population is still debatable [104]. The HHV8 human herpesvirus is the cause of Kaposi's sarcoma (KS). Age (risk increases with age) and immunocompromised status (induced by HIV, medications, or the host's genetic characteristics) are risk factors for the emergence of KS and HHV8-related malignant lesions [105].

### Oncogenic pathways

Cancer arises due to irregular cell growth and division, which lacks synchronization with the adjacent healthy tissues. This condition results from epigenetic and genetic changes that contribute to the transformation into a neoplastic state. Over recent years, numerous essential molecular pathways associated with the initiation, advancement, and proliferation of melanoma have been revealed.

#### WNT signaling pathway

The WNT signaling pathway governs critical processes, including determining cell polarity, cell fate, migration, and proliferation. The WNT protein family consists of glycoproteins that are secreted and bind to Frizzled receptor (FZD) proteins, which have seven transmembrane segments. This binding triggers intracellular signal transduction. Upon WNT-FZD binding, three potential signaling pathways become active: (a) a canonical pathway dependent on β-catenin; (b) a non-canonical pathway independent of β-catenin for cell polarity signaling; and (c) a pathway dependent on both WNT and protein kinase-C (PKC) [106, 107].

#### G-protein-coupled receptor-responsive skin cancer progression

All G-protein coupled receptors (GPCRs) share a common core feature consisting of seven alpha-helices that traverse the cell membrane. This structural arrangement enables them to be influenced by various agonists or antagonists. When GPCRs are activated, they typically oversee cellular functions by unleashing the signaling capabilities of inactive heterotrimeric G-proteins. These dormant heterotrimers comprise a Gα subunit bound to guanine diphosphate, which maintains a strong affinity for a functional Gβγ monomer. Upon being triggered by a compatible ligand or signal, a GPCR triggers the replacement of guanosine triphosphate (GTP) for guanosine diphosphate (GDP) on the Gα subunit. This results in the Gα subunit having a reduced affinity for Gβγ. Consequently, this modification leads to the separation of the subunits or reconfiguration of the heterotrimers, enabling Gα and Gβγ to interact with and regulate a diverse array of effector molecules, which is continuously expanding. Ultimately, the specific G-protein coupling of each receptor dictates the type of downstream signaling targets it engages with. Although GPCRs have conventionally been associated with the activities of specialized, non-dividing cells, they are also present in dividing cells, contributing to processes such as embryogenesis, tissue reconstruction, inflammation, angiogenesis, regular cell proliferation, and even cancer [108, 109].

#### MAPK pathway

The mitogen-activated protein kinase (MAPK) pathway holds significant importance as an intracellular signaling route. Activation of this pathway takes place in normal physiological conditions when growth factors externally bind to receptor tyrosine kinases (RTKs).

The role of MAPKs is to facilitate the transmission of signals that regulate various intracellular processes, including immediate hormonal responses, embryonic development, cellular differentiation, proliferation, and programmed cell death (apoptosis). For the MAPK pathway to be activated, an interaction between an RTK and its corresponding ligand is crucial. This interaction triggers the activation of rapidly accelerated fibrosarcoma (RAF) components (BRAF, ARAF, and CRAF), which are integral to the pathway [110]. This series of events set off a chain reaction within the cell, resulting in increased cellular growth, improved cell survival, and the prevention of apoptosis. In the absence of an RTK-ligand interaction, RAF kinases remain in their inactive state without undergoing phosphorylation, leading to no signal transduction. However, when an RTK and ligand interact, the phosphorylation of RAF takes place. This leads to the activation of BRAF and CRAF serine/threonine kinases, which then act as downstream mediators. Once activated, RAF forms homo- or heterodimers that interact with and phosphorylate mitogen-activated extracellular signal-regulated kinase (MEK). MEK, in turn, phosphorylates and activates mitogen-activated extracellular-signal-regulated kinase (ERK). The activation of ERK plays a pivotal role in oncogenesis by promoting cell growth and differentiation. Additionally, activated ERK is responsible for initiating a negative feedback loop at various levels of the MAPK pathway to regulate its activity. Furthermore, RTK-ligand interactions can also trigger the activation of other intracellular pathways, such as the phosphatidylinositol-3-kinase (PI3K) pathway [111, 112].

#### PI3K pathway

Oncogenic RAS, a participant in the MAPK pathway as discussed earlier, also functions as a positive regulator upstream of the PI3K pathway. Consequently, the activation of RAS transcription triggers the downstream activation of two distinct yet interconnected pathways such as the PI3K-AKT signaling pathway and the RAF–MEK–ERK pathway. While both pathways contribute to cell proliferation, dissemination, and survival, the PI3K-AKT pathway additionally stimulates anabolic processes, while the RAF–MEK–ERK pathway predominantly facilitates proliferation and invasion. One of the inhibitors of the PI3K pathway is the enzyme phosphatase and tensing homolog deleted on chromosome 10 (PTEN). PTEN catalyzes the de-phosphorylation reaction of phosphatidylinositol 3,4,5-trisphosphate (PIP3) back to phosphatidylinositol 4,5-bisphosphate (PIP2) through its lipid phosphatase activity. This action leads to a decrease in phosphorylated AKT (p-AKT) levels and subsequent inhibition of the PI3K signaling pathway. Additionally, PTEN has the ability to target and dephosphorylate other proteins, such as focal adhesion kinase (FAK), which in turn curbs focal adhesions and reduces cellular migration. Interestingly, PTEN is also believed to interact with the MAPK signaling by dephosphorylating adapter proteins, resulting in diminished phosphorylation of MEK1/2 and ERK1/2. The loss or inactivation of PTEN leads to unregulated signaling through the PI3K pathway. Notably, the absence of tumor suppressor genes located on chromosome 10, including PTEN, is detected in a considerable portion (30%–60%) of non-inherited melanomas. Crucially, the lack of PTEN expression has been noted in around 30%–50% of established melanoma cell lines and 5%–20% of primary melanomas. Remarkably, mutations in both BRAF and PTEN have been frequently observed to co-occur at high rates in melanoma, whereas NRAS mutations have been found to be mutually exclusive with BRAF and PTEN mutations [113–115].

## Solid resistant skin tumor mechanism

Solid tumors start at the site where the level of oxygen is frequently reduced due to the enhancement in oxygen diffusion interspaces i.e., the distances among cells and vasculature. The oxygen deficiency also occurs due to additional uptake of oxygen by the proliferative cancerous cell which causes hypoxia in the surrounding environment which accelerates the mechanism of cell apoptosis [116]. At the tumorous site, the accumulation of mast cells occurs to tackle the damages and impairment. Mast cells recruit various kinds of chemokine behaviors which play a crucial role in defensive mechanisms [117].

**Fig. 4:**
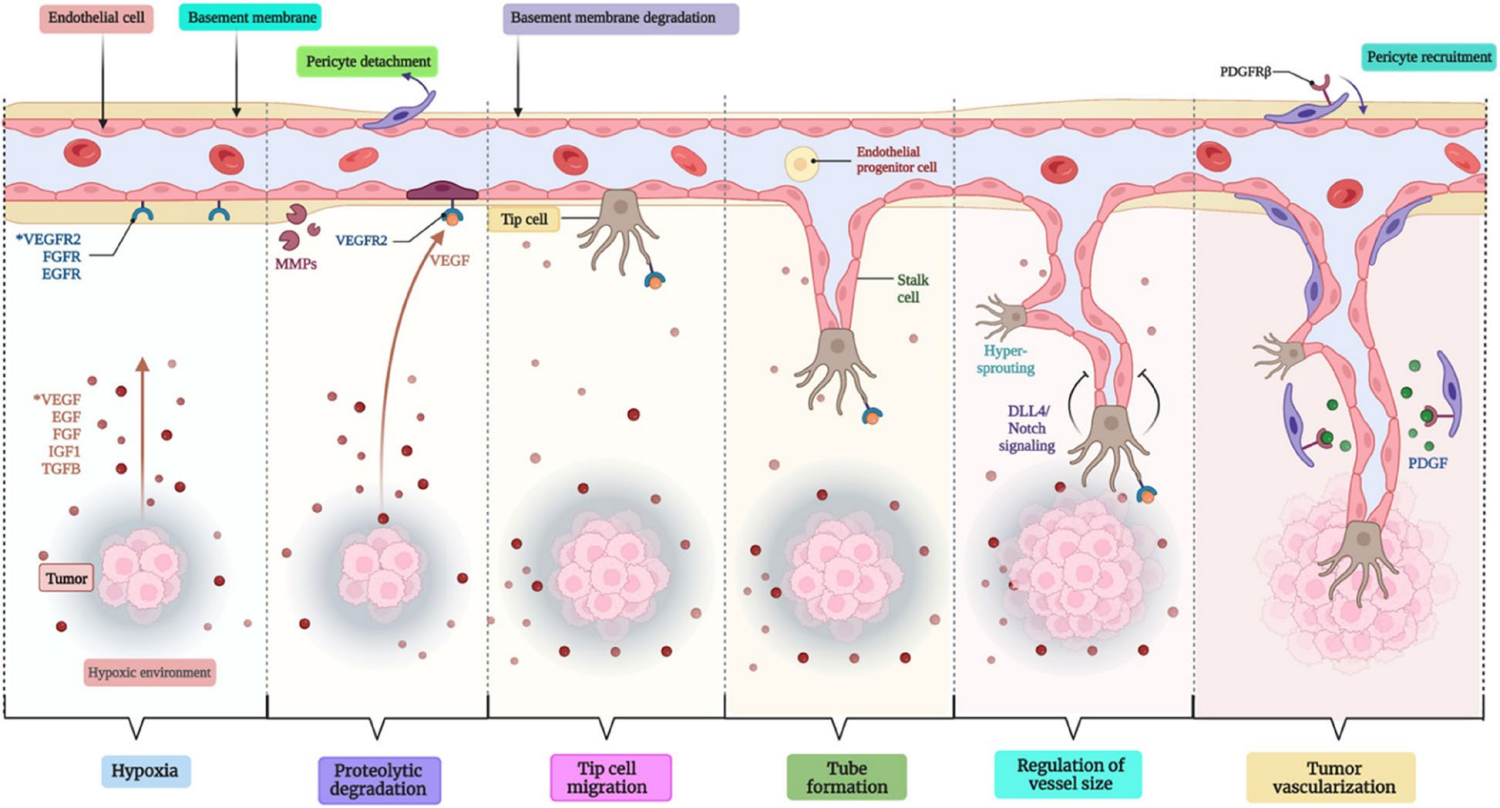
Diagrammatic representation of the detailed mechanism of tumor vascularization

SCC or BCC expands as a precancerous lesion named actinic keratosis, which is also called keratinocyte intraepidermal neoplasia, very general in UV-exposed regions and looks like reddish macules which are further enfolded by yellowish scales. There is the presence of a typically large nucleus including hyperchromasia, dyskeratosis, and mitosis at the region of actinic keratocytic in the epidermal layer. The pathogenic pathway which shows involvement in skin cancer is shown in Fig. 4 as such; Protein patched homolog 1 (PTCH1) plays the tumor-conquering action which encodes a protein receptor termed Sonic Hedgehog (SHH), which causes the loss of PTCH1 functionalities that impacts on working of G-Protein coupled receptor as decreasing the suppression of intracellular signaling which Smoothened (SMO) G-Protein coupled receptor. glioma-associated oncogene transcription factors (GLI) were targeted by SMO, whether a mutation in PTCH1 induced encouraged activation of target genes [118].

## Approaches to combat skin cancer

We must prevent skin cancer in various ways which must be effective in controlling and stopping the spreading and rising level of cancer patients, such as (i) By increasing the awareness of sun exposure and its harmful effects in public and guiding the patients on an individual basis [119], (ii) By avoiding indoor tanning and never use UV beds, (iii) By applying broad spectrum range of sunscreen having a Sun protection factor (SPF) range equal to or greater than 15 [120], (iv) By avoiding to getting sunburned, (v) By examining your whole body from head to toe at every month, (vi) By visiting the dermatologist at least one in a year for examining the skin professionally, (vii) By being in shade mostly between 10 AM to 4 PM [121]. Treatment is depended upon the type and stage of skin cancer and the circumstances by which it can be diagnosed. There are different treatments regimes that are either under trial or approved by US-FDA which tabulated in Table 1.

**Table 1:** The approved and currently under trials treatment modalities for the cSCC. The Table was modified from Mårten et al. (https://www.nature.com/articles/s41568-023-00583-5)

**Treatment options**	**Description**	**Manifestation**	**Approved**	**Reference**
**Conventional Treatment/Therapies**				
Excision	Surgical removal of cancer cells	Localised cSCC	Yes	[122]
Mohs	Progressive resection of tissues layers containing cancer cells by minimizing the removal of healthy tissues	Localised cSCC	Yes	
Cryotherapy	Freezing and destroying the tumor by applying liquid nitrogen	Superficial; Localised cSCC	Yes	
Photodynamic	The initiation of tumor-ablation reaction by applying light source with therapeutics	Localised cSCC	Yes	
Radiation	High-energy radiations	Locally advanced, recurrent, or metastatic cSCC	Yes	
Topical with therapeutics	Application of chemotherapeutics (5-flurouracil or imiquimod)	Superficial; Localised cSCC	Yes	
Systemic chemotherapy	Single or combination therapies of different chemotherapeutics such as cisplatin and 5-flurouracil	Locally advanced, recurrent, or metastatic cSCC	Yes	
**Systemic Targeted Therapy**				
Gefitinib	EGFR tyrosine kinase inhibitor	Locally advanced, recurrent, or metastatic cSCC	No	[123]
Lapatinib	EGFR tyrosine kinase inhibitor	Locally advanced, recurrent, or metastatic cSCC	No	[124]
Erlotinib	EGFR tyrosine kinase inhibitor	Locally advanced, recurrent, or metastatic cSCC	Yes, for NSCLC	[125]
Panitumumab	Monoclonal antibody that targets the extracellular domain of EGFR and inhibits it	Locally advanced, recurrent, or metastatic cSCC	No	[126]
Cetuximab	Monoclonal antibody that competitively inhibits the EGFR	Locally advanced, recurrent, or metastatic cSCC	Yes, for HNSCC	[125]
**Systemic Immunotherapy**				
Cemiplimab	PD1 checkpoint inhibitor	Locally advanced,recurrent, or Metastatic cSCC, not curable with surgery or radiation therapy	Yes	[127]
Nivolumab	PD1 checkpoint inhibitor	Locally advanced, recurrent, or Metastatic cSCC, not curable with surgery or radiation therapy	Yes	[125]
Pembrolizumab	PD1 checkpoint inhibitor	Locally advanced, recurrent, or Metastatic cSCC, not curable with surgery or radiation therapy	Yes	[128]
Ipilimumab	CTLA-4 checkpoint inhibitor	Locally advanced, recurrent, or Metastatic cSCC, not curable with surgery or radiation therapy	yes	[125]
**Combination therapies**				
Cetuximab + 5FU + Cisplatin	EGFR inhibitor + Chemotherapeutic agents (Cetuximab + 5FU + Cisplatin) Or 5FU + Cisplatin	Platinum-resistant metastatic SCC	Yes, for HNSCC	[129]
Pembrolizumab + chemotherapy	PD1 checkpoint inhibitor + Chemotherapeutic agents	Metastatic or locally advanced cSCC,	Yes, for HNSCC	[130]
**Intralesional therapy**				
5 Fluorouracil	Anti-cancer/cytotoxic effects	Localised, early stage cSCC	No	[131]
Methotrexate	Anti-cancer/cytotoxic effects	Localised, early stage cSCC	No	[132]

Abbreviations: These treatments modalities are currently in clinical use a part of different trials or an approved modality in the United States

*cSSC* cutaneous squamous cell carcinoma, *PD1* Program Death 1, *EGFR* Epidermal growth factor receptor, *NSCLC* Non-small cell lung cancer, *HNSCC* Head and neck squamous cell carcinoma, *CTLA-4* Cytotoxic t-lymphocyte associated protein-4

### Surgery

Surgery is the method in which the cancer cells are treated with a combination of chemotherapeutics, and excisional removal of tissues with the use of certain surgical instruments are used for the procedure. The traditional method of removing cancerous cells by using a scalpel to a certain depth is also a type of surgery. Different kinds of surgical methods are used to treat cancerous tumors which are described as such [133].

#### Simple excision

In simple excision, the area belonging to the tumor are totally cut and removed along with some normal cells or tissues of the body followed by stitching of the area of the incision, then the removed tissues by a dermatopathologist, were sent to the laboratory for assessment the tissues for the confirmation that the whole tumor area has been removed [134]. A markup in fusiform shape is advisable which precludes the excess skin along the ablation [135].

#### Mohs micrographic surgery

**Fig. 5:**
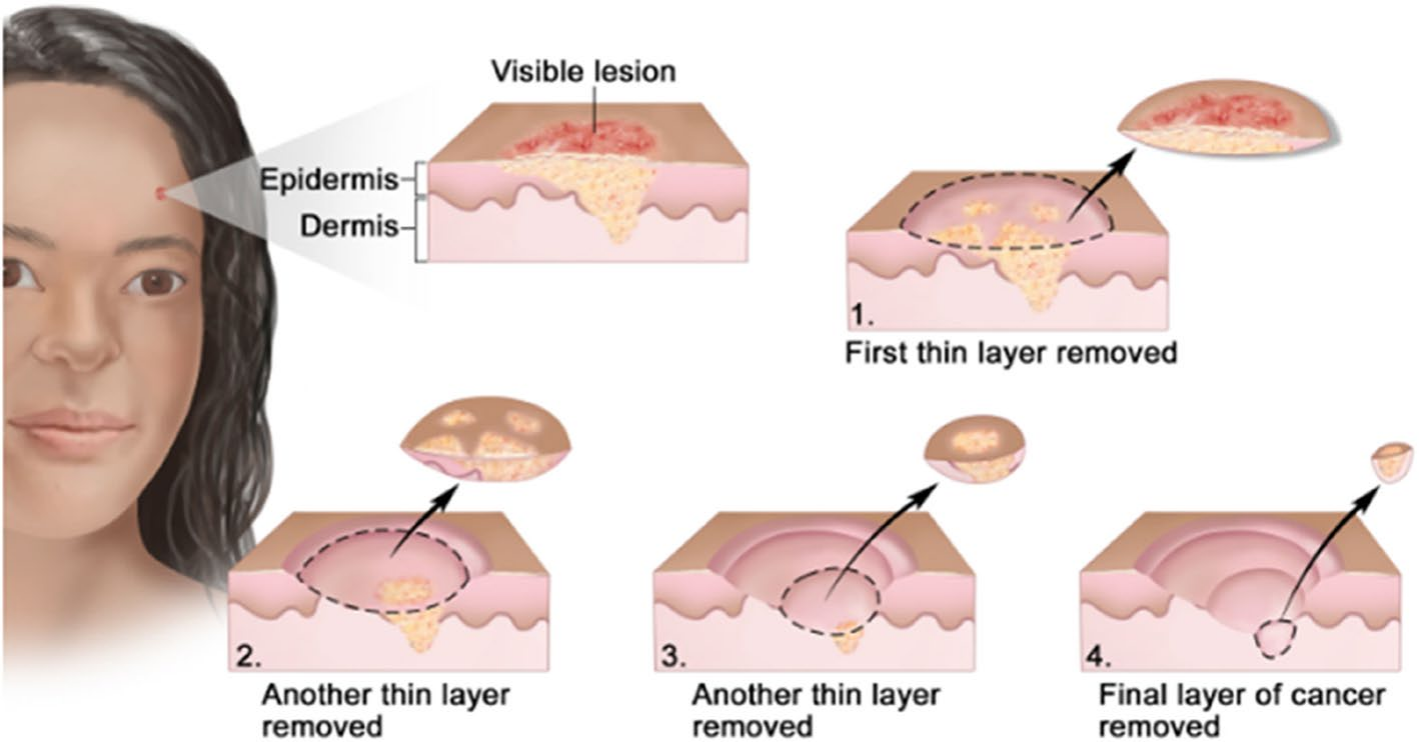
Procedure for Mohs Microscopic surgery used for the removal of the tumor from the skin. Adapted with permission from [141]

It is the treatment of choice for high- risk nonmelanoma skin cancer. It is usually performed when the cancer is recurrent type and when tissue conservation is needed. The surgery is an outpatient one, which is preferentially used for large area tumors which is the amalgamation of the removal of the tumorous tissue followed by microscopic analysis during the surgery takes place. By analyzing the skin in stratified form the little damaged skin is also affected during the removal of the tumor (Fig. 5) [136, 137]. And further, the area is assessed for the presence of any additional tumor tissues or cells at the excised location [138]. After that, a thin portion of the underlying tissue is extracted and histologically analyzed by a frozen section to see if it is tumor-free. If not, additional surgical layers are excised until no microscopic signs of a tumor are present. The patient stays in the hospital while tissue samples are examined following the one-time surgery. The fundamental benefit of Mohs surgery is that it maximizes the preservation of healthy tissue while providing exact microscopic control of the whole tumor margin. Retrospective investigations have discovered a 97% 5-year primary tumor cure rate and a 90% 5-year recurrence cure rate. This contrasts with 77% for recurrences and 92% for primary tumors with other methods [139, 140].

#### Electrodesiccation and Curette (ED&C)

In this technique, the health specialist removes the tumor area after desensitizing the area by applying anesthetic locally and sweeping out the tumor tissues with an instrumental tool called ‘curette’ which has sharp edges and a tiny scoop. Then, to stop any further bleeding, they use a probe by using electricity or electric current. Following that, one has to follow the same procedure several times which is followed by bandaging the treated area which leaves a light pink or white scar. This technique is used only for the tumor which lies on the upper layer of the epidermis mostly for BCC and SCC [134]. A controlled study compared the cure rates of low-risk SCC at one year by ED&C to those by surgical excision in a dermatological clinic at a Veterans Administration teaching hospital. A second study looked at the success rate of curettage and electrodesiccation for low- risk lesions in a private clinic. The first study did not show any significant difference between ED&C ( whole of 14 patients treated successfully) and Surgical excision (15 of 16 patients treated successfully and 1 recurrence), while the second study showed that 106 of 106 patients were treated successfully by ED&C as compared to 95% arbitrary cure rate [142]. This proved the efficacy of the ED&C method in the treatment of low-risk BCC.

### Phototherapy

#### Photothermal therapy

**Fig. 6:**
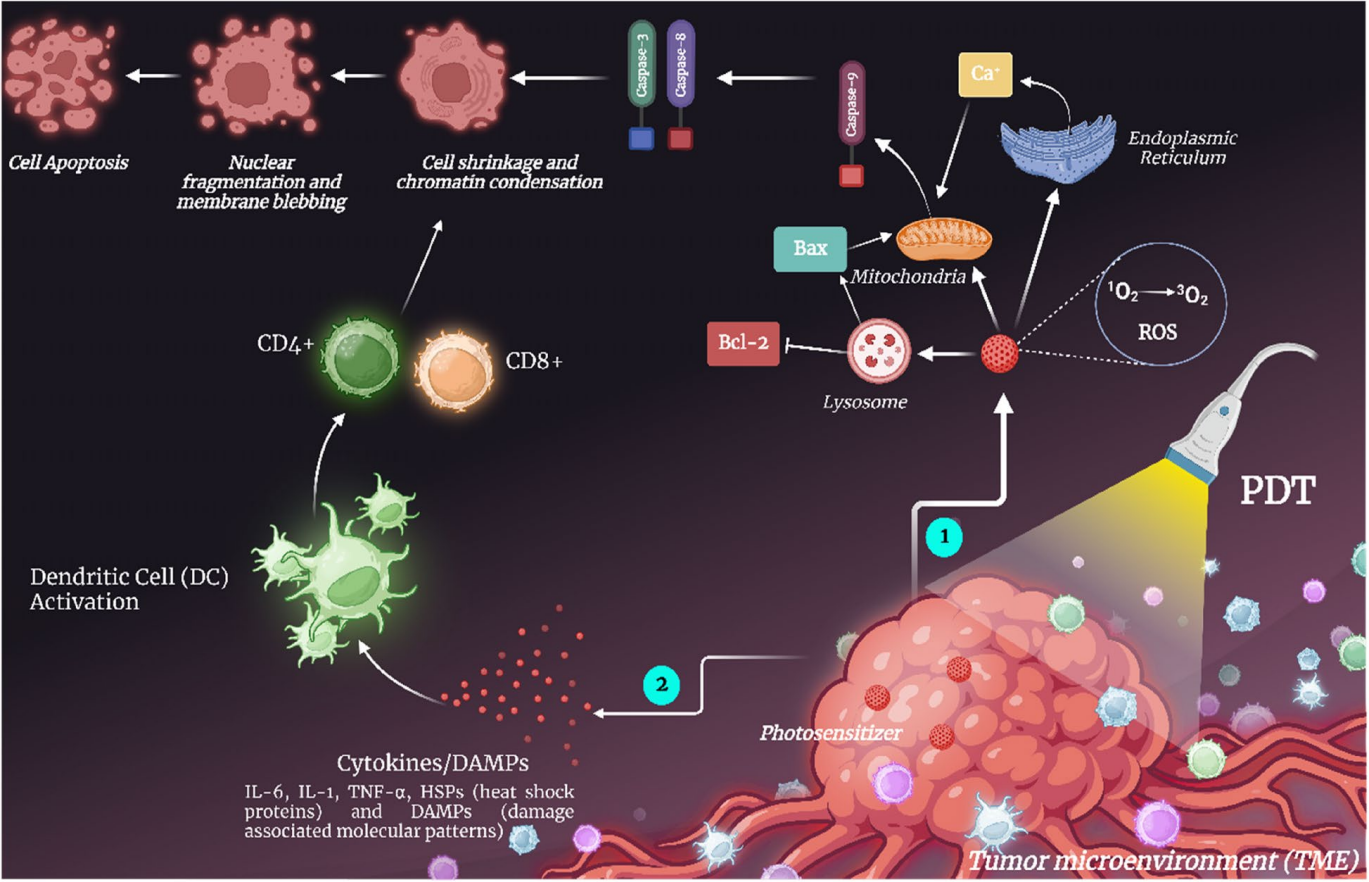
An illustration of the effector mechanism in photodynamic therapy. 1) Activation of the photosensitizer by external light of a specific wavelength results in the singlet state, which is followed by the creation of reactive oxygen species (ROS), which is the main cause of the apoptosis of tumor cells. 2) Photodynamic therapy activates the immune system, releasing inflammatory mediators like IL-6, IL-1, and TNF-alpha, heat shock protein (HSP), and DAMPs (Damage Associated Molecular Patterns), which cause tumor cells to die

It is a technique in which an inactivated topical medication is applied to the affected area, and the area gets further activated by exposure to a certain wavelength of light. The main component of photodynamic therapy (PDT), a way of treating malignancies, particularly cancer, is the impact that photochemical reactions have on biological tissues. The initiator of these processes is light energy [2]. The three key elements of PDT are oxygen, light, and photosensitizer. It is clear that none of these substances are harmful when taken alone, but when combined, they start a photochemical process that results in the production of singlet oxygen, a highly reactive substance, as well as a number of free radicals. By causing profound functional and structural changes in the cellular membrane and activities taking place inside of them, this singlet oxygen activates the cytotoxic activity, which is a unique mechanism of damage to critical cell functions and ultimately leads to the death of these cells [143] as shown in Fig. 6. In the instance of skin cancer the drug may be applied directly on the skin in liquid form [144]. Photodynamic therapy and photothermal therapy are two different kinds of therapy for healing skin cancer in which the former results in chemical damage at the localized target site whereas the latter results in thermal damage at the localized cancerous lesson site. Photosensitizers play a key role in photodynamic therapy due to killing or inactivating cancerous cells. Whereas, in addition, the exogenous photothermal contrast agents are not having any important role but can enhance the efficacy and efficiency of the therapy [145]. A randomized intraindividual comparison study to assess the effectiveness and safety of methyl amino levulinate (MAL)-PDT vs. Imiquimod (IMQ) 5% in the treatment of patients with field alterations to prevent the development of new NMSCs was carried out. It was found that the trial was completed by 44 patients. There was no statistically significant distinction in the creation of new NMSCs at any stage during the follow-up period between MAL-PDT and IMQ, hence there was no difference with regard to the primary endpoint. Both treatment plans were well-tolerated and safe. MAL-PDT was preferred by patients in relation to the technique, response rates, and future options [146]. For its members, the American Society of Dermatologic Surgery (ASDS) regularly creates consensus documents on a range of topics concerning surgeries related to dermatology. Photodynamic treatment (PDT) has undergone numerous advancements, and it is now widely used to treat a range of skin disorders. According to the ASDS, photodynamic treatment is quite efficient for treating inflammatory acne vulgaris, precancerous lesions, and superficial nonmelanoma skin malignancies [147].

##### Clinical applications of photodynamic therapy (PDT)

Various studies have recorded the use of PDT to be an efficacious treatment for BCC [148, 149]. For low-risk basal cell carcinoma (BCC), photodynamic therapy (PDT) is a well-established treatment option [150]. Many nations have approved topical PDT as a therapy for BCC. Although it has not yet received USA approval. A recent evaluation found interest in its use as a "off-label" treatment. ALA and MAL are approved prodrugs for the topical PDT therapy of BCC [151]. Despite reports that the usage of ALA and MAL cause different pain levels, with MAL-PDT causing less pain, a cohort study revealed no discernible difference [152]. With a three-month follow-up, the licensed MAL-PDT protocol employs a cycle of two treatments separated by a seven-day gap [150, 153]. The suggestion to consider a treatment with topical PDT is often restricted, i.e., in the case of nBCC, to thin (2 mm) nBCC, because the ability to penetrate both the prodrug and light decreases with the growing depth of the BCC lesion [153].

As topical PDT has a lesser clearance rate than surgical BCC treatment and has a limited depth of penetration for both the prodrug and the stimulating light, topical PDT is not advised for high-risk BCC subtypes [148]. However, in some circumstances, if a low-risk BCC subtype spreads periphery from a high-risk subtype, topical PDT may serve as an adjuvant therapy to surgery. PDT treatment may be utilized before surgery in order to streamline the surgical procedure or, more frequently, after margin-controlled surgery in cases where a low-risk BCC region remains and the morbidity from further surgery outweighs the risk of a potential recurrence after PDT [154, 155].

PDT treatment is constrained by the radiation's depth of penetration, which varies with the BCC's depth and pigmentation as well as with the radiation's wavelength. Some of the recent photosensitizers can expand the PDT treatment window into the near-infrared (NIR), potentially opening the door to deeper tissue penetration. They can also penetrate pigmentation, which reduces the PDT treatment window's effectiveness by quenching ROS produced by PDT and by absorbing incident radiation [150].

The results of various clinical randomized trials as mentioned in a systematic review [156] shows that the use of topical PDT for low- risk BCC is an effective treatment. For nBCC there were 3 trials with MAL prodrug and it showed a clearance rate of 73% [157], 91% [158, 159], and 88% [160] after 3 years. One study was for nBCC with ALA and it showed 93% clearance after 3 years [161, 162]. Three studies were for sBCC which showed clearance at three years of 90% [163], 87% [164], and 82% [165–167].

A clinical trial (NCT03573401) titled “A Randomized, Double-Blind, Vehicle-controlled Multicenter Phase III Study to Evaluate the Safety and Efficacy of BF-200 ALA (Ameluz®) and BF-RhodoLED® in the Treatment of Superficial Basal Cell Carcinoma (sBCC) with Photodynamic Therapy (PDT)” is being conducted at multi-centers (15 sites) to compare the safety and effectiveness of photodynamic therapy (PDT) for superficial basal cell carcinoma (BCC) using the drug Ameluz® and the PDT-lamp BF-RhodoLED® to the corresponding placebo treatment [168]. This kind of trial would be the perfect opportunity to conduct additional research on any refractory or recurrent tumors that may develop and to correlate these findings with patient characteristics so as to both improve the treatment of such tumors and, possibly, screen patients before they undergo PDT and provide them with personalized information about the possibility of treatment failure. By doing so, PDT's cosmetic advantages may be made more publicly known and new knowledge about the technique's potential use to the treatment of others, more dangerous tumors could be gathered.

##### Squamous cell carcinoma

The absence of standardized treatment procedures or results data makes it difficult to employ PDT for SCC clinically. Although MAL and red- light illumination have been utilized in the majority of published trials for SCC, ALA and blue-light illumination (ALA-PDT) is the most popular method of PDT administration in the United States. Furthermore, it is unknown if additional tumor or therapy factors influence PDT response. Data on whether tumor size [169–172] or anatomic location [173, 174] affect response are inconclusive. According to varying reports, the number of PDT treatments and the incubation period for ALA both have an impact on the removal of malignant and premalignant lesions [169, 175, 176].

In 58 patients with 68 primary SCCis lesions that were treated with ALA-PDT and blue light illumination, according to a retrospective review. The initial full response rate following PDT was 77.9%; it was unrelated to the quantity of PDT treatments. The position on the face, a tumor diameter of less than 2 cm, and a longer ALA incubation period were significantly linked with the response on multivariate analysis. Lesions treated with an incubation period of no more than three hours exhibited a 53.3% response, while extended incubation times resulted in an 84.9% response. SCCis recurred later in 7 of 53 instances (13.2%), with a median interval of 11.7 months [177].

Repeated cycles of PDT have been proposed to prevent the development of carcinomas in patients at high risk with many lesions. Field therapy of lesion precursors with topical fluorouracil has been found to lower the incidence of eventual SCC. In this way, PDT may address preexisting SCCis and prevent new lesions, however, further research is needed to fully understand this effect [178, 179].

##### Melanoma

PDT is a possible alternative palliative therapy for individuals with advanced melanoma since it is minimally invasive and has few adverse effects. The most important challenge is to overcome melanoma resistance, due to melanosomal trapping, the presence of melanin, enhanced oxidative stress defense, defects in the apoptotic pathways, immune evasion, and neo-angiogenesis stimulation [180]. Chemotherapy, immunological treatment, or a photosensitizer that is designed for a theranostic approach with increased efficacy can all be used in conjunction with PDT to treat melanoma.

Baldea et. al. reported that while PDT on human and mouse melanoma cells using various procedures significantly increased apoptosis, necrosis, tumor growth halt, and prolonged animal life in experimental settings, it rarely resulted in full recovery and/or was accompanied by recurrence and side effects. According to the clinical data, PDT caused choroidal melanoma and cutaneous melanoma metastases to regress [180].

An article demonstrated a different method for treating melanoma by combining traditional PDT with electroporation. Through temporary membrane holes, the method improved the photosensitizer's intracellular trafficking. In vitro, the technique proved more effective than PDT alone at inducing apoptosis in human melanoma cell lines that were both amelanotic (C32) and melanotic (MeWo) [181].

A few research [182] suggested theranostic nanoparticles to treat melanoma. A complex nanoconstruct with increased tumor targeting and anti-melanoma activities against a melanoma cell line (A375) in vitro was made using a single-walled carbon nanotube carrier and zinc monoamino phthalocyanine, which was additionally connected to folic acid [183]. Aerosol OT (AOT)-alginate nanoparticles were used as a carrier to construct a multidrug nanoparticle that delivered doxorubicin, a chemotherapeutic agent, and methylene blue, a photosensitizer for PDT. The melanoma cells' drug accumulation, as well as the antitumor effectiveness, were both raised by the nanoparticles [184]. Fraix et al. reported using two chromo fluorogenic components to visualize the medication administration while also delivering photosensitizers to melanoma cells using biocompatible polymeric nanoparticles. Through the production of both singlet oxygen and nitric oxide, this method improved tumor cell killing [185]. Clinical studies revealed that topical IMQ (a toll-like receptor agonist) and PDT (ISPI) together enhanced PDT's overall anti-melanoma effects [186–188]. According to current applied research, actively targeted PDT unconventional treatments do seem to have a chance of enhancing treatment for metastatic melanoma.

#### Clinical applications of Photothermal therapy (PTT)

In the fight against cancer, photothermal therapy (PTT), which transforms light energy into heat energy, has emerged as a new research hotspot. PTT's simple operation, brief treatment period, and quick recovery make it highly valuable for research [189, 190]. Following administration of the photosensitizer for a variable amount of time, light of a fixed wavelength is focused precisely on the target lesion, which causes the selective excitation of the photosensitive agents and the subsequent induction of photophysical and photochemical actions for the treatment of cancer.

In PTT, photothermal agents accelerate the localized heating of cells and tissues. These substances absorb energy from photons and change from their ground singlet state to an excited singlet state when exposed to the light of a particular wavelength. A heat-shock reaction begins when a tissue's temperature rises to 41 °C, which then triggers a series of quick changes in gene expression patterns, including the production of heat-shock proteins, to lessen the effects of the original thermal injury. When the temperature rises to 42 °C, irreversible destruction of tissue takes place; after being heated for 10 min to a temperature between 42 °C and 46 °C, tissues experience cell necrosis. Cells quickly perish at 46–52 °C due to microvascular thrombosis and ischemia. Due to protein denaturation and plasma membrane disintegration, cell death occurs very instantly at tissue temperatures > 60 °C, which are often reached with PTT [191, 192]. Due to the fact that cancer cells often have a low tolerance to heat, PTT is a promising treatment. Additionally, external laser irradiation with a dosage that may be adjusted enables the selective eradication of different malignancies while minimizing harm to the nearby nonmalignant tissue [193].

Although other delivery methods are possible, such as oral delivery (for example, ALA, which is more convenient for patients but is associated with concerns over inter-patient variability in bioavailability and pharmacokinetics, photosensitizing agents are typically administered in clinical settings through intravenous or topical application. The pharmacokinetics and biodistribution of photosensitizers have been demonstrated to vary depending on whether they are delivered intravenously or intraperitoneally in preclinical investigations on rodents [194].

Large-scale clinical trials using photothermal therapy (PTT) agents, which can be utilized to boost the effectiveness of localized light-based heating and ablation of cancer tissues, have not yet been conducted; laser ablation without PTT drugs has, however, been used therapeutically [145].

The biggest challenge with PTT is the systemic diffusion of PTAs in the body and non-precision laser irradiation, which might have detrimental side effects on healthy tissues surrounding tumors [195]. Attention has been focused on the creation of nanosized photothermal agents (NPA), which can accumulate in tumors through enhanced permeability and retention (EPR) effects and active targeting [196, 197]. This would thus reduce the off- site side effects. To present, numerous NPAs, including metal nanomaterials (gold and platinum), semiconductor nanomaterials (copper), nanomaterials made of carbon (graphene and carbon nanotubes), and conduction of polymers (polyaniline and polypyrrole), have been created for improved PTT-based cancer treatment [198–200].

Han et. al. prepared bimetallic hyaluronate modified gold-platinum nanoparticles for photothermal therapy of skin cancer. It was found that these nanoparticles were noninvasively transported into deep tumor tissues in comparison to traditional PTT through injection, and they ablated the targeted tumor tissues by NIR light irradiation [201]. In another study, nanographene oxide- hyaluronic acid conjugate was used for photothermal ablation therapy for melanoma skin cancer. The study confirmed the feasibility of using transdermal photothermal ablation therapy using this conjugate [202]. Bonamy et. al. studied the internalization, trafficking, and efficiency of green gold nanoparticles (gGNP). The study showed that glucose-stabilized gGNP allows for fast internalization, which is four times higher in malignant cells in comparison to healthy cells with no side effects. This finding is particularly pertinent to the targeting and treatment of cancer. These were also found to be less cytotoxic than gold nanoparticles [203].

### Immunotherapy

Immunotherapy is a novel approach in the field of cancer treatment that garners antigen–antibody interactions. Interferon plays a great role in immunotherapy to treat cancer and act on target cells by binding with receptors that are present on target cells. Interferons produce antiproliferative effects (by inhibiting mitosis and growth factor, by activation of pro-apoptotic genes, and by promoting antiangiogenic activity) on cancer cells and also support the immune system to fight against cancer-causing agents [204]. A blinded prospective cohort study was performed where a 4-day regimen of topical calcipotriol and 5-FU was contrasted with a control regimen of Vaseline and 5-FU to treat Actinic keratosis (AK), which is a predecessor of squamous cell carcinoma (SCC). After 1, 2, and 3 years of trial, the incidents of SCC and BCC were accessed. It was observed from the study that the probability of developing SCC within three years of treatment is greatly reduced by a brief course of calcipotriol with 5-FU treatment on the face and scalp. This treatment is connected with the generation of strong T cell immunity and Trm production against AKs [205]. In a randomized trial using Nivolumab plus ipilimumab or nivolumab alone. It was suggested that patients with advanced melanoma who got nivolumab + ipilimumab or nivolumab alone had a higher percentage of sustained long-term survival rates and progression-free survival at 5 years than patients who were given ipilimumab alone, with no discernible decrease of quality of life in the nivolumab-containing regimens [206].

**Fig. 7:**
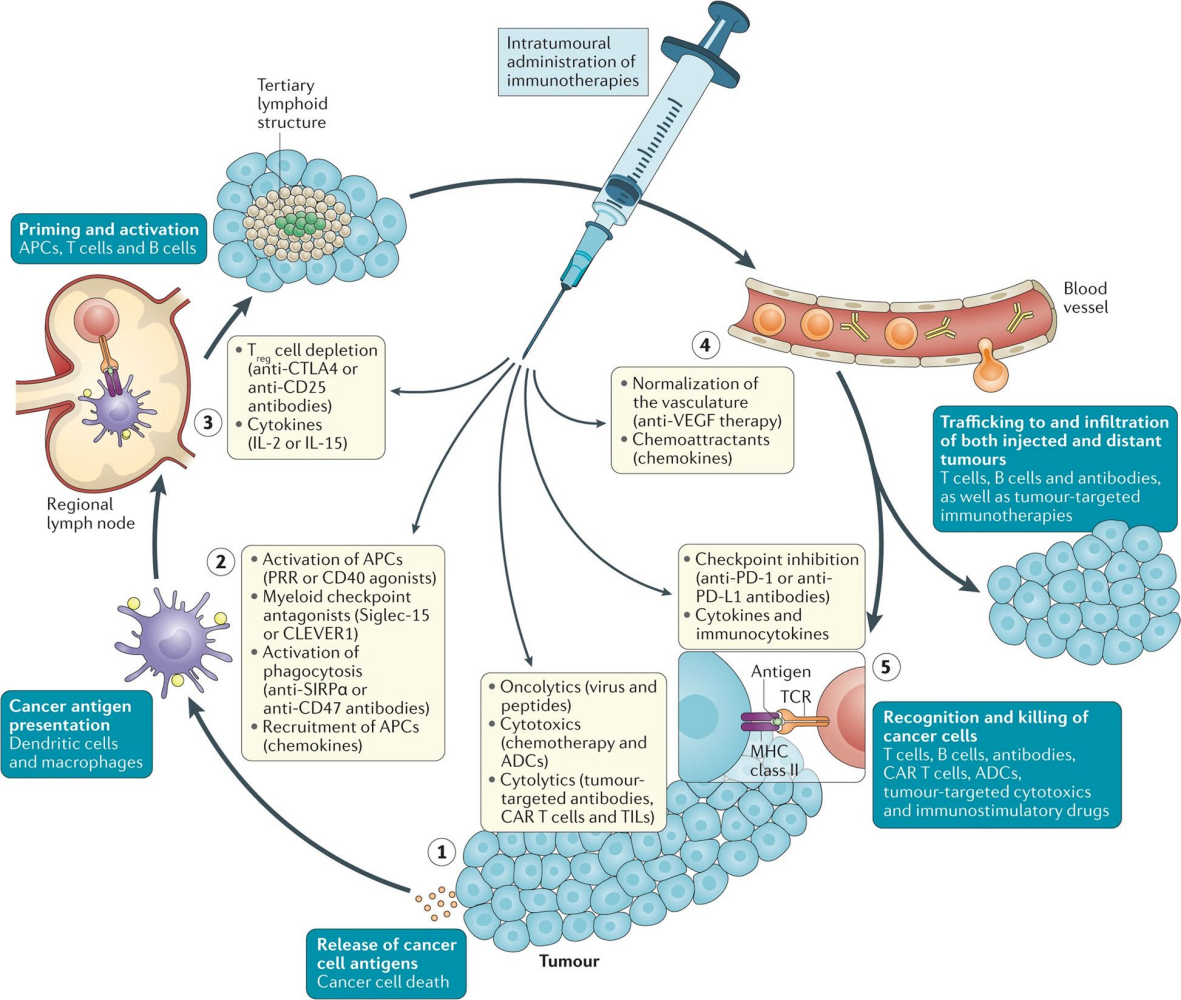
Illustration of cell types, processes, immunotherapy, and their effect on the tumor after intratumoral administration. Adapted with permission from [207]

Ignacio et. al. researched the effect of intratumoral administration of cancer immunotherapeutics against tumor cells. The aim of the study was to evaluate the efficacy of the monothematic agent for reducing the tumor after intratumoral local administration as shown in Fig. 7. The study results revealed that the direct tumoral administration of the iontophoretic entity causes increased efficacy or therapeutic index due to bypassed systemic exposure of the immunotherapeutic agent as well as drastically changes the toxicity profile of the immunotherapeutic agent [207].

#### Types of Skin Cancer Immunotherapy

- Checkpoint Inhibitors: These drugs block certain proteins that inhibit the immune response, allowing immune cells to recognize and attack cancer cells more effectively. Checkpoint inhibitors like PD-1 (programmed cell death protein 1) and CTLA-4 (cytotoxic T-lymphocyte-associated protein 4) inhibitors are commonly used in skin cancer treatment.
- Cytokine Therapy: Cytokines are signaling molecules that regulate the immune system. Interferons and interleukins are examples of cytokines used in skin cancer immunotherapy to enhance immune responses against cancer cells.
- Cancer Vaccines: These vaccines stimulate the immune system to recognize and attack cancer cells. In skin cancer, therapeutic vaccines can target specific antigens expressed by cancer cells, boosting the immune response against them.
- CAR-T Cell Therapy: Chimeric Antigen Receptor (CAR) T-cell therapy involves genetically modifying a patient's T cells to express receptors that target cancer-specific antigens. This personalized approach has shown promise in certain types of skin cancers.

#### Targets and ongoing studies

- PD-1/PD-L1 Inhibitors: Pembrolizumab and nivolumab are PD-1 inhibitors approved for advanced melanoma treatment. They target the interaction between PD-1 on T cells and PD-L1 on cancer cells, enabling immune cells to attack the cancer. Ongoing studies explore their combination with other therapies and their effectiveness in different stages of melanoma. (NCT02506153) compares the effectiveness of pembrolizumab versus high-dose recombinant interferon alfa-2B in treating patients with stage III-IV melanoma that has undergone surgical resection but are likely to recur or spread. High-dose recombinant interferon alfa-2B may aid in melanoma reduction or growth inhibition [208].

Anti-PD-1/PD-L1 therapy for skin cancer is revolutionizing management and results. The higher anti-PD-1/PD-L1 response rates in skin cancer relative to other solid tumor types are probably caused, at least in part, by a greater mutational burden. These drugs have 40–60% response rates when used as monotherapies, with many of those responses lasting a long time [209].

b) CTLA-4 Inhibitors: Ipilimumab is a CTLA-4 inhibitor used in advanced melanoma treatment. It enhances T cell activity by blocking CTLA-4, a protein that suppresses immune responses. Ongoing research investigates its use in combination with PD-1 inhibitors and its efficacy in preventing disease recurrence.

Ipilimumab boosts the immune system's reaction to cells of melanoma and tumors by inhibiting CTLA-4. The medication acts to stimulate T lymphocytes, allowing them to grow and destroy melanoma cells wherever they are found in the body [210]. This is an oncolytic virus therapy approved for advanced melanoma. It is injected into tumors that triggers immune responses against cancer cells. Studies focus on its application in combination with checkpoint inhibitors and in earlier stages of melanoma. It is used to treat melanoma skin cancer that is unresectable because it has spread to other parts of the skin, soft tissue, or lymph nodes. T-VEC functions as a targeted therapy to eliminate melanoma cells and reduce skin and lymph node lesions. However, it has not been demonstrated that T-VEC can reduce metastatic melanoma (melanoma that has spread to the brain, bone, liver, lungs, or other organs) or increase overall survival. T-VEC is a local treatment, which implies that in order to cure melanoma cells, it is given directly to the lesions. T-VEC's precise mechanism of action within the immune system remains not entirely understood. But it's suspected that along with killing cells directly, the virus may also trigger an immune reaction that fights melanoma by releasing antigens released from tumor cells, which are chemicals that trigger an immune response, and GM-CSF, a molecule that boosts the immune system [211, 212].

A phase I study (NCT03458117) was conducted for the evaluation of effectiveness, safety, and tolerability after repeated T-VEC injections in patients with non-melanoma skin cancer [211].

A phase III study (NCT02965716) aims to assess the effectiveness of talimogene laherparepvec (T-VEC) in combination with pembrolizumab (MK-3475) in treating patients who have progressed on prior anti-PD-1 or anti-PD-L1 therapy, either alone or in combination with other drugs other than talimogene laherparepvec (T-VEC) [213].

c) Cancer Vaccines: Therapeutic vaccines like the MAGE-A3 vaccine target specific antigens expressed by cancer cells. Ongoing trials are exploring their effectiveness in stimulating immune responses against melanoma. mRNA-based vaccines can also elicit cellular and humoral immune reactions [214]. They are intended to stimulate the immune system to fight an existing disease rather than avoid sickness [215]. A vaccine that boosts the synthesis of a protein essential to the skin's antioxidant network, according to research from the Oregon State University College of Pharmacy, may help people strengthen their resistance to skin cancer [216]. Clinical trials for an mRNA vaccine have shown potential in the treatment of stage 3 and stage 4 melanoma. The incidence of death and recurrence from stage 3 and stage 4 melanoma was reported to be 44% lower when the Moderna vaccination was used with immunotherapy treatments as opposed to immunotherapy alone [217, 218]. An I.M. or I.D./S.C. injection of the immunotherapeutic MAGE-A3 was used in an open-label randomized single-institution pilot study (NCT01425749) to assess the safety and immunologic response. It was found that both immunization routes were well tolerated and free of grade 3 adverse treatment-related events [219]. Another study (NCT00002952) aims at determining the safety and maximum tolerable dose level of the MAGE-3 or Melan-A (human tumor antigen genes) peptide-pulsed autologous peripheral blood mononuclear cell and interleukin-12 vaccination should be determined. It also aimed to ascertain whether the procedure successfully immunizes the patient and to evaluate the tumor’s reaction to the vaccination [220].
d) BRAF/MEK Inhibitors with Immunotherapy: In melanoma cases with BRAF mutations, targeted therapy with BRAF and MEK inhibitors is combined with immunotherapy to maximize treatment response. Clinical trials are assessing the efficacy and safety of these combinations. When used before or simultaneously with immune checkpoint inhibitors, BRAF/MEK inhibitors at least momentarily change several immunosuppressive tumor microenvironment characteristics, which could possibly increase immunotherapy sensitivity [214]. The phase III trial IMspire150, which added atezolizumab (an anti-programmed death ligand 1 [PD-L1] drug) to cobimetinib (a MEK inhibitor) and vemurafenib (a BRAF inhibitor), produced encouraging findings for the first time in 2020. Despite the fact that response rates were comparable between the two arms, this immune/targeted triplet was observed to significantly lengthen the duration of response (DoR) and progression-free survival (PFS) [221].

A phase III trial (NCT01909453) compares the effectiveness and safety of LGX818 with MEK162 to vemurafenib and LGX818 monotherapy in patients with locally progressed, unresectable, or metastatic melanoma with the BRAF V600 mutation in two parts. There will be 900 patients randomized in total (64).

e) New Checkpoint Inhibitors: Beyond PD-1 and CTLA-4 inhibitors, researchers are developing novel checkpoint inhibitors targeting other immune checkpoints. These inhibitors are being studied in clinical trials for their potential in skin cancer treatment. It is imperative to research novel avenues targeting immune checkpoint receptor proteins because of the treatment resistance, poor response, or considerable increase in toxicity of antibody medicines targeting PD-1/PD-L1 or CTLA-4 that have previously been found [222, 223]. LAG-3, also known as CD223 or FDC protein, belongs to a new class of immunological checkpoint receptors [224]. According to studies, blocking or inhibiting LAG-3 can restore the cytotoxic activity of T cells, lessen the function of regulating T cells in suppressing immune responses, and increase the effect of T cells in destroying tumors. Beyond PD-1/PD-L1 and CTLA-4, LAG-3 has emerged as a novel tumor immunotherapy target as an indication of tumor prognosis [225, 226]. A phase II clinical trial (NCT03666325) aims to study the fighting of primary and secondary resistance with Immunotherapy after EGFR inhibitors in locally advanced or metastatic squamous cell carcinoma [227].
f) Personalized Immunotherapy: It is a type of highly individualized cancer therapy that uses the patient's immune system to battle tumor cells. For patients whose cancers do not respond to conventional therapy, the discovery of uncommon anti-tumor lymphocytes that can infiltrate and aid in the destruction of metastatic solid epithelial tumors could promote the development and efficacy of personalized cancer immunotherapies [228]. CAR-T cell therapy is being explored in certain skin cancers. Researchers are identifying specific antigens and designing CAR-T cells to target them, with ongoing studies to assess their effectiveness and safety.

A phase I trial (NCT03893019) using CD20 CAR-transduced T cells to treat melanoma will be the first one in Europe. The trial's justification is based on the discovery that CD20 is expressed by melanoma cancer cells and that killing CD20+ cells in preclinical models produces potent anticancer effects [229]. For the treatment of patients with stage IIIC or IV melanoma, this phase I trial (NCT04119024) investigates the adverse effects and optimal dose of modified immune cells (IL13Ralpha2 CAR T cells) [230].

g) Combination Therapies: Many ongoing trials focus on combining different immunotherapies, targeted therapies, or conventional treatments to enhance response rates, reduce side effects, and improve overall survival in skin cancer patients. The combination of anti-cancer medications improves efficacy in comparison to monotherapy because it targets important pathways in a manner that is typically additive or synergistic. This method may decrease the occurrence of drug resistance while also having therapeutic anti-cancer effects, such as lowering the growth of the tumour and metastatic potential, stopping mitotically active cells, lowering cancer stem cell populations, and causing apoptosis [231].

A Randomized, Double-blind, Placebo-controlled, Phase III Study (NCT02967692) is being conducted to evaluate the safety and efficacy of the combination of an anti-PD-1 antibody (Spartalizumab (PDR001)), a BRAF inhibitor (dabrafenib) and a MEK inhibitor (trametinib) in unresectable or metastatic BRAF V600 mutant melanoma (63). A clinical trial with identifier NCT02224781 titled “Dabrafenib and Trametinib Followed by Ipilimumab and Nivolumab or Ipilimumab and Nivolumab Followed by Dabrafenib and Trametinib in Treating Patients with Stage III-IV BRAFV600 Melanoma” aims to determine whether the following will significantly enhance 2-year overall survival (OS) in patients with stage III or stage IV BRAFV600 mutant melanoma that is not amenable to surgery (60).

### Targeted therapy

It is generally a dependent treatment and normally used for cancers that were present in closely associated lymph nodes and distant sites lymph nodes or any other organs located on distant areas, hence could be preferred for stages 1, 2, 3, and 4 of melanoma cancer. In this technique, drugs are used which were delivered on the target i.e., mutated genes and molecules present in melanoma cells stop the growth as well as affected the action of tumorous cells and also stop the proliferation of melanoma cells.

The medications used in targeted therapy arrest the proliferation of melanoma that constituted mutated serine/threonine-protein kinase B-Raf (BRAF), Mitogen-activated protein kinase (MEK), or tyrosine-protein kinase Kit (C-KIT) genes. Targeted therapy is only confined to these mutated genes, but not all melanoma cells do not possess these genes. Hence targeted therapy is not applicable to all types of cancer [232].

### Chemical peel

In a chemical peel, medical practitioners have to apply trichloroacetic acid (TCA) on the cancerous lesions directly, which results in the removal of the top layer of the skin. It is mainly used for precancerous skin lesions. The three types of chemical peels are; (i) Superficial peel – this kind of peel normally removes the top layer of the skin, (ii) Medium peel – this kind of peel is able to remove middle layers of skin, (iii) Deep peel – this kind of peels are able to penetrate the skin deeper and remove the deeper most layer of the skin.

Chemical peel treatment can be less costly and less complicated as compared to other treatments. Chemical peel treatment can make the skin appear red and the patch can stay for a month or a couple of months discoloration of the skin may be possible as the skin can become darker or lighter [233]. One review summarized the available data on the effects of chemical peels on UV- induced cancer of the skin. For the analysis, a total of 42 articles including in-vitro and in-vivo human and animal models were used. The information mostly concerned lab animals. The results indicate that chemical peeling may have clinical applications for the prevention of skin cancer in addition to its effectiveness in removing visible actinic keratoses. There is currently no proof that human skin exposure to chemical peels causes systemic harm [233].

### Radiotherapy

**Fig. 8:**
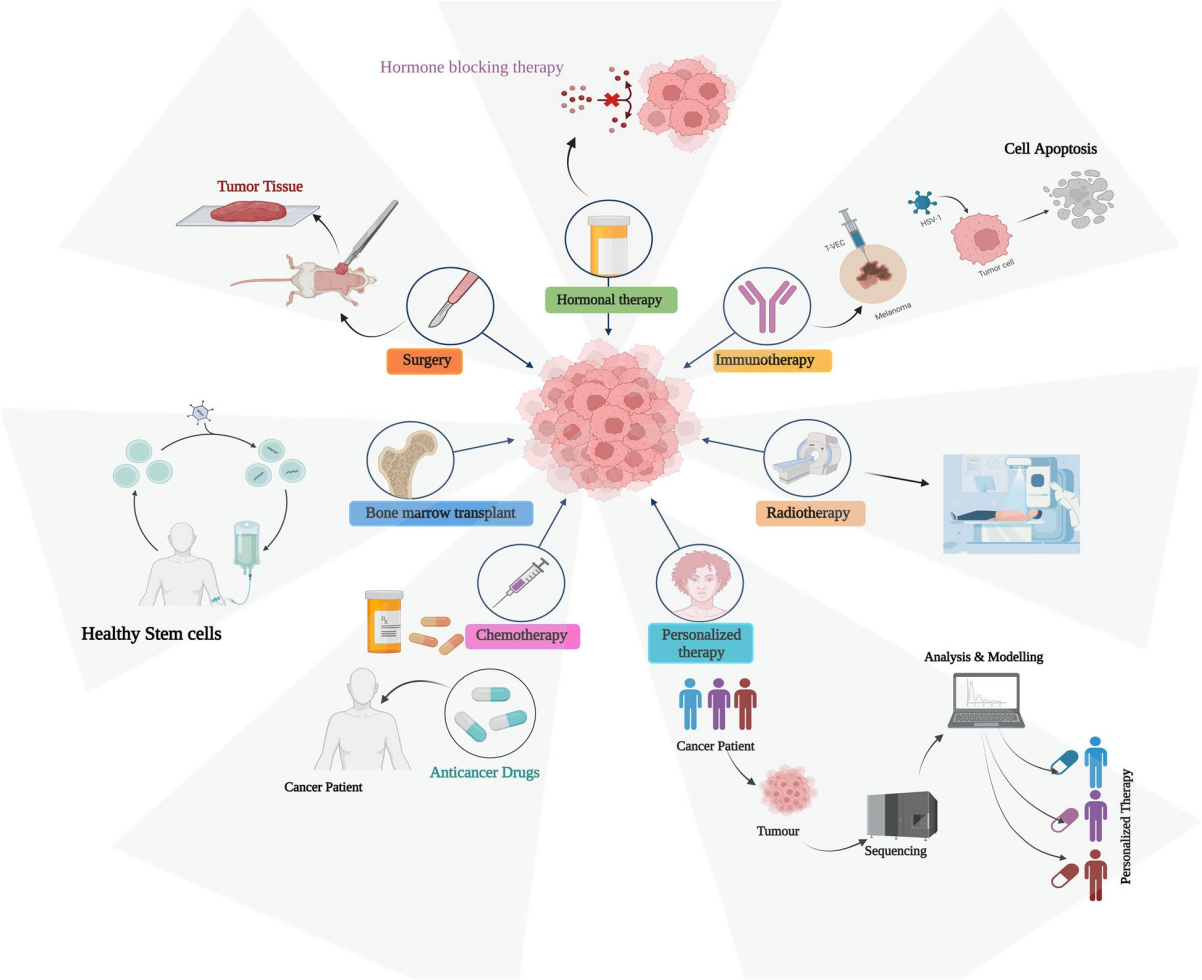
Representation of different approaches for the management of skin cancer

Radiotherapy is a successful and adaptable non-surgical (or medicinal) approach for tissue preservation. Radiotherapy is a fantastic alternative for people in whom excision may not be possible (medically or technically inoperable) or may be viewed as less optimal (e.g., cosmetic outcome). Adjuvant radiation after surgery can reduce the incidence of recurrence and related morbidity in individuals with unfavorable histology. When surgery is not a possibility, elderly and co-morbid individuals with low functional outcomes can profit from relatively brief hypo-fractionated radiotherapy [234]. In radiation therapy, the radiation is just focused on the outer surface of the skin on the tumorous area. The low energy x-ray (superficial radiation therapy) or electrons (electron beam radiation) is used for radiotherapy and it doesn’t penetrate deeper into the skin [235]. Radiotherapy is one of the prevailing kinds of treatment done by skin cancer patients. In the US approximately 50% patients of with skin cancer have an indication of performing radiotherapy at least once in the whole course of their disease. But most of the patients can’t receive the treatment due to a shortage of important resources including equipment as well as personnel for handling the instrumentation or the expertise persons who can do the whole treatment [236]. A phase II study was carried out for evaluating how older individuals with early-stage NMSC respond to accelerated radiation therapy (RT). Out of 31 study subjects, 30 showed complete responses. Concluding that > 90% of people had local control of disease which is considered to be a good or excellent result [237]. The results from a meta-analysis consisting of 344 articles published between the years 1985- 2016 were analyzed and it was concluded that for more than 80% of patients with cutaneous BCCs/SCCs, hypo fractionated RT has a positive cosmetic outcome [238]. Different approaches for tackling/ Management of skin cancer are well illustrated in Fig. 8.

## Dermal chemotherapy

Several treatment strategies have been designed to treat the never-ending rate of skin carcinoma. A void in the therapy regimen should be filled to facilitate cellular inhibition and apoptosis. The section below discusses the scope of the dermal therapeutic approach against skin cancer.

### Topical chemotherapy

Dermal drug delivery represents a broad term used to address the administration of dosage forms via transdermal (across) or topical (into) the skin [239]. In addition to oral, hypodermic, and intravenous injections, etc., the dermal drug administration technique has recently emerged as a crucial therapeutic strategy. The dermal approaches provide a number of advantages over competing ones [240]. In comparison to injections, it is painless, and because it uses needle-free technology, there is essentially no chance of disease transmission through the reuse of needles. This can help to reduce medical waste, which is a common source of medical malpractice, particularly in developing nations. Once more, the transcutaneous method can simply get around the drawbacks of the oral route. It can prevent gastrointestinal breakdown as well as first-pass or pre-systemic metabolism, in which the drug's dosage is drastically decreased before it even reaches systemic circulation. Consequently, it can increase the active pharmaceutical ingredients' (APIs) bioavailability. The non-invasive nature of the transdermal patches makes it a self-administered technology with better patient compliance, and they can deliver medications in a much more well-controlled manner over an extended period of time [240, 241]. Topical treatment may be more effective in some circumstances, especially in cases where the patient refuses surgical assistance or surgery is not an option. As a matter of fact, dermal or transdermal therapy is frequently advised to individuals who have several clinical and subclinical lesions spread across a significant anatomical area or field cancerization. In addition, topical therapy may result in lower patient toxicity than systemic therapy and higher medication concentrations at the tumor site [242].

The current dermal therapy for skin cancer includes 5- Fluorouracil, retinoids, Ingenol mebutate (IM), IMQ. Some of the phytopharmaceuticals used as dermal therapy include sinecatechins, Patidegib, BIL-010t, Gingerol, Betulinic acid, Epigallocatechin-3-gallate, Colchicine, resveratrol, quercetin, and curcumin.

#### 5- Fluorouracil (5-FU)

A 5% cream or solution of 5-FU is a common topical agent used against superficial Basal Cell Carcinoma (sBCC). 5-FU has been approved by the Food and Drug Administration (FDA) and the European Medicines Agency (EMA) as a therapeutic of sBCC along a dosage regimen of two times a day for two to four weeks. The pyrimidine analog 5-FU is an antineoplastic antimetabolite, and through 5,10-methylene tetrahydrofolate which is a co-factor, it shows binding to thymidylate synthase. As a result, thymidine synthesis is inhibited, DNA replication is compromised, and apoptosis is subsequently induced [243].

In a prospective, single-arm study, 5-FU cream (5%) was used on 29 patients with a total of 31 sBCC lesions on the trunk or limbs as confirmed by histology. The treatment was done two times a day for a period of 12 weeks. The histological analysis showed that 28 lesions (or 90%) out of the 31 were cured [242].

Patients with sBCC in a randomized controlled study were given 5% of 5-FU two times a day for 4 weeks, MAL-PDT twice a week apart, and 5% cream of imiquimod once a day for six weeks. Five- year follow- up was done on the patients. At 1 year the estimated success of treatment was 80.1% with 5% 5-FU, 72.8% for MAL-PDT, and, 83.4% on using imiquimod which has proved the superiority of imiquimod to MAL-PDT in treating sBCC while topical 5-FU was not inferior. Adnexal expansion and tumor thickness in sBCC did not seem to indicate treatment failure. The likelihood of being tumor-free five years after treatment with 5% 5-FU was 70.0%, with MAL-PDT was 62.7% and 80.5% for imiquimod. Thus, proving the superiority of 5% 5-FU over MAL-PDT and that of imiquimod over both 5-FU and MAL-PDT [167].

**Fig. 9:**
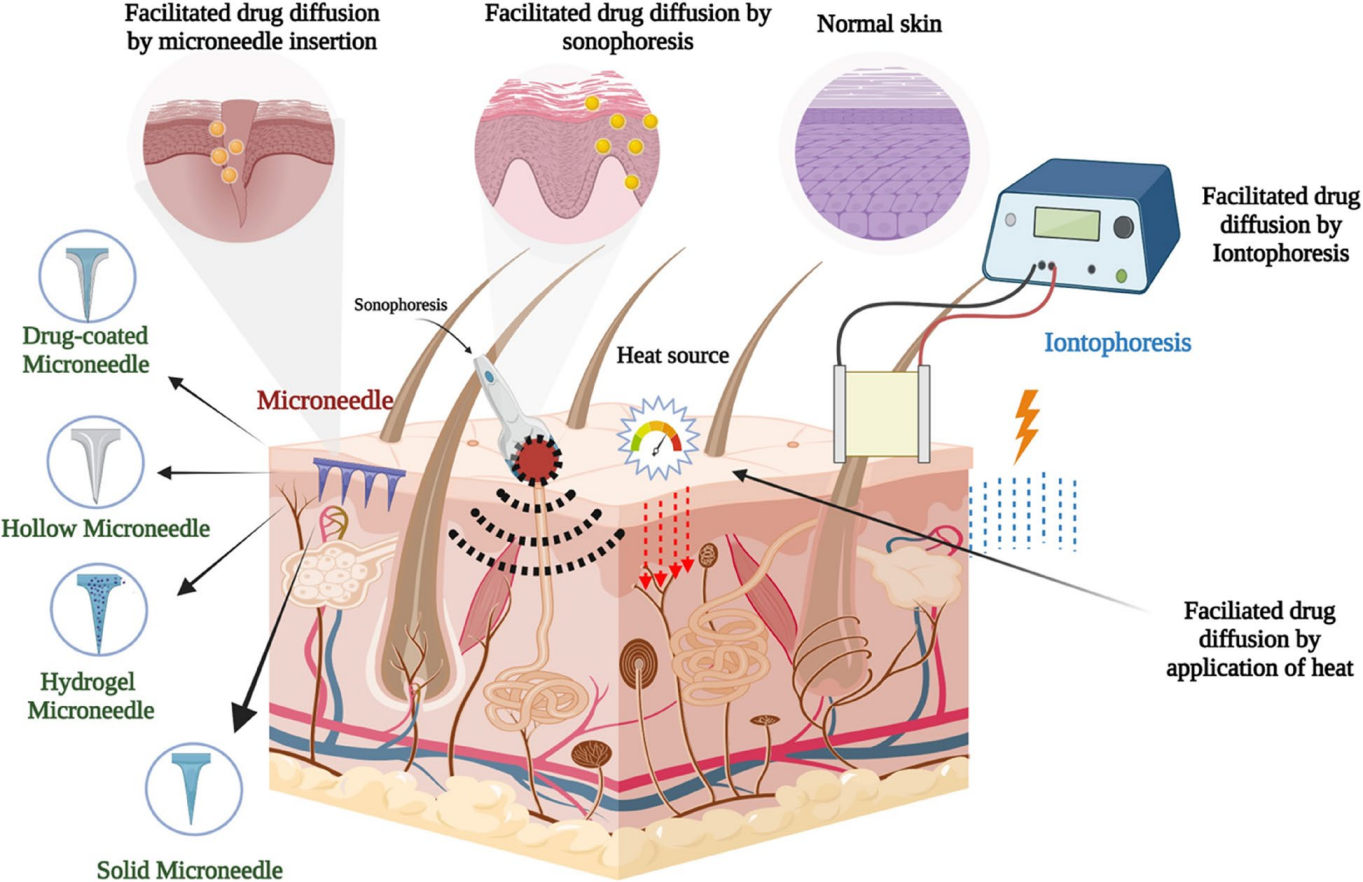
Physical methods employed for enhancing the penetration ability of the drug

The consideration of 5-FU as a treatment option for sBCC is possible, however, it should only be used on people who are unable to have surgery and have small lesions in low-risk sites. Additionally, long-term follow-up must be maintained. Sahu Prashant et. al. worked on the pH-responsive biodegradable chitosan nanogels incorporated with 0.1% and 0.2% 5-FU, and the developed formulation was applied topically on the Swiss albino male mice bearing skin cancer and compared with the control. The study showed promising results in terms of reducing tumor size and efficacy of the formulation. As shown in Fig. 9, the Group G1 (Control), G2 (Toxic group), G3 (treatment with conventional 5-FU formulation), G4 (treatment with 1% 5-FU loaded chitosan nanogel), and G5 (treatment with 2% 5-FU loaded chitosan nanogel). The decreasing order of efficacy found after conducting the study on tumor-bearing mice was G5 > G4 > G3 > G2. The graph represented in Fig. 9 also shows the tumor burden in G5 was minimum compared with the remaining groups [244].

### Topical immunotherapy

#### Imiquimod (IMQ)

IMQ is a synthetic compound of low- molecular weight. It belongs to the family of imidazoquinolinone. A high range of tumor necrosis factor-alpha (TNF-alpha), interferon-alpha (IFN-alpha), and other interleukins (IL-6, IL-8, IL-10, and others) are determined by IMQ's binding to Toll-like receptor (TLR-7) and -8. It is possible that IMQ could be utilized for individuals having skin cancers, particularly in smaller tumors at insubstantial- risk areas, those not convenient for alternate treatments since it can also cause cell apoptosis [245]. 5% cream of IMQ is used off- label for nBCC. However, it is an approved drug used against sBCC [242].

A randomized trial was carried out to assess the effectiveness of topical IMQ and ascorbic acid for treating BCC. 25 patients with BCC confirmed by 29 biopsies were randomly allocated to be given topical imiquimod, or topical ascorbic acid solution two times a day for 8 weeks. In the ascorbic acid group, 13/15 (86.7%) of the lesions had completely disappeared after 8 weeks, but with IMQ the lesions disappeared only in 8/14 (57.1%) cases. The superiority of topical ascorbic acid over topical imiquimod in the treatment of low-risk nodular and surficial lesions was found at 8 weeks and by 12 weeks it was found to be non-inferior. Additionally, in comparison ascorbic acid was linked to fewer side effects than IMQ. At the 30-month follow-up, persistent hypopigmentation was found in 70% of individuals belonging to the IMQ category, compared to 0% in the ascorbate category [246].

One of the studies concerning therapy with topical IMQ 5% has demonstrated a higher rate of success when compared to PDT and 5-FU. Although there does not appear to be a correlation between the thickness of the tumor and the rate of success for the three aforementioned treatments [245].

A systematic review was done by Huang and colleagues on the use of topical IMQ as a treatment option for nBCC including its safety and efficacy. The treatment regimen varied from once daily twice a week to two times daily 7 days a week. It was concluded that histological and clinical clearance rates for nBCC treated with imiquimod exceeded 70%, with a recurrence incidence of 1.80%. Although IMQ has lower clearance rates than surgery, it can be used to treat nBCC in patients who are unwilling or unable to undergo surgical intervention [247].

**Fig. 10:**
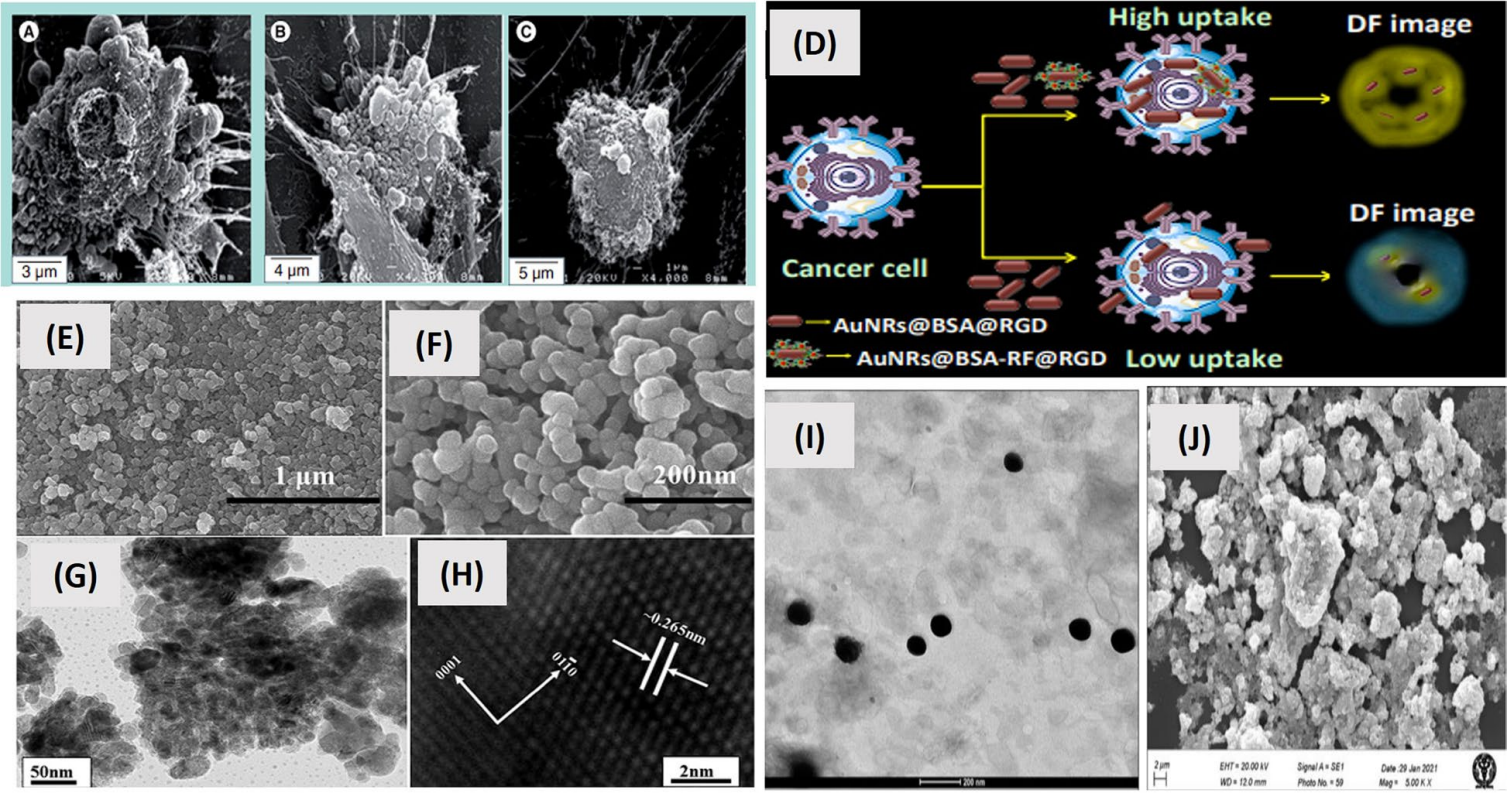
SEM image of Me300 melanoma cells in which **A**) represents cells not exposed to iron oxide nanoparticles while **B** and **C**) represent treatment groups exposed to amino ultra-small superparamagnetic iron oxide nanoparticles [250], (**D**) influence of @BSA-RF@RGD on AuNRs uptake [251], (**E**) FESEM and TEM images of ZnO nanoparticles in which Low magnification FESEM image, (**F**) high-magnification FESEM image, (**G**) Low magnification TEM image of ZnO, and (**H**) high-magnification TEM image of ZnO showing distance between lattice fringes around 0.265 nm [252], (**I** and **J**) TEM and FESEM image of cerium oxide nanoparticles respectively. Adapted with permission from [253]

According to case report data, IMQ may be utilized in some situations as a treatment prior to surgical excision of high-risk BCC or as an adjuvant therapy for partially removed tumors [248]. Topical IMQ has the benefits of being self-applied, being a non-scarring therapy, being less costly, and being less painful. Additionally, it can be utilized as a substitute for surgery for lesions that include sensitive regions or huge areas that are resistant to surgery [242]. Franca, et al. developed a polymeric nanoparticle containing Imiquimod and the efficacy of these formulations was compared with the marketed Imiquimod formulation, Placebo as well as Tumor control. The efficacy of each formulation was determined on the tumor-bearing rats based on the reduction of no. of papilloma in each group as shown in Fig. 10. The study results revealed that the Imiquimod loaded polymeric nanoparticles showed greater efficacy against tumors followed by the marketed Imiquimod formulation and placebo showing the enhanced effectiveness of the Nano formulation approach over the conventional formulation of Imiquimod [249].

### Other drug therapies

#### Retinoids

Retinoids were discovered in 1913 and are defined to be natural or synthetic derivatives of vitamin A. Basal cell carcinomas (BBCs) have responded well to treatment with retinoic acid derivatives. Retinoids exert their chemo-preventive effects by arresting the growth of tumor cells, apoptosis, altering the immune response or keratinocyte differentiation, or by a combination of these actions. Retinoids interact intracellularly with cytosolic proteins as well as two families of retinoic acid receptors (RARs), nuclear receptors, and retinoid X receptors (RXRs). This activates downstream targets’ transcription that contains retinoic X response elements (RXREs) or retinoic acid (RAREs). Skin malignancies have been treated with isotretinoin (also known as 13-cis-retinoic acid), tretinoin (also known as all-trans retinoic acid), tazarotene, and adapalene. However, they are all in use experimentally off- label, as none of them have been approved. In particular, a clinical investigation investigated how to treat sBCC or nBCC, for this once-daily application of 0.1% tazarotene gel for up to 8 months was carried out. It was observed that 5 of 17 nBCC cases and 11 of 13 sBCC cases showed elimination of lesions. The development of alternate forms of therapy to BCC lowered the use of topical retinoids.

A randomized, open, controlled trial comparing tretinoin 0.05% cream with low-dose oral isotretinoin was conducted for the purpose of treating field cancerization. The results indicated that retinoids are efficient and secure for field cancerization because there was no difference in the outcomes between the two treatments. Retinoids are a suitable option to intercalate with traditional field cancerization therapies (IM and IMQ) which are used for short time periods. However, retinoids can be taken constantly [254].

Tazarotene, a retinoic acid derivative was successfully used in one of the clinical study to treat BBCs. Another retinoid called Fenretinide is thought to be among the most optimistic. Skin tumor cells as well as other cancer cells are cytotoxic to the substance. N-(4-hydroxyphenyl) retinamide (4-HPR) impacts tumors by generating ROS, boosting the formation of dihydroceramide, encouraging angiogenesis inhibition, and enhancing Natural killer cells (NK) cell activity [255].

#### Ingenol mebutate (IM)

IM is one of the extracted substances from Euphorbia peplus plant sap. IM is marketed by LEO Pharma with the brand name Picato and is an activator of protein kinase C. IM causes cellular cytotoxicity and prevents recurrence by promoting the development of anti-tumor antibodies and proinflammatory cytokines [245]. It is approved for treatment against actinic keratoses but is used off-label for SCC and BCC [256].

Treatment with IM gel has been demonstrated to be an effective therapy for both non-pigmented and pigmented superficial BCC, with no significant adverse events. Histology and dermoscopy techniques were used to observe these outcomes. Only the highest concentration (0.05%) administered over a period of days was significantly superior to the vehicle when used to treat superficial BCC, according to a phase II trial. More studies are required as currently indicated therapy for BCC is off-label [257].

According to Erlendsson et al., repeated IM field-directed treatments prevent hairless mice from developing UV-related SCC. Additionally, the scientists discovered a correlation between higher local skin responses, such as erythema, flaking, crusting, vesiculation, swelling, and ulceration, and better clinical results. At the moment, it is used off-label to treat SCC [258].

M. R. Fuente and colleagues performed a study concerning topical IM 0.05% gel in BCC, triggering the inflammatory response and its characterization. 16 patients with distinct BCC subtypes were prospectively assessed and treated with IM gel 0.05% once daily for two days in a row under occlusion. Patients were randomly assigned into two arms, patients in one of the arms received biopsies between the 3^rd^ and 10^th^ day following the start of the treatment (referred to as the "early immune response") and another arm received biopsies at the thirty-day mark (referred to as the "late immune response"). Complete remission in 10 BCCs was found after 2 years of follow- up. It was concluded that IM gel causes a brief immuno-inflammatory response as well as a significant necrotic reaction [259].

Another study described the example of the 100-year-old woman with a massive nBCC (nodular BCC) of the face. IM 0.015% gel was used for three days in a row, once a day. Seven cycles of this dosage regimen were administered over the course of 11 months, and the tumor was completely cured. Mild localized cutaneous responses that were well tolerated were noted. IM gel may be used to treat some cases of nBCC; however, the ideal medication concentration and dosage schedule have not yet been established. Below listed Table 2 represents the FDA-approved drugs for the management of skin cancer.

**Table 2:** List of drugs approved by FDA for skin cancer treatment

**Generic name**	**Trade Name**	**Dosage form**	**Mechanism of action**	**Indication**	**Side effects**
**Imiquimod**	Aldara/ Zyclara	Cream/ ointment (topical)	Immune response modifier also treats genital and anal warts by enhancing the activity of the body’s immune system	Use to treat superficial basal cell carcinoma and actinic keratosis	Redness, itching, headache, diarrhea, Back pain, tiredness, etc
**Vismodegib**	Erivedge	Capsule (oral)	Works as hedgehog pathway inhibitor	Generally used to treat basal cell carcinoma and malignant melanoma, can’t be consumed by pregnant women	Muscle spasms, tiredness, joint pain, hair loss, weight loss, etc
**Sonidegib**	Odomzo	Capsule (oral)	Work as hedgehog pathway inhibitor	Commonly used to treat basal cell carcinoma and also that kind of skin cancer which are reoccur after the surgical or radial treatment and can’t be consumed by pregnant women	Diarrhea, abdominal pain, loss of appetite, nausea, vomiting, anemia, hair loss, etc
**5-fluorouracil**	Carac/ Efudex/ Fluoroplex/Tolac	Cream/ointment(topical)	It is a kind of antimetabolite that acts by killing fast-growing abnormal cells by inhibiting DNA formation	Generally used to treat actinic keratosis, and solar keratosis which were caused due to overexposure of UV rays by few years	Burning, crusting, redness, pain, discoloration, etc

## Physico-chemical combination therapies

Cancer has many potential ways to worsen since it can develop as a result of mutations in several pathways that are present in our bodies. Due to this characteristic, treating cancer patients with monotherapy alone becomes challenging and runs the risk of not completely curing the disease. Therefore, the need was felt for combinatorial therapy, which includes the simultaneous use of two or more methods or drugs for the treatment of a disease. A combination of two or more drugs or methods would target multiple pathways simultaneously and would help in the better treatment of cancer. This has also yielded a higher success rate as compared to monotherapy. Combinatorial treatment involves the complimentary pairing of two separate therapies, such as the pairing of radiotherapy with immunotherapy or the pairing of medications that can target several cancer-formation pathways. Along with complimenting each other, the combination also leads to reduced drug resistance which is generally seen with monotherapy [260, 261].

Multidisciplinary involvement in the treatment of local, regional, or distant metastatic cSCC is necessary. When lymph nodes are involved, NCCN (National Comprehensive Cancer Network) advises excision lymphadenectomy or parotidectomy. Depending on the results of the margin assessment or if extracapsular lymph node expansion is observed, adjuvant chemoradiation may be considered [262].

In one of the studies (NCT03057613) concerning SCC, Pembrolizumab was added along with postoperative radiotherapy. The study showed 94.4% progression-free survival and no dose-related toxicity. Some minor toxicities like anemia, rash, fever, edema, etc., were found in 5.56% of the participants [263]. A phase 3 trial (NCT03833167) is underway, recruiting participants with CSCC (advanced but not metastasized), using pembrolizumab against placebo following radiation and surgery (MK-3475–630/KEYNOTE-630). The findings of a phase II trial (NCT01979211) named “Post-operative Radiation with Cetuximab for Locally Advanced Cutaneous Squamous Cell Carcinoma of the Head and Neck” showed 91.1% local–regional control, 70.8% disease-free survival, 56.1% overall survival and a 37.5% of participants faced all-cause mortality [264].

Singh et. al. formulated liposomal gold nanoparticles encapsulating curcumin and used it as an adjuvant with photothermal treatment. When compared to free curcumin, Curcumin encapsulated gold nanoparticles considerably improved uptake. Following laser irradiation, the group treated with curcumin gold nanoparticles showed enhanced cytotoxicity to cancer cells because of the presence of curcumin. The study suggests that for the treatment of melanoma, curcumin can be used as an adjuvant along with PTT [265]. Moghaddam et. al. formulated PEG- curcumin- gold nanoparticle (PEG- cur- Au NP) and used it as a near-infrared photothermal agent, it was found that when this is used in combination with 808-nm diode laser irradiation, a sizeable reduction in the volume of the tumor was seen *in- vivo*, with no adverse effects on the animal's well-being [266].

Cheng et. al. designed a Titanium-dioxide-nanoparticle–gold-nanocluster–graphene (TAG) heterogeneous for efficient utilization of sunlight which resembles melanoma skin cancer PDT, which would decrease or make the pain less severe as compared to PDT. TAG nanocomposite when used on mouse B16F1 melanoma cells has shown significant toxicological reactions when exposed to simulated sunlight. These toxicological reactions include glutathione depletion, intracellular reactive oxygen species generation, mitochondrial dysfunctions, and expression of heme oxygenase-1. Furthermore, in mice bearing B16F1 tumor xenografts, intratumoral or intravenous treatment with TAG nanocomposites can effectively suppress cancer growth and result in severe clinical tumor tissue alterations [267].

Following illumination (720 J/cm2; 650 nm filter) using or excluding a gel of kojic acid, a transethosome encapsulating ferrous chlorophyllin (Fe-CHL), a lipid-based NP, caused a progressive reduction in the size of the tumor in a melanoma mouse model, which totally regressed under one month [268]. Chlorin-e6 also called meso-tetraphenyl morphine – Mn (III) chloride, which is a generation- three photosensitizer, showed effective internalization within malignant melanoma cells after being encapsulated in liquid crystalline nano dispersions (LCNs), and they showed notable photodynamic effects after being exposed to radiation as compared to free photosensitizer (PS) [269]. Following exposure to radiation (0.5, 1.0, and 2.0 J/cm2; 670 nm filter), aluminum chloride phthalocyanine loaded SLN showed a decrease of 3.2-fold in the viability of the cell line (B16F10) [270].

Photodynamic therapy (PDT) in combination with photothermal therapy (PTT) is now showing promise in cancer therapy, overcoming PDT's intrinsic limitations. Complete cell/tumor eradication has been seen in one as well as other *in- vivo* along with *in- vitro*, recommended that the combined PDT/PPTT action caused by AuNRs/MoS2-ICG is significantly more effective than either one of synergistic PPTT or PDT alone. Remarkably, the two plasmonic nano agents producing synergistic PPTT have higher antitumor activity *in- vivo* as compared to PDT or PTT alone. When used in conjunction, the simultaneous PDT/synergistic PPTT approach efficiently shortens the duration of the therapy, has a high therapeutic index, and provides a safe treatment alternative as a cancer therapeutic [271].

Gefitinib was inspected in phase II single-arm analysis (NCT00126555) in persons suffering from CSCC who were contenders for curative therapy, such as radiation or surgery. 13 patients (35%) had a stable illness at 8 weeks, with an overall response rate of 16%. The median response and progression-free survival times, respectively, were 31.4 months and 3.8 months. A modest amount of efficacy was seen by gefitinib in incurable CSCC, and its adverse event profile was favorable [123].

In a multicenter Randomized Control Trial, the use of diclofenac gel in addition to cryosurgery showed to be superior to cryosurgery alone. Photodynamic therapy has also demonstrated efficacy against nonmelanoma skin cancer as well as premalignant lesions, either alone or in conjunction with topical immunomodulators [204]. Wang et al. reported that light-responsive Doxorubicin (DOX) -loaded nanocapsules showed considerable anticancer action in vivo as well as in vitro. The study found that succeeding with receiving pulsed microwave radiation exposure, the nanocapsules consumed energy to create a significant thermoacoustic shockwave that concurrently split fragments into ammonia as well as carbon dioxide, resulting in the creating the partial vacuum condition and eventually causing cellular destruction. The thermoacoustic shockwave as well as gas bursting emanate in the production of a significant number of destructive cells. Then, to begin cell death, DOX was released into the nucleus from the cytosol [272]. In one study, imiquimod (R837) loaded gold nanorods (GNR) coated with cetyltrimethylammonium bromide (CTAB) and adorned using polyethylene glycol (PEG), bovine serum albumin (BSA) was used as an immunoadjuvant. In mice with metastatic melanoma, the produced nano complexes subjected to near-infrared (NIR) radiation can provoke potent immune responses and efficiently destroy tumors. Melanoma and other malignant tumors may potentially be successfully treated with the nano complex-based PTT in conjunction herewith immunotherapy [273].

The delivery of medications to an area of interest using iontophoretic devices has been modified for the treatment of cancer. Iontophoresis is a drug delivery technique that involves applying a small electric current all around a drug reservoir to enhance drug transfer into the surrounding tissue [274]. Jose et al. studied the treatment of skin cancer using topical iontophoretic liposomal co-delivery of STAT3 siRNA and curcumin. Cationic liposomes complexed with STAT3 siRNA were used to encapsulate the curcumin. In an excised porcine skin model, iontophoresis applied topically was used to examine the penetration of nano complexes through the skin. The outcomes demonstrated that cells preferentially absorbed the curcumin-loaded liposome-siRNA combination through clathrin-mediated endocytosis. In comparison to free STAT3 siRNA and neat curcumin treatment, liposomal delivery of curcumin along with STAT3 siRNA significantly increased inhibition of the growth of cancer cells and apoptotic activities. Additionally, topical iontophoresis application improved the skin's ability to allow viable epidermis to penetrate nano complex [274–276]. Another study showed an enhanced skin penetration of the drug by iontophoresis. An EGFR-targeted 5-FU-loaded immunoliposome was developed. The study exhibited that penetration through viable epidermis doubled for 5-FU by iontophoresis of immunoliposomes in comparison to identical therapy using control liposomes. The therapeutic effectiveness was confirmed by the results in a mouse skin cancer model, which showed a decrease in cellular proliferation and tumor volume [277].

Targeted modulation and site-specific administration of therapeutic drugs are made possible by the spatiotemporally programmable application of ultrasound, either with or without ultrasound contrast agents [278]. Through the activation of sonosensitizers by low-intensity ultrasound, sonodynamic therapy (SDT) has turned out to be a viable treatment alternative for the management of cancer. Docetaxel is loaded onto a redox/enzyme/ultrasound responsive chondroitin sulfate-chlorin e6-lipoic acid nanoplatforms, integrating chemotherapy and SDT, for the prevention of growth of melanoma as well as metastasis. Chemo SDT along with the prevention of tumor development and metastasis by lowering the expression of metastatic proteins, but also triggered an immune response by releasing antigens related to the tumor. The nanoplatforms demonstrated good blood compatibility and *in-vivo* stability, proving them to be efficacious and safe in drug delivery [279].

**Fig. 11:**
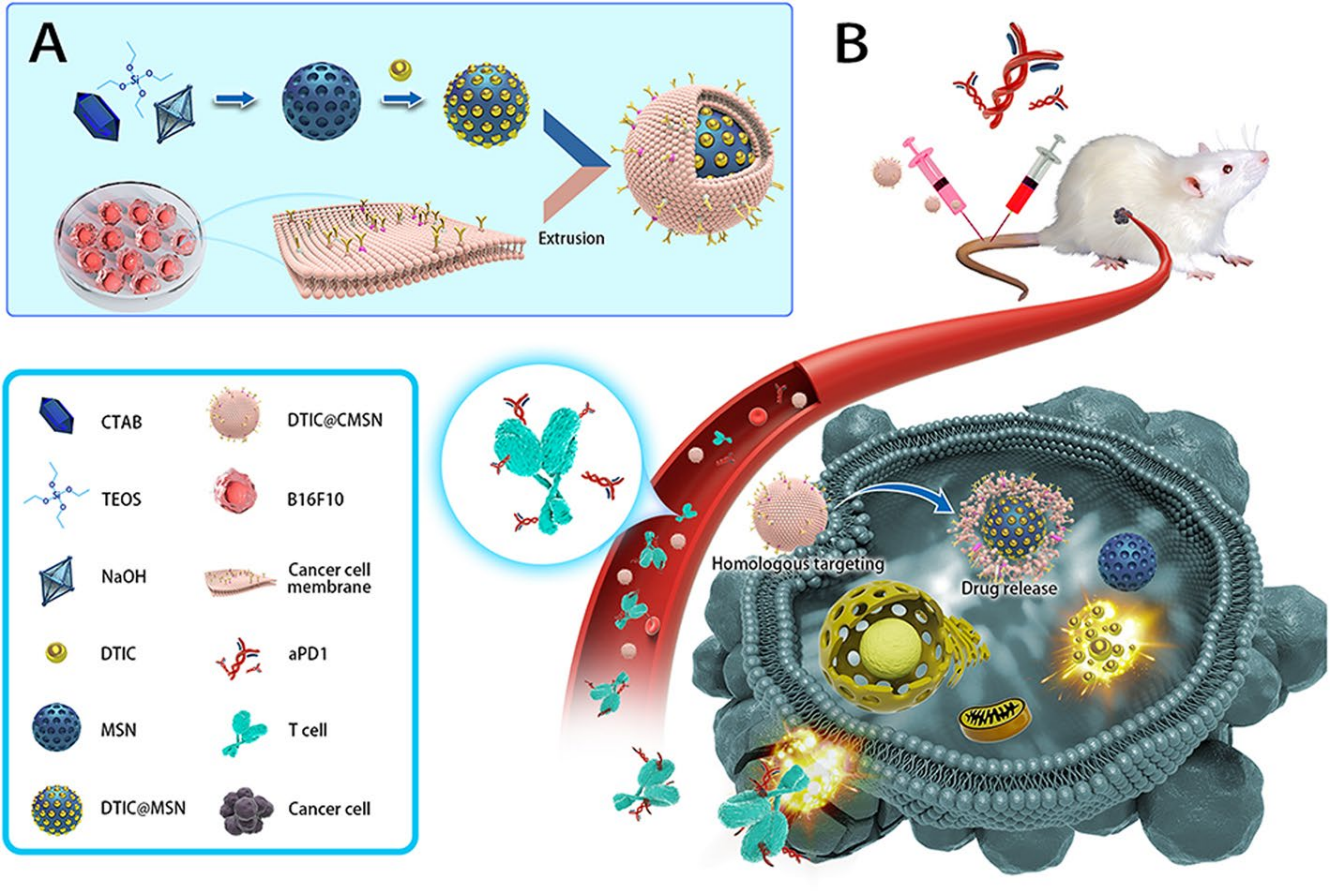
**A**) Process of fabrication of cancer cell membrane camouflaged Dacarbazine loaded porous silica nanoparticles and **B**) Induction of antitumor immune response by DTIC@CMSN combined with aPD1. Adapted with permission from [281]

Curcumin and Topotecan were evolved by Prasad and Banerjee co-encapsulating nanocapsules and microbubble nanoconjugates for spatiotemporal delivery in melanoma. When ultrasound was used along with this developed formulation the effect in terms of reduced tumor growth was found to be 3.5 and 14.8 times more as compared to when ultrasound was not used and when only a physical mixture of drugs was used, respectively [280]. Below listed Table 3 elaborates on the list of ongoing clinical trials on physicochemical combination therapies for the management of skin cancer. Figure 11 represents different physical methods of penetration enhancement for the drug.

**Table 3:** Elaborates the list of ongoing clinical trials on physicochemical combination therapies for the management of skin cancer

**NCT Number**	**Study Title**	**Phase**	**Completion of Study**	**Current state**
**NCT04977453**	GI-101 as a Single Agent or in Combination with Pembrolizumab, Lenvatinib or Local Radiotherapy in Advanced Solid Tumors	Phase 1 Phase 2	September 2024	Recruiting
**NCT05498805**	PD-1 Inhibitors with or Without Radiation in Advanced Melanoma	Phase 2	August 15, 2025	Not yet recruiting
**NCT03969004**	Study of Adjuvant Cemiplimab Versus Placebo After Surgery and Radiation Therapy in Patients with High-Risk Cutaneous Squamous Cell Carcinoma	Phase 3	February 28, 2027	Recruiting
**NCT04879654**	Toripalimab Combined with Radiotherapy and Chemotherapy in the Treatment of SNMM After Endoscopic Surgery	Phase 2	May 1, 2026	Recruiting
**NCT01319565**	Prospective Randomized Study of Cell Transfer Therapy for Metastatic Melanoma Using Tumor Infiltrating Lymphocytes Plus IL-2 Following Non-Myeloablative Lymphocyte Depleting Chemo Regimen Alone or in Conjunction With 12 Gy Total Body Irradiation (TBI…	Phase 2	June 1, 2025	Active, not recruiting
**NCT02806258**	Comparing Sequential Neoadjuvant Treatment Including Chemotherapy and Accelerated Radiation Focused to the Tumor Bed vs Neoadjuvant Chemotherapy Alone	Phase 1 Phase 2	December 2025	Recruiting
**NCT03025724**	Photodynamic Therapy for Treatment of Cutaneous Squamous Cell Carcinoma in Situ	N/A	January 2020	Unknown
**NCT05359419**	Comparison of Two Modes of Photodynamic Therapy for the Treatment of Actinic Keratosis on the Upper Extremities	Phase 4	December 30, 2023	Not yet recruiting
**NCT03110159**	DUSA: Cyclic PDT for the Prevention of AK & NMSC in Solid Organ Transplant Recipients	Phase 1 Phase 2	January 2022	Recruiting

## Drug combination therapy

A combination of high-dose Ipilimumab along with high-dose interleukin-2 (IL-2) has shown an enhanced response rate, survival without cancer progression, or a larger percentage of complete responses of all desirable outcomes. Interferon alfa because of its significant immunomodulatory effects has been FDA-approved for high-risk melanoma treatment as an adjuvant [282].

In 2018 Bocanegra and colleagues used iron oxide magnetic nanoparticles (NPs) doped with zinc for the delivery of peptide antigens in combination with TLR agonists. On aggressive B16F10 melanoma cells, the vaccinations demonstrated greater effectiveness. Additionally, magnetic resonance and nuclear imaging allowed the researchers to follow the vaccine's progress from the injection site to the lymph nodes and tumor [283].

Faster responses and fewer adverse effects have been obtained when 5-FU and imiquimod are combined together for the treatment of skin cancer [204].

A 38.8% Objective Response Rate (ORR) was achieved with ipilimumab and T-VEC when used in combination. However, when ipilimumab is used as a single drug it only achieved ORR of 18.0%, as per the findings of a phase II randomized trial [284]. In phase II, an open-label, randomized trial concerning the safety and efficacy of Coxsackievirus A21 (CVA21) in combinatorial ipilimumab, ORR of 50% was found in participants with melanoma which was higher than the average rate of either drug alone [285].

Toll-like receptor-9 (TLR9), which is a target produced from plasmacytoid dendritic cell (pDC) precursors, has lately acquired popularity in melanoma clinical trials. The intralesionally given, CMP-001 is undergoing clinical trials as monotherapies and in various combinations with systemic immunotherapies. Preliminary data from the research, which is still ongoing, suggests that TLR9 agonists may be utilized in combination therapies to counteract immune depletion and limit metastasis by controlling the chronic inflammation brought on by systemic immunotherapies [286].

A 3^rd^ phase trial was directed on double-blind, randomized, placebo-controlled patients in 112 organizations in around 20 countries for cobimetinib, vemurafenib, and atezolizumab for advanced BRAF ^V600^ mutation-positive unresectable melanoma as first-line treatment. After 18.9 months, a median follow-up showed that in comparison to control, atezolizumab had remarkably prolonged progression-free survival [221].

In an early phase study, the combination of trametinib and atezolizumab against BRAF-wild-type melanoma showed promising outcomes in patients. When compared to monotherapy, the phase III study, which was completed in 2021, did not show any promising results [287, 288].

Durable clinical effect was previously seen with nivolumab with ipilimumab in contrast to monotherapy in the phase III CheckMate 067 study. In trial participants with advanced melanoma, the combination of nivolumab and ipilimumab demonstrated sustained, better clinical outcomes in comparison to monotherapy [289].

A phase 3 study was done for safety analysis and long-term survival using a combination of dabrafenib along with trametinib in the person suffering from metastatic melanoma with BRAF V600E/K- mutation. The finding of the study support long-term first-line treatment with this combination and showed the possibility of survival for a duration of 3 years [290].

An observational, retrospective, cross-sectional, multicenter study was conducted comprising forty-one patients having advanced melanoma who began receiving vemurafenib/cobimetinib combination therapy and who also had the BRAFV600 mutation. Twelve patients (29.3%) had a complete response to the combined therapy, whereas 19 (46.3%) had a partial response and five (12.2%) had progressive disease and 5 had stable disease. A total of 12 patients (29.3%) were thought to have received a durable response, while 29 patients (70.7%) received a non-durable response. One-third of the participants experienced a stable complete response as a result of using cobimetinib and vemurafenib in combination, which has a significant influence on long-term survival [291].

Petrilli et al. reported that Combining 5-FU topical therapy with cetuximab, an IgG1 recombinant human/mouse chimeric antibody that prevents EGFR activation and phosphorylation brought on by other ligands, can increase the effectiveness of 5-FU topical therapy against SCC. Cetuximab demonstrated a strong cytotoxic activity on A431 cells after 120 h of incubation, and synergism was shown by immunoliposomes containing cetuximab and 5-FU [277].

Due to encouraging results, a recent phase 3 trial for patients with V600E-variant melanoma was terminated early. One of the studies comparing vemurafenib monotherapy and a combination of dabrafenib and trametinib showed a greater 3-year overall rate of survival (11% for monotherapy while 25% for combination). Additionally, patients taking dabrafenib and trametinib together had a lower risk of cutaneous squamous cell carcinoma (Dean, 2017). For patients lying in stage III resected BRAF-mutant melanoma, dabrafenib combined with trametinib has been approved as adjuvant therapy [292].

Dickinson et al. reported that skin tumors prone skin, on treatment with a combination of rapamycin and PHT-427 together produced a considerable decline in tumor multiplicity in comparison to vehicle controls. For people with a high risk of developing cutaneous SCC, a combination of topical Akt inhibitors (PHT-427) and mTOR inhibitors (rapamycin) may be an effective chemoprevention strategy [293].

A phase 2 trial (NCT02955290) is being done with a combination of nivolumab and pembrolizumab to test the overall survival along with progression-free survival in patients suffering from squamous cell carcinoma (SCC). Although they have not yet received FDA approval for SCC treatment, pembrolizumab and nivolumab are FDA-approved for treating metastatic melanoma or unresectable melanoma [294]. Table 4 gives a brief about list of ongoing clinical trials comprising drug combinations for skin cancer management.

**Table 4:** Emphasis on ongoing clinical trials comprising drug combinations for skin cancer management

**NCT Number**	**Study Title**	**Phase**	**Completion of Study**	**Current state**
**NCT03524326**	5-Fluorouracil and Calcipotriene for Treatment of Low-Grade Skin Cancer	Phase 2 Phase 3	June 2025	Active, not recruiting
**NCT03524326**	Testing Lenvatinib and Cetuximab in Patients with Advanced Head and Neck Squamous Cell Carcinoma and Cutaneous Squamous Cell Carcinoma	Phase 1	April 2023	Active, not recruiting
**NCT03944941**	Avelumab With or Without Cetuximab in Treating Patients with Advanced Skin Squamous Cell Cancer	Phase 2	December 2023	Recruiting
**NCT02324608**	Cetuximab Before Surgery in Treating Patients with Aggressive Locally Advanced Skin Cancer	Not applicable	December 2022	Active, not recruiting
**NCT03082534**	Pembrolizumab Combined With Cetuximab for Treatment of Recurrent/Metastatic Head & Neck Squamous Cell Carcinoma	Phase 2	May 2023	Active, not recruiting
**NCT03108131**	Cobimetinib and Atezolizumab in Treating Participants with Advanced or Refractory Rare Tumors	Phase 2	July 30, 2022	No result posted
**NCT04007744**	Sonidegib and Pembrolizumab in Treating Patients with Advanced Solid Tumors	Phase 1	July 31, 2024	Recruiting
**NCT02955290**	CIMAvax Vaccine, Nivolumab, and Pembrolizumab in Treating Patients with Advanced Non-small Cell Lung Cancer or Squamous Head and Neck Cancer	Phase 1 Phase 2	December 9, 2023	Recruiting
**NCT04234113**	Study of SO-C101 and SO-C101 in Combination with Pembro in Adult Patients with Advanced/Metastatic Solid Tumors	Phase 1	December 2023	Recruiting
**NCT03590054**	Abexinostat in Combination with Pembrolizumab in Patients with Advanced Solid Tumor Malignancies	Phase 1	November 30, 2023	Active, not recruiting
**NCT03684785**	Intratumoral Cavrotolimod Combined with Pembrolizumab or Cemiplimab in Patients with Merkel Cell Carcinoma, Cutaneous Squamous Cell Carcinoma, or Other Advanced Solid Tumors	Phase 1 Phase 2	March 30, 2022	Terminated due to administrative reasons
**NCT03082534**	Pembrolizumab Combined With Cetuximab for Treatment of Recurrent/Metastatic Head & Neck Squamous Cell Carcinoma	Phase 2	May 2023	Active, not recruiting
**NCT03816332**	Tacrolimus, Nivolumab, and Ipilimumab in Treating Kidney Transplant Recipients with Selected Unresectable or Metastatic Cancers	Phase 1	May 30, 2023	Suspended
**NCT02955290**	CIMAvax Vaccine, Nivolumab, and Pembrolizumab in Treating Patients with Advanced Non-small Cell Lung Cancer or Squamous Head and Neck Cancer	Phase 1 Phase 2	December 9, 2023	Recruiting
**NCT03767348**	Study of RP1 Monotherapy and RP1 in Combination with Nivolumab	Phase 2	November 2024	Recruiting
**NCT04050436**	Study Evaluating Cemiplimab Alone and Combined with RP1 in Treating Advanced Squamous Skin Cancer	Phase 2	March 2025	Recruiting
**NCT05544929**	A Study of Safety and Efficacy of KFA115 Alone and KFA115 in Combination with Tislelizumab in Patients with Select Advanced Cancers	Phase 1	September 15, 2025	Not yet recruiting
**NCT05220748**	RM-1995 Photoimmunotherapy, as Monotherapy or Combined with Pembrolizumab, in Patients with Advanced CuSCC and HNSCC	Phase 1	September 2024	Recruiting
**NCT02419495**	Selinexor With Multiple Standard Chemotherapy or Immunotherapy Regimens in Treating Patients with Advanced Malignancies	Phase 1	December 31, 2022	Recruiting
**NCT02304458**	Nivolumab With or Without Ipilimumab in Treating Younger Patients with Recurrent or Refractory Solid Tumors or Sarcomas	Phase 1 Phase 2	October 5, 2022	Active, not recruiting
**NCT02159066**	LGX818 and MEK162 in Combination with a Third Agent (BKM120, LEE011, BGJ398 or INC280) in Advanced BRAF Melanoma	Phase 2	January 17, 2023	Active, not recruiting
**NCT04835805**	A Study to Evaluate the Safety and Activity of Belvarafenib as a Single Agent and in Combination with Either Cobimetinib or Cobimetinib Plus Atezolizumab in Patients With NRAS-mutant Advanced Melanoma	Phase 1	November 11, 2024	Recruiting
**NCT04526899**	Trial With BNT111 and Cemiplimab in Combination or as Single Agents in Patients with Anti-PD-1-refractory/Relapsed, Unresectable Stage III or IV Melanoma	Phase 2	June 2025	Recruiting
**NCT04305041**	Substudy 02A: Safety and Efficacy of Pembrolizumab in Combination with Investigational Agents in Participants with Programmed Cell-death 1 (PD-1) Refractory Melanoma (MK-3475-02A/KEYMAKER-U02)	Phase 1 Phase 2	April 3, 2030	Recruiting
**NCT04305054**	Substudy 02B: Safety and Efficacy of Pembrolizumab in Combination with Investigational Agents or Pembrolizumab Alone in Participants with First Line (1L) Advanced Melanoma (MK-3475-02B/KEYMAKER-U02)	Phase 1 Phase 2	April 3, 2030	Recruiting
**NCT04303169**	Substudy 02C: Safety and Efficacy of Pembrolizumab in Combination with Investigational Agents or Pembrolizumab Alone in Participants with Stage III Melanoma Who Are Candidates for Neoadjuvant Therapy (MK-3475-02C/KEYMAKER-U02)	Phase 1 Phase 2	April 3, 2030	Recruiting
**NCT04700072**	Substudy 02D: Safety and Efficacy of Pembrolizumab in Combination with Investigational Agents or Pembrolizumab Alone in Participants with Melanoma Brain Metastasis (MK-3475-02D/KEYMAKER-U02)	Phase 1 Phase 2	April 3, 2030	Recruiting
**NCT04370587**	A Clinical Study of Intratumoral MVR-T3011 (T3011) Given as a Single Agent and in Combination with Intravenous Pembrolizumab in Participants with Advanced or Metastatic Solid Tumors	Phase 1 Phase 2	October 31, 2024	Recruiting
**NCT04674683**	Study Comparing Investigational Drug HBI-8000 Combined with Nivolumab vs. Nivolumab in Patients with Advanced Melanoma	Phase 3	October 2025	Recruiting
**NCT04835805**	A Study to Evaluate the Safety and Activity of Belvarafenib as a Single Agent and in Combination with Either Cobimetinib or Cobimetinib Plus Atezolizumab in Patients With NRAS-mutant Advanced Melanoma	Phase 1	November 11, 2024	Recruiting
**NCT04526899**	Trial With BNT111 and Cemiplimab in Combination or as Single Agents in Patients with Anti-PD-1-refractory/Relapsed, Unresectable Stage III or IV Melanoma	Phase 2	June 2025	Recruiting
**NCT04557956**	Testing the Addition of the Anti-cancer Drug, Tazemetostat, to the Usual Treatment (Dabrafenib and Trametinib) for Metastatic Melanoma That Has Progressed on the Usual Treatment	Phase 1 Phase 2	December 4, 2024	Recruiting
**NCT04157517**	A Study of Modakafusp Alfa (TAK-573) Given by Itself and Together with Pembrolizumab in Adults with Metastatic Solid Tumors	Phase 1 Phase 2	August 2, 2023	Recruiting
**NCT04993677**	A Study of SEA-CD40 Given with Other Drugs in Cancers	Phase 2	October 31, 2025	Recruiting
**NCT02857270**	A Study of LY3214996 Administered Alone or in Combination with Other Agents in Participants with Advanced/Metastatic Cancer	Phase 1	July 31, 2023	Active, not recruiting
**NCT03776136**	Efficacy and Safety of Lenvatinib (E7080/MK-7902) Plus Pembrolizumab (MK-3475) for Advanced Melanoma in Anti-Programmed Death-1/Programmed Death-Ligand 1 (PD-1/L1)-Exposed Participants (MK-7902–004/E7080-G000-225/LEAP-004)	Phase 2	December 26, 2024	Active, not recruiting
**NCT03767348**	Study of RP1 Monotherapy and RP1 in Combination with Nivolumab	Phase 2	November 2024	Recruiting
**NCT03891953**	Study of Safety and Efficacy of DKY709 Alone or in Combination with PDR001 in Patients with Advanced Solid Tumors	Phase 1	April 15, 2024	Recruiting
**NCT04894994**	FLX475 in Combination with Ipilimumab in Advanced Melanoma	Phase 2	December 1, 2023	Recruiting
**NCT05544929**	A Study of Safety and Efficacy of KFA115 Alone and KFA115 in Combination with Tislelizumab in Patients with Select Advanced Cancers	Phase 1	September 15, 2025	Not yet recruiting
**NCT04514484**	Testing the Combination of the Anti-cancer Drugs XL184 (Cabozantinib) and Nivolumab in Patients with Advanced Cancer and HIV	Phase 1	November 2, 2025	Recruiting
**NCT03289962**	A Study of Autogene Cevumeran (RO7198457) as a Single Agent and in Combination with Atezolizumab in Participants with Locally Advanced or Metastatic Tumors	Phase 1	February 1, 2024	Active, not recruiting
**NCT02791334**	A Study of Anti-PD-L1 Checkpoint Antibody (LY3300054) Alone and in Combination with Participants with Advanced Refractory Solid Tumors	Phase 1	December 6, 2022	Active, not recruiting
**NCT03729596**	MGC018 With or Without MGA012 in Advanced Solid Tumors	Phase 1 Phase 2	May 2023	Recruiting
**NCT02419495**	Selinexor With Multiple Standard Chemotherapy or Immunotherapy Regimens in Treating Patients with Advanced Malignancies	Phase 1	December 31, 2022	Recruiting
**NCT04244552**	A Phase 1b Trial of ATRC-101 in Adults with Advanced Solid Malignancies	Phase 1	March 2025	Recruiting
**NCT04140526**	Safety, PK, and Efficacy of ONC-392 in Monotherapy and in Combination of Anti-PD-1 in Advanced Solid Tumors and NSCLC	Phase 1 Phase 2	December 31, 2024	Recruiting
**NCT04336241**	Study of RP2 Monotherapy and RP2 in Combination with Nivolumab in Patients with Solid Tumors	Phase 1	July 223	Recruiting
**NCT05259696**	Glycan Mediated Immune Regulation with a Bi-Sialidase Fusion Protein (GLIMMER-01)	Phase 1 Phase 2	June 2025	Recruiting
**NCT04165967**	Adoptive Tumor-infiltrating Lymphocyte Transfer with Nivolumab for Melanoma	Phase 1	August 2025	Recruiting

## Herbal drugs in skin cancer

### Various natural resources of anti-cancerous agents

The availability of natural resources for medical purposes is widely spread; more than 50% of drugs marketed in the current scenario is having a natural origin, even with more than 70% of anti-cancerous drugs marketed having a natural origin also. Due to their widespread availability and variety, plants have the primary raw materials for new medications and lead compounds for applications within the pharmaceutical science. Still, there are currently very few naturally acquired drugs marketed presently that focus on skin-related disorders, and there is not a single medication approved now for topical use [39].

Skin cancer with cutaneous malignant melanoma, which has a high mortality rate, is the most aggressive type. Malignant conditions can be treated in many ways, but due to the emergence of multidrug resistance, contemporary chemotherapy has a poor success rate of efficacy. So there is significance in finding a novel natural compound that is safe and efficient against melanoma skin cancer.

Majorly, anti-cancer agents are obtained from natural derivatives and their common mechanism of action for combating melanoma accompanies potentiating apoptosis, inhibiting metastasis, and inhibiting cell proliferation. The anti-melanoma effect of natural drugs is also mediated through a number of other mechanisms, including stimulation of activity of caspase, angiogenesis inhibition, and tumor-promoting proteins inhibition including Pl3-K, B-cell lymphoma 2 (Bcl-2), signal transducer and activator of transcription 3 (STAT3) and Matrix metalloproteinases (MMPs) [39].

#### Quercetin

Quercetin is a flavanol and the presence of -OH groups situated at 3, 5, 7, 3', and 4' of the flavanol framework indicates the flavanol quercetin. This flavanol is partially soluble in hot water, but practically insoluble in cold water and dissolvable in alcohol. The most prevalent flavanol in human nutrition is quercetin and which can be plant-derived in a variety of glycosidic categories, like arabinosides, glucosides, galactosides, and rhamnosides. Quercetin's major sources are tea, apples, tomatoes, grapes, Onions, and Ginkgo. Other derivatives are cloves, dark chocolate, capers, black elderberries, and oregano. The anti-inflammatory, as well as anti-oxidant properties of quercetin, are primarily responsible for its anti-cancer action [295].

Since quercetin can be found in a wide range of foods, studies have been done to find out whether these foods have offered any inhibitory activity against the development and progression of cancer. Until now, a variety of synergistic and antagonistic activities of quercetin had been identified, because of which quercetin operates biologically to enhance the anti-metastatic effects as well as chemo-protective effect. According to the studies, quercetin inhibits melanoma cell growth at low concentrations and induces apoptosis at high concentrations. Quercetin reduced the Bcl-2 expression as well as activity and enhanced the action and potential of caspase-3 within murine melanoma cells (B16-BL6) which led to the cells dying. In recent studies, it has been observed that quercetin suppresses UVB-influenced oxidative stress as well as DNA destruction, both of which promote and induces apoptosis within mouse epidermal cells [296].

The derivatives of quercetin such as Glycosides and polymethoxylate are considered good candidates to be utilized for topical application in quercetin because minute categories showed better metabolic stability, anti-inflammatory therapeutic indices, and activity in vitro. In vitro drug delivery to the skin using nanoparticle formulations as well as micro-emulsions consisting of quercetin has been showing promising results in skin cancer [297]. Mohammad Imran et. al. worked on the effectiveness of herbal components like Quercetin and resveratrol on skin cancer. The In-Vitro cell culture experiments revealed that the % cytotoxicity of quercetin and resveratrol NLC gel showed a higher antitumor effect than the conventional gel on A431 cell lines. The IC50 values of conventional quercetin and resveratrol and NLC gel of quercetin and resveratrol were observed to be 86.50 µM and 123.64 µM, respectively [298].

##### Kaempferol

It is the most general kind of flavonoid present in numerous categories of food products. It is sparingly soluble in water and is contained in tea, strawberries, green peppers, carrots, pumpkin, broccoli, propolis, brinjal, grapefruit, apples, beans, and onions. According to epidemiological data, dietary foods which are high in kaempferol content are associated with a decrement in the risk of cardiovascular diseases and cancer (including gastric, lung, ovarian, and pancreatic cancer). The anti-cancer mechanisms of kaempferol by which they show its anti-cancer activity are encouraging apoptosis and discourage cell proliferation. The G2/M phase of the choroidal melanoma cell cycle has been blocked by kaempferol [299].

Also, there are some researches published on the effectiveness of kaempferol for the treatment of cancer. The solar UV radiation induces the 90 kDa Ribosomal S6 Kinase (RSK) and Mitogen and Stress activated Protein Kinase (MSK) which are the family of proteins responsible for the downstream signal transduction of MAPK cascade mechanism. The Kaempferol inhibits these two families of proteins (RSK2 and MSK1) by binding at their ATP binding site and suppressing the Kinase activity. Ke Yao et. al. conducted research on the effect of kaempferol on Solar UV radiation-induced skin cancer. The obtained study results revealed that Kaempferol suppressed Solar UV-induced skin cancer markedly by acting via RSK2 and MSK1 in a mouse model. The 1 mg kaempferol reduces the tumor volume by up to 68% and the incidence rate by up to 91% [300].

##### Epigallocatechin-3-gallate

Epigallocatechin-3-gallate (EGCG), can be also termed as Flavan-3-ols which basically originate from tea, red wine, strawberry, and cocoa products, are the prime sources [301].

In melanoma cells, EGCG can cause apoptosis as well as cell cycle arrest (A374 and Hs-294 T). EGCG can follow the mechanisms by which it can produce various effects including the upregulation of Bcl-2 associated X protein (Bax), a pro-apoptosis protein, the activation of caspases-3, -7 and -9, downregulation of proteins that inhibit apoptosis, or cell survival-promoting proteins (Bcl-2, D1 and cyclin-dependent kinase 2 (cdk2), and through the induction of tumor-suppressor proteins (p16INK4a, p21WAF1/CIP1, and p2KP1). Other suggested mechanisms include inhibiting the activation of the downstream adapter protein and also the epidermal growth factor receptor in human skin cancer cells (A431) [302].

Also, EGCG is thought to be a promising candidate to be used as a photo protectant. Studies have shown that EGCG has a great ability to safeguard the skin from photoinduced destruction, which is the chief principle of cancerous skin, and that skin photo protection is a highly significant component in preventing skin cancer [39]. Maha, et al. worked on the EGCG-loaded nanovesicles including Ethosomes and Transferosomes (TEs) as well as Penetration Enhancer-containing vesicles (PEVs). these prepared nanovesicles were subjected to treatment on the skin cancer cell lines (A431), the study results revealed that the prepared PEVs showed higher chemopreventive and chemotherapeutic potential against skin cancer cell lines. The histological studies also showed better effectiveness of the EGCG nanovesicles compared to conventional EGCG drug solutions [303].

##### Apigenin

Apigenin, chemically described as 4',5,7, -trihydroxy flavone and its pure form produces yellow needle-like crystals and it is frequently present in oranges, celery, parsley, tea, and onions. Apigenin is categorized as the most bioactive flavone found in the natural system and epidemiological studies indicated, high flavone in diets lowers the chance of acquiring certain malignancies [296].

It was found that ethosomes containing apigenin, when compared to liposomes in vivo as well as in vitro, caused a greater deposition of apigenin under the skin. It was discovered that apigenin which had been nano encapsulated may be a more appropriate formulation than apigenin which was free because encapsulated apigenin has the ability to permeate the nucleus and maximize the apoptotic effects. In albino mice exposed to UVB, topical therapy of nano-encapsulated apigenin inhibited carcinogenesis [304]. Apigenin is a flavonoid that is supposed to inhibit UV-induced skin carcinoma by activating the AMPK pathway (AMP-activated protein kinase) leading to suppressed basal mTOR activity in the in-vitro cultured keratinocytes. Also, it was demonstrated that apigenin is supposed to inhibit the UVB-induced mTOR signaling leading to decreased cell cycle progression and cell proliferation in the mouse skin as well as cell culture of mouse skin keratinocytes [305].

##### Daidzein

Daidzein is also termed soy isoflavone and is primarily soluble in solvents that are alkaline in nature. It is a member of a group of compounds, called phytoestrogens which can demonstrate little chemo-protective nature towards the skin, whether according to an analysis, topical application of daidzein provides a very effectual photo-protection. In vitro studies provided the fact that daidzein was capable to hinder UVB-induced production of hydrogen peroxide within cells and hence they provide protection to the keratinocytes. In numerous investigations, genistein, as well as Daidzein, were examined as synergistic cytotoxic agents, and the results suggested that the 2 isoflavones had been effective when they combined together [296]. Daidzein is used as an anti-inflammatory, antioxidant activity, and also anticancer effect. Ugur, et al. developed Daidzein-loaded nanoemulsions and nanoemulgel by high-pressure homogenization technique with the droplet size of 149.80 ± 3.52 nm and 200.25 ± 11.09 nm respectively for nanoemulsion and nanoemulgel respectively, and the anticancer activity was assessed against melanoma. The cell culture experiments result on melanoma cells (B16) showed that the 100–150 µM of Daidzein induces 20–25% cytotoxicity. The mechanism behind the anticancer activity was supposed to be inhibiting the CDK2 (Cyclin-dependent kinase) in the G1 phase of the cell cycle. After treating the cell culture at some high concentration up to 100–200 µM the cytotoxicity was increased up to 20–40%, while no significant change in cell viability was observed at lower concentration of Daidzein [306].

##### ***β-***Carotene

Β-carotene is present in human diets predominantly. Its major sources include carrots, kale, pepper, spinach, pumpkin, sweet potatoes, and cantaloupe. Through the caspase cascade process, β-Carotene can activate apoptosis of melanoma cells in vitro by stimulating caspases-3, -8, and -9. The mode of action of β-carotene in murine melanoma cells may conclude the blockage of Bcl-2, p53, and caspase-3, which then stimulates apoptosis and potency of β-carotene upon tumor-specific angiogenesis, due to which tumor growth is affected. According to research, β-carotene may have prevented the stimulation or nuclear translocation of numerous transcription agents, which may have suppressed tumor-specific angiogenesis [307].

Freitas et al. performed research in which they compared the efficacy of β-carotene and β-carotene + resveratrol for the prevention of UV-induced skin cancer. After performing in-vitro skin penetration studies by Franz diffusion cell using Porcine ear skin it was observed that about 90% and 80% of β-carotene and antioxidants were penetrated in the subcutaneous layer after 12 h post application with 63% of skin retention. The β-carotene alone or in combination with the resveratrol reduced the skin penetration of UV radiation and improved sunscreen safety [308].

##### Resveratrol

Resveratrol, a group A stilbene is a phytoalexin antioxidant that occurs naturally. Stilbenes belong to the naturally occurring stilbenoid family of substances, which are distinguished by having dual aromatic rings connected via methylene bridge. It has a range of pharmacological activities, like anti-tumor activities, chemo-prevention, and cardio-protection. Red wine, red grapes, barley, and peanuts are the prime sources of resveratrol. Mojave yucca plant, as well as Japanese knotweed (Polygonum cuspidatum), are additional sources of resveratrol. Resveratrol is considered to have an impact on cancer induction in tumor development, promotion, and progression [309].

According to a study, resveratrol is preferred as a potential anti-cancerous property and comes in light to be discovered that it can suppress the proliferation of amelanotic as well as melanotic cells by activating apoptosis. Resveratrol has been shown to suppress the lipopolysaccharide-induced epithelial-to-mesenchymal transition, probably via regulating NF-κB signaling due to its anti-metastatic properties. As a result, radio-resistant melanoma cells have been seen to react positively to a treatment regimen combining resveratrol with radiation & there is also the possibility for resveratrol to be used as a radiation sensitizer in the treatment of melanoma. In comparison to radiation or resveratrol alone, the combined treatment proved more effective. According to in-vitro analysis, Resveratrol and temozolomide together have been found powerful cytotoxic representatives towards melanoma cells and primarily observed that it results in cell death when it participated in sensitizing melanoma cells to IL-2 immunotherapy & it appears that resveratrol functions effectively in collaboration with other treatment approaches [310].

##### Curcumin

Curcumin a yellow polyphenol, has been employed for both spice and medicinal purposes over the years. Curcumin’s anti-inflammatory and anti-oxidant abilities are considered to be its key medical benefits. The primary source of curcumin is Curcuma longa or turmeric. The mixture of curcuminoids contains only 2%–6% of curcumin. It has been evidenced that curcumin provides little efficacy towards a number of disorders including cancer, diabetes, epilepsy, psoriasis, and human immunodeficiency virus, etc., while also containing sufficient safety parameters at overdosing, and despite these realities still, there is no evidence for curcumin for its approval to be used as a medicine to treat any kind of diseases. Researchers have investigated that curcumin has the potential to treat various disorders and can be an effective therapeutic agent for regulating and treating cancer ailments like depression, pain, insomnia, and neuro-degeneration [311].

The mechanism of action of curcumin against tumors is facilitated by three different mechanisms: (i) downregulation of anti-apoptotic proteins which leads to the induction of apoptosis in tumor cells and upregulation of tumor suppressor genes (e.g., p53) (ii) downregulation of MMPs (matrix metalloproteinase), which prevents tumor invasion and (iii) the anti-inflammatory effect which increases its potency against tumors. In a recent study, it was identified that curcumin’s anti-melanoma activity was dependent on its ability to open the mitochondrial permeability transition pore (mPTP), which it had successfully done in vitro with melanoma cells. In melanoma cells, curcumin can cause time and dose-dependent apoptosis that is not dependent on p53 activation. Curcumin treatment of melanoma cells caused caspase-8 activation of the death receptor Fas-initiated Fas-Associated protein with Death Domain (FADD) apoptotic pathway. The outcome of topically administered curcumin was analyzed on a mice model by inducing cancer by UVB radiation. Topical treatments of curcumin have been found to delay tumor growth and reduce tumor extension as well as its broadness without producing any adverse effects, both before and after UVB exposure. However, no studies evaluating the usage of curcumin in human clinical trials have been reported [312].

##### Sulforaphane

Sulforaphane contains isothiocyanate that is present in cruciferous vegetables, like cabbage, Broccoli, radish, and cauliflower. Sulforaphane has been demonstrated to have anticancer effects including the capability to induce apoptosis, inhibit cell proliferation and prevent metastasis. By activating caspase 9, caspase 3, p53 protein, and the Bax gene, sulforaphane causes apoptosis. Further, it also deactivates the Bcl-2, Bid, and caspase 8 unintentionally and acts against the apoptosis. Sulforaphane has been shown to be anti-metastatic and to have the potential for use in cancer immunotherapy in In-vivo experiments using a mouse melanoma model. By promoting the cell-mediated immune response and up-regulating IL-2 and interferon-gamma (IFN-γ), while down-regulating IL-1β, IL-6, TNF-α and granulocyte–macrophage colony-stimulating factor (GM-CSF), sulforaphane suppresses the metastasis [39]. Sulforaphane is found in cruciferous fruits like cabbages, brussels sprouts, and broccoli. Its antioxidant effect is considered due to the activation of NRF2 (Nuclear Factor Derived-2 like 2) by inducing or overexpressing different cytoprotective proteins [313]. Cristiano Maria Chiara et el. Worked on the comparative efficacy of sulforaphane-free drugs vs sulforaphane-loaded nanovesicles like ethosomes and transferases for the treatment of skin cancer. The in-vitro skin permeation study results showed that the nanovesicles of sulforaphane had an increased skin penetration compares to free drug as well as after performing antiproliferative activity of free drug and nanovesicles it was found that the anticancer activity of Sulforaphane ethosomes has greater anticancer activity compared to free Sulforaphane after tested on SK-MEL 28 [314].

##### Hypericum perforatum

Hypericin is subjected as a very active agent for treating St. Hypericum perforatum or John’s Wort and is penanthroperylene quinine with photosensitive properties. Hypericin is a prime candidate to be used in photodynamic therapies for skin carcinoma due to its concentration and low dosage-dependent photo-sensitizing properties. The UVA-activated hypericin caused cell demising in melanoma by necrosis as well as apoptosis pathways. When hydro-alcoholic extract of H. perforatum was analyzed towards malignant melanoma cells of humans it was found to suppress the production of free radical formation, prevent cell proliferation and improve phototoxicity caused by UVA radiation. The polar methanolic extract of Hypericum perforatum is a prime candidate for future investigations because of its better performance towards physical and fluorescence activities, which could lead to its potential applicability to skin cancer via PDT [315]. PDT (photodynamic therapy) is one of the oldest techniques used for the management of skin cancer.Hypericum perforatum is a photosensitizer mostly used along with PDT due to its anticancer activity. Abd-El-Azim, Heba, et al. constructed the Hypericum perforatum-loaded lipidic nanocapsules (Hy-LNCs) as well as Hypericum perforatum-loaded hollow microneedles (Hy-HMNs) for sufficient intradermal delivery of the drug deep into the skin. the results showed that the Hy-LNCs have an encapsulation efficiency of 99.67% and drug deposition of 7 folds higher in the sin which resulted in 386 folds higher photoactivity. The Hy-LNCs were delivered deep into the skin through Hy-HMNs resulting in drastic destruction of skin cancer up to 85.84% after irradicating at 595 nm. Overall, the study showed that the Hy-LNCs through Hy-HMNs deep into the skin tissue followed by irradiation could be a good approach for managing skin cancer effectively, site-specifically, and minimally invasively [316].

##### Withania somnifera

Withania somnifera, often called Ashwagandha or Indian ginseng and comes a member of Solanaceae family of plants and the prime source of a group of naturally occurring polyoxygenated steroidal lactones known as withanolides. Withanolides show anti-proliferative effects in melanoma cell lines. Withaferin A is the most studied withanolide, which possesses a wide range of pharmacological activities and is useful in treating melanoma through hyperthermia. And in contrast to the reduction in the degree of thermotolerance, withaferin A increased tumor response during hyperthermia treatment. In a mice model, the combination of hyperthermia therapy and radiotherapy produce a non-toxic dose of Withaferin A also produced better therapeutic effects than radiation alone. Melanoma cells undergo apoptosis when Withaferin A is used alone due to its ability to down-regulate Bcl-2 and activate the formation of Reactive oxygen species (ROS) [39]. The Ashwagandha plant is rich in steroids, alkaloids, flavonoids, nitrogen-containing compounds, phenolics as well as trace elements. The clinical studies showed that the plant could be highly effective against various types of cancers including skin, lung, colon, prostate, kidney, and liver. The main anticancer activity is supposed to be due to withaferin A and Withanolide D with the least toxicity [317].

##### Melaleuca alternifolia

Tea tree oil, which is derived from the *Melaleuca alternifolia* plant, is widely familiar for many therapeutic activities, specifically for the skin. Terpinen-4-ol is the most important and potent chemical constituent of M. alternifolia which has anti-cancer properties. terpinene-4-ol or Tea tree oil have been shown to suppress the growth of melanoma cells in vitro via apoptosis, cell cycle arrest, necrosis, and prevention of cell proliferation at low doses which are not toxic to healthy fibroblast cells. The topical application of 10% tea tree oil in dimethyl sulfoxide (DMSO) has significant inhibition in tumor growth of mice bearing subcutaneous melanoma tumors. The oil and terpenes of *M. alternifolia* are indicated to prevent the proliferation of melanoma cells and tumors both in vivo as well as in vitro [318]. The studies showed that the combination of Melaleuca alternifolia with trametinib and/or dabrafenib induces cell apoptosis and reduces melanoma cell viability. The components of *M. alternifolia* plant responsible for anticancer activity are terpinolene, Alfa-Terpineol, and terpinene-40-ol, in which the terpinene-4-ol is having anticancer as well as pro-apoptotic activity [319].

The anticancer activity of terpinene-4-ol is supposed due to the induction of caspase-dependent M14 WT apoptosis of melanoma cells. The terpinene-4-ol interacts with the plasma membrane of the cell although another study showed that the anticancer activity of terpinene-4-ol was due to B16 melanoma, AE 17 mesothelioma when demonstrated in murine cell lines. They inhibit the cell cycle growth by arresting the G1cell growth cycle [320].

#### Rosmarinus officinalis

The herbal evergreen plant, *Rosmarinus officinalis* is also known as rosemary. Rosemary accommodates triterpene and phenolic diterpene anti-oxidants. The prime constituent of *R. officinalis* extract is phenolic diterpene i.e., carnosol. Metastatic murine melanoma cells were prevented from migrating towards the gel of basement membrane by carnosol and this effect was connected to the inhibition of MMP-9. At higher concentrations, carnosol has also been shown to restrict cell growth and viability. Ursolic acid, a pentacyclic triterpene is another active component of Rosmarinus officinalis. In both human and mice melanoma cell, it has been seen that ursolic acid activate the expression of the p53 protein and NF-κB activation which leads to apoptosis. Additionally, due to its inhibitory effects on the production of VEGF, MMP-2, MMP-9, and nitric oxide, ursolic acid act as a potent anti-angiogenic member in melanoma [39]. Also, some research suggests that Rosmarinus officinalis leaf extracts and some of their metabolites like indirubin (IND), prototype ligand 2,3,7,8-tetrachlorodibenzo-p-dioxin (TCDD), 6-formylindolo[3,2-b] carbazole (FICZ) and pityriazepin (PZ) inhibits the AhR activation (Aryl hydrocarbon receptor) in human keratinocytes. All *R. officinalis* leaf extracts showed dose-dependent agonist activity against AhR activation which makes them a potential candidate for the prevention and treatment of skin diseases caused by Aryl hydrocarbon receptor (AhR) activation [321].

#### Aloe species

The plant known as aloe is well known for its therapeutic uses and specifically in the treatment of skin irritation, laxative effects, and wound healing. The active chemical component of Aloe is an Emodin which is natural hydroxyanthraquinone that has been investigated for potential anti-melanoma activities. Emodin shows the anti-proliferation which is a time-dependent procedure and can prevent the MMP-9 effect of anti-melanoma. The anti-metastatic activity of Aloe-emodin therapy includes the inhibition of murine melanoma cell adhesion, invasion, migration, and aggregation. Aloin is another compound present in Aloe, that has the ability to block melanoma cell growth, interfere with cell adhesion pathways and act as a cytotoxic agent towards melanoma i.e. cisplatin [318]. Alex et al. evaluated the in-vitro anticancer activity of whole leaf extract on A375 cell lines. The MTT assay was carried out on the A375 cell lines using the gel of whole leaf extract of aloe species including *A. vera*, and *A. muth- muth* for the determination of their IC50 values. The results of the study showed that the selectivity index was found above 2, showing the activity against cancer cells. The A375 cell colonies were observed less in the Aloe group compared to the control group. Also, the *Aloe Vera* gel has higher anti-melanoma activity having less selectivity than dacarbazine [322].

#### Ingenol mebutate

*Ingenol Mebutate* can be extracted from the plant *Euphorbia peplus* which is getting approved by the U.S. FDA recently for treating actinic keratoses as a therapeutic agent after being investigated to have chemotherapeutic potential. According to preclinical studies, *Ingenol Mebutate* can cause apoptosis in mice by primary necrosis and mitochondrial swelling of the dysplastic keratinocytes. *Ingenol Mebutate*, a prime component of the *E. peplus* was studied in a phase I and phase II clinical study to examine its efficacy towards Basal Cell Cancers, Squamous Cell Carcinomas, and intra-epidermal carcinomas as a therapeutic agent [311].

Also, some researchers suggested that *Ingenol Mebutate* (IM) is a modulator of eight PKC (Protein Kinase C isoenzyme) which involved in the signaling pathways including cell angiogenesis, cell invasion, cell survival/ cell death (survival/autophagy), cell proliferation and acts as either tumor suppressor or oncogenes. IM induces two types of mechanisms depending upon the concentration including at high concentrations it induces PKC-dependent necrosis by activating neutrophils, antibodies, and anticancer T cells, and at lower concentration < 100 nM induces cell apoptosis [323].

#### Zingiber officinale

Ginger is a monocotyledonous herb in the form of rhizome and its botanical name is *Zingiber officinale*. The active constituent of *Z. officinale* is known as 6- gingerol, which has been tested for its capability to hinder the extension of melanoma and epidermoid carcinomas cells. According to studies, 6- gingerol has anti-proliferative, growth inhibition, and apoptosis induction effects on epidermoid carcinoma. By impacting the venous supply to the tumor, 6-gingerol suppresses the melanoma tumor growth, but it also causes cell death via the apoptosis in epidermoid carcinomas cells [318].

The studies suggested that the topical application of 6-paradol and 6-gingerol prior to 12-O-tetradecanoylphorbol-13-acetate (TPA) application on the back of shaven female mice showed inhibition of tumor burden in DMBA-induced skin cancer by inhibition of TPA-induced COX-2 in mouse which usually occurred due to the blocking of the p38 MAP kinase-NF-κB signaling pathway. Also, Shogaol one of the components of *Z. officinale* has a stronger inhibitory action on the SK-MEL2 human skin cancer cell lines [324].

#### Cannabinoids

*Cannabis sativa* is a plant that has been used for textile fibers; oleoresin and seed oil all of which have both therapeutic and psychotropic effects. With more than 100 compounds detected so far and it is the primary source of Phyto cannabinoids. Cannabidiol (CBD) and D9-Tetrahydrocannabinol (THC) is a prime phytochemicals found in *C. sativa* got a lot of attention recently. CBD has non-psychoactive effects in comparison to THC, which is advantageous for therapeutic uses of anticancer effects [325].

Cannabinoids are terpene phenolics that contain diphenol and a monoterpene moiety. These have been investigated in dermatology for psoriasis, itch, acne, atopic dermatitis, allergic contact dermatitis, scleroderma, and seborrheic dermatitis. According to the preclinical studies, cannabinoids show antitumor effects in melanoma and nonmelanoma skin cancer (see Table 5) [326]. The Phyto-cannabinoid D9- Tetrahydrocannabinol (THC) causes apoptosis, induce autophagy, and loss of cell viability, in melanoma cells. These effects were enhanced by the co-treatment of cannabidiol and these effects were proven in a melanoma xenograft mouse model. Additionally, D9- Tetrahydrocannabinol (THC) also reduces cell proliferation, metastasis, and angiogenesis in melanoma cells by inhibiting the oncoproteins Akt and pRbs [8, 326–328].

**Table 5:** Elaboration of antitumor effects of cannabinoids on melanoma and nonmelanoma skin cancer

**Cannabinoid** **Type Tested**	**Cancer Cell** **Line Tested**	**Results**
D (9)-tetrahydrocannabinol	BRAF wild-type melanoma cell line CHL-1 BRAF-mutated A375 and SK-MEL-28 melanoma cell lines	Activation of autophagy and apoptosis, loss of cell viability
JWH-133 synthetic cannabinoid	Human melanoma cell line A2058	Activation of CB2 receptors with JWH-133 reduced adhesion of melanoma cells to brain endothelial cells and decreased trans-endothelial migration rate of melanoma cells
THC WIN-55,212–2	Murine melanoma cell line B16 Human melanoma cell line Meljuso	Decreased melanoma cell viability and increased apoptosis in vitro. Decreased tumor cell growth, proliferation, angiogenesis, and metastasis in vivo

According to a famous Canadian marijuana activist who established his method of extracting oil from cannabis for promoting its homeopathic uses in various types of skin cancers such as melanoma cancer and Basal Cell Cancer and the oil is extracted from potent sedative Indica strains that consist of about 90% of THC level. However, it is illegal to produce oil from cannabis, and there are no clinical trials conducted for its efficacy and safety in humans [326].

#### Retinoids

Retinoids are derivatives of vitamin A, with widely determined functions in bone development, ocular physiology, reproduction system, and the immune system. Retinoids are known for acquiring pro-differentiating and antiproliferation activities. Isotretinoin (13-cis-retinoic acid) is the most effective retinoid for the inhibition of non-melanoma skin cancer [329]. The retinoid derivatives mainly include 13-cis-retinoic acid (CRA), Retinoic acid ethyl ester (RAEE), Trans-Retinoic acid (TRA), and Bexarotene (BXT). A study showed that the 2 mg/kg/day dose of CRA significantly reduced incidence of the new skin cancer when the dose was administered for 2 years. While RAEE due to its ester nature gets stored in the fat of the body for a longer period of time which is further detected after discontinuation of therapy after several years. BXT is FDA approved drug for the treatment of Cutaneous T-cell lymphoma with an oral dose range of 300 mg/m2/day. The use of retinoids (Isotretinoin) in chemotherapy may reduce the chances of the development of multiple non-melanoma skin cancers [330]. These Retinoids are supposed to act by binding to the nucleic receptor (NR) of the Retinoic acid receptors (RAR) subfamily like RARα, RARβ, and RARγ. This RAR acts as ligand regulated transcription factor by forming a heterodimer with retinoid X factor (RXR) by binding to the RA response elements (RAREs). RARs activate the signaling pathway called kinase and have a non-genomic effect. The mechanism involved in the prevention and treatment of skin cancer by Retinoic acid (RA) involves disruption of the RA signaling pathway via non-hematological and hematological malignancies which are also involved in breast cancer, lung cancer, leukemia, ovarian cancer, and prostate cancer. It is considered as RA derivatives also have anti-oxidant, pro-apoptotic, and antiproliferative effects in humans [330].

#### Dietary and herbal supplements

There is an increasing interest in the application of dietary and herbal supplements for the prevention and treatment of skin cancer. Some common dietary supplements and many herbal supplements are good sources of antioxidant and anti-inflammatory compounds. However, most of the phytochemical activity against cancer is related to either their apoptotic or antioxidant activity. In the mice model, the administration of antioxidants in combination seems to be more effective than the protection by antioxidants alone. Additionally, high dietary fat consumption shortened the time interval between UV exposure and the development of skin cancer. Apoptosis is a self-defense activity by which the body throws out dysfunctional cells such as metastatic malignant cells [329]. Table 6 represents the list of various herbal drugs used in the management of skin cancer.

**Table 6:** Illustration of herbal drugs used in the treatment of various kinds of skin cancer

**Sl. No**	**Name of Herbal drug**	**Carrier system in which the drug is encapsulated**	**Type of skin cancer on which drug is used for**	**Properties or characteristics of the herbal drug**	**References**
1	Syzygium aromaticum essential oil (SAEO) or Eugenol	Chitosan nanoparticles	Breast cancer, melanoma skin cancer	Mainly Having antioxidant properties	[331]
2	Cellulose	Nanoskin bacterial cellulose	Basal cell carcinoma	By providing anticancer nutrients(beta-glycan) to the cancerous skin tissues	[332]
3	Curcumin	Chitin nanogels	Skin cancer including melanoma and non-melanoma	Shows cytotoxicity & apoptotic effect on cancerous tissues	[333]
4	Ipomoea pes-caprae	Administered directly along with chemo and radiotherapeutics	Melanoma skin cancer	Anticancerous potential	[334]
5	Sanguinarine	Nanoparticles	Melanoma and non-melanoma skin cancer	Shows cytotoxicity	[335]
6	Ruta graveolens	Ethanolic nanoparticles or ethosomes	Melanoma skin cancer	Chemopreventive properties	[336]
7	Arabinoxylan	Nanocomposite hydrogels	Melanoma skin cancer	Showing anticancer activities	[337]
8	Scutellaria bardata (Lamiaceae)	nano drug	Skin cancer cells	Anti-inflammatory and antitumor activity	[338]

#### Caffeic acid

It is also one of the polyphenolic compounds which has shown enormous therapeutic potential in cancer. It can be derived from almost all plants, especially coffee, thyme, and olive plants. The properties such as antitumor, antioxidative, and anti-inflammatory make this compound a potential option for diseases which are induced by any kind of exogenous and endogenous stress [339]. The antioxidant role protects cells from oxidative damage caused by reactive oxygen species (ROS). ROS can induce DNA damage and promote the development of cancer [340]. By scavenging ROS, caffeic acid may help in the prevention of initiation and progression of NMSC. Whereas by modulating of signalling pathway, it inhibits the activation of epidermal growth factor receptor (EGFR) pathway, which is known to play a crucial role in the growth and survival of cancer cells [341]. By interfering with these signalling pathways, caffeic acid may disrupt the molecular mechanisms that promote non-melanoma skin cancer [342]. There are several scientific evidence that demonstrates the role of caffeic acid and its derivates in the inhibition of progression and development of cancer through their distinct mechanisms. To exemplify this, Balupillai et al*.,* showed the chemotherapeutic efficacy of CA on tumor bearing mice, and the findings demonstrated that CA downregulated iNOS, VEGF, and TGF-beta, and upregulated p53, resulting in the reduction of the metastasis of tumor cells. This study was conducted on UV-B induced carcinogenesis mice by activating anti-inflammatory transcription factor (PPAR γ) [343]. In another study from the same team, findings suggested that CA prevents UV-B induced photodamage through the activation of PTEN expression in human dermal fibroblast and mouse skin [344]. In addition, the researchers have explored the role of CA with some synthetic anticancer activity (combination therapy) and showed the promising outcomes by suppressing the proliferation, survival, and tumor growth in prostate cancer [345]. Another study conducted by Li et al*.,* showed the synergistic anti-tumor effects of CA when delivered in combination with 5-FU. Interestingly, the combination of CA and hydroxydaunorubicin (DOX) showed the synergistic therapeutic effects against lung cancer cells [346]. The findings demonstrated that the inhibitory effects were better with the less dose of two drugs as compared to double dose of single drug. In this study, CA was augmenting the effects of DOX by inhibiting proliferation, migration, and inducing apoptosis [339]. Such various evidence validates the application of CA in downregulating molecular pathways which are responsible for the initiation of inflammatory conditions which leads to the development of precancerous lesions.

### Molecular Targets for Natural Chemo preventive Agent

A natural chemo-preventive agent activates various cell signaling pathways and these pathways differ for different agents. However, depending on these cell types, the same molecule activates signaling pathways differently.

#### P53 family members

In response to many genotoxic stresses, the tumor suppressor P53 significantly regulates the cell cycle, apoptosis, DNA repair, and genomic integrity. P53 can bind to DNA sequences and activate the expression of target genes after its activation and is divided into four groups: Cell cycle inhibition, apoptosis, inhibition of angiogenesis, and genetic stability. P53 can also function as a transrepressor due to its trans-activation function. Various natural chemo-preventive agents cause apoptosis or cell cycle arrest by activating P53 and its target genes. For example; Curcumin induces P53-mediated apoptosis by activating mitochondrial translocation. Additionally, EGCG also stimulates the P53 and BAX in breast cancer cells [347]. The vitamin D receptors (VDR) are considered tumor suppressors in the skin which are the prime element of the Vitamin D endocrine system involving CYP27B1, CYP27A1, and VDR. These receptors are overexpressed in the non-melanoma skin cancer condition (NMSC). This anticancer effect of the VDR family is associated with the p53 family (p53/p63 and p73) in which ligand-induced growth inhibitory effect is seen. This p53 family interacts in a highly coordinated manner in response to cell hemostasis to a large manner, involving UV-induced DNA damage which is a sign of photocarcinogenesis [348].

#### Nuclear Factor- Kappa B

The master transcription factor nuclear factor- kappa B (NF- κB) is comprised of closely related protein that usually occurs as dimers and binds to a typical DNA sequence within the promoters or enhancers of target genes known as the κB site. NF- κB is stimulated by free radicals, cytokines, tumor promoters, inflammatory stimuli, carcinogens, Endo-toxins, γ- radiation, x- rays, and UV lights and induce NF- κB target genes which are essential for transformation and cellular growth, metastasis, suppression of apoptosis, invasion, inflammation, chemo-resistance, and radio-resistance. Mostly chemopreventive agents including curcumin, EGCG, resveratrol, and genistein are potent NF- κB pathways inhibitors [347]. Nuclear Factor Kappa-β (NFKβ) is the major transcription factor present in the cell cytoplasm which on activation moves to the nucleus. The activation mainly happens due to smoking, stress, and exposure to bacteria, viruses, carcinogens, free radicles, inflammatory stimuli, endotoxins, and tumor promotors. Around 400 genes were expressed upon activation of NFKβ including enzymes (iNOS and COX-2) and cytokines (chemokines, IL1, IL6, IL8, and TNF), cell cycle regulatory entities, angiogenic factors, and viral proteins. NFKβ is linked with most human diseases like diabetes, asthma, Alzheimer’s disease, cancer, AIDS, and atherosclerosis. The NFKβ is mainly responsible for the development and progression of cancer [349].

#### Activator protein-1

A group of dimeric basic region leucine zipper proteins comprising Jun, Fos, Maf, and ATF subfamilies is knowns as activator protein-1 [AP-1]. These proteins can bind to cAMP response elements or AP-1 DNA recognition sites to form homodimer or heterodimer and which activates their target genes. AP-1-activated genes of important modulators of angiogenesis, invasion, metastasis, proliferation, apoptosis, differentiation, and survival.

Various naturally occurring chemo-preventive molecules like curcumin, resveratrol, and green tea act by inhibiting AP- Activation & activates the AP-1 target genes, which subsequently shows its chemo-preventive potential [347]. Activator Protein-1 is a key protein from family Jun (c-Jun, JunB, and JunD) Fos (c-Fos, FosB, and FosD) responsible for the proliferation as well as differentiation of cells. Several diseases including cancer and inflammatory diseases occur due to failure of expression of Activator Protein-1. Targeting activator protein 1 signaling pathway by bioactive natural agents is a possible therapeutic strategy for cancer prevention and intervention [350, 351].

#### STAT (Signal Transducers and Activators of Transcription Pathway)

The studies of transcription activity in response to interferon have identified a unique signal transduction pathway to the nuclear. Activation of different tyrosine kinases causes effect as dimerization, phosphorylation, and nuclear localization of the STAT proteins, binding to specific DNA regions, and direct transcription. Several natural agents, such as Curcumin, resveratrol, and green tea regulate the STAT activation in tumor cells [347].

STAT is the pathway that is directly connected to the family of proteins called JAK (Janus Kinase) which is involved in the integration of different signaling pathway’s signals. STAT3 and STAT5 are majorly involved in the progression of cancer while STAT1 plays a crucial role in tumor suppression. Chronic inflammation occurs due to persistent STAT3/STAT5 activation which leads to increased susceptibility of healthy cells and the development of cancer [352]. STAT1-STAT4, STAT5A, STAT5B, and STAT6 are the seven genes of the STAT family which are mainly involved in apoptosis, cell proliferation, immune response, and angiogenesis. The mutation in STAT3 leads to chronic natural killer lymphoproliferative disorder (CLPD-NK) and T-cell large granular lymphocytic leukemia (T-LGL). STAT5 mutation leads to CLPD-NK but compared to STAT3 in smaller proportion [353].

### Benefits of phytopharmaceuticals

Bioactive substances originating from plants and herbal items are known as phytochemicals. As they have anti-carcinogenic properties and are easily accessible, affordable, and well-tolerated, these substances seem to be helpful in the fight against cancer. According to evidence, phytochemicals' anti-oxidative, anti-inflammatory, anti-proliferative, anti-metastasis, & antiangiogenic activities are what give them their anti-carcinogenic capabilities [40].

Several phytochemicals contain polyphenol groups, which are made up of numerous hydrophilic hydroxyl groups and function as free radicals & ROS scavengers. By doing this, cells are prevented from suffering oxidative damage to their DNA, proteins & lipids. And some other phytochemicals have anti-inflammatory effects by preventing the release of inflammatory mediators or cytokine activity, which stops the host cells from becoming inflamed. Additionally, phytochemicals alter a number of cell signaling pathways and prevent cell proliferation and angiogenesis [354].

Combining chemotherapeutics with phytopharmaceuticals can lead to the reduction of dose and improved chemotherapeutic efficacy. This would reduce the dose-related toxicity. They will manage skin cancer in the future because of the special function of phytopharmaceuticals in the treatment of skin cancer or carcinoma cancer by targeting various molecular pathways without harming other normal cells [355]. A combination approach can provide synergy which ultimately would reduce the dose of drugs and lethality involved with the currently used chemotherapeutics [356].

Since the phytopharmaceuticals used would not have site-specific targeting therefore the use of nanotechnology-based drug delivery systems is being learned about. This can address a number of safety issues including biocompatibility, potential toxicity (of unknown natural substances), and a dearth of clinical studies on medicinal plants and herbal remedies [357–359].

## Nanotechnological approaches to tackle skin cancer (Critical attributes for the development of nanotechnology-assisted drug delivery systems for enhanced outcomes)

The development of nanotechnology-driven advanced drug delivery systems is based on various parameters which are critical on the different stages of the design and development of these nanoformulations for various diseases including non-melanoma skin cancer. First and foremost, the composition of nanovehicle is highly critical for the design of a stable and effective nano-delivery system. There are some important variables/attributes on which the designing of a nanosystem is dependent and their successful delivery to the skin. Such variables are: (a) The nature of excipients (lipids, emulsifier, and stabilizer); (b) Experimental conditions (temperature, rpm, humidity, gases condition); (c) Instruments/machines/equipments involved in designing and development [360]. There might be some other attributes of the human skin which also affects the delivery and overall chemotherapeutic efficacy. The pharmacodynamic effects such nanovechicles is also dependent on the route of delivery (topical/intravenous/intratumoral/subcutaneous) [361]. To achieve the desired pharmacological effectiveness, the nanovehicle should carry a drug and absorbed at the site of administration such as skin. If the desired delivery is locally/topically, nanovehicle will exert effects locally, but there are chances when these nanocarriers absorbed deeper into the systemic circulation [362]. Interestingly, the free therapeutics/API is always responsible for any therapeutic efficacy; however, the excipients involved in the designing of nanocarrier could enhance the effectiveness and cause the toxicity by modifying the chemistry of nanocarrier [363]. The extent of the API or solvent absorbed in the skin defines the severity of the exposure which is dependent on:

- Normal or diseases condition of the skin / pathophysiology of the skin
- Time of exposure
- The surface area of dose application
- Location of dose application
- Dose of applied nanocarriers along with API
- Physiochemical features of API/Ingredients
- Clearance

These factors are crucial as these could influence the performance of development drug- loaded nanocarrier by altering the pharmacokinetic and pharmacodynamics of therapeutics/API [364]. There is plethora of ingredients which are already approved by regulatory authorities such as USFDA, EMA, and TGA to involve them in the design and development of nanocarrier. These ingredients are considered safe and categorises as Generally Recognised as Safe (GRAS) by USFDA. Also, the pharmaceutical and biotechnology industries follow the guidelines provided by regulatory agencies to develop safe, stable, and effective product. Therefore, every aspect of formulation from the design tom administration/application route play key role in confirming the effectiveness of drug-loaded nanocarrier.

### Nanotechnology

During the last few years, numerous advances have been made in the area of nanotechnology, notably in the utilization of nanotechnology in medicine**.** Nanotechnology is one of the most visible developing technologies, with great hopes in a variety of disciplines influencing daily life [365, 366]. Nano-agents offer novel ways for the treatment of an inclusive range of diseases because of their distinctive ability to enhance drug delivery. Nano-agents are nano-scale machines (i.e., molecularly precise in structure and function) that work on biological cells to increase drug distribution inside a patient's body [367]. Lots of researchers and scientists are currently working on different therapies or disease conditions to prove the dominance of nanocarrier therapy or nanotherapeutics in the drug delivery system.

### Nanosystems in the diagnosis of skin cancers

Diagnosis at an early stage can slow down the growth and metastasis of cancer cells. The collaborative efforts of the American Joint Committee on Cancer (AJCC) Staging Manual (eighth edition) and the National Comprehensive Cancer Network (NCCN) guidelines version 2.20225 establish a well-grounded framework rooted in evidence for the diagnosis and management of skin cancer [368, 369]. The process of clinical investigation commences with a comprehensive review of the patient's medical history and a thorough physical examination. It is strongly advised to conduct total body examinations, as it is common for patients to present with multiple instances of skin cancer. Noteworthy factors contributing to the risk of skin cancer comprise advanced age, an increased count of benign moles (relevant to melanoma), red hair, and fair skin coupled with a history of intense intermittent ultraviolet (UV) exposure (pertinent to melanoma) or chronic cumulative UV exposure (associated with SCC) and instances of immunosuppression. Furthermore, a personal history of skin precancerous conditions or a family history serves as a significant risk indicator. Conducting a thorough examination of skin offers and educating patients about identifying potential early signs of concerning lesions.

Skin cancer can be identified through visual observation or specific inspection. The Fitzpatrick scale one of measurement aids that recognizes the type of skin cancer in individuals who have an elevated susceptibility to sunburn and its resulting complications. Despite constituting just 1–2% of the population, individuals with red hair account for 16% of melanoma patients. Observable effects from sun exposure, such as wrinkles, telangiectasia (visible blood vessels), solar elastosis (yellow/white skin puckering), and irregularities in pigmentation, must be acknowledged factors. Further, specific inspection dermoscopy to magnify and light (non-polarized and polarized) for accurate diagnosis and analysis. When a suspicious lesion is detected, it is palpated and reported then, Individuals at a heightened risk should consider undergoing comprehensive imaging techniques, such as whole-body photography and digital dermoscopy, commonly referred to as mole mapping [370].

#### Nano-biosensors

Biosensor or nano biosensor technology is often used for the identification of skin disease indications. In the physical evidence of a living thing, nano-sensors are used as perceptive tools. Although, nanomedicine has a substantial impact on the design of nano-sensors. Theoretically, cancer diagnostics might be established using point-of-care tests using biosensors, which are frequently used tools for electrochemical biomarker detection [371]. Electrochemical identification of functional melanoma biomarkers is an effective and low-cost detection technology for melanoma cancer diagnosis that facilitates better sensitivity and specificity. Antibodies that are electrochemically transducer-anchored are used to build immunosensors, which are useful for cancer diagnosis. The production of sensitive and stable materials by immobilizing biomolecules is likely to be a crucial aspect of the development of electrochemical immunosensors. Because of their great adsorption efficiency and large surface area, nanoparticles may efficiently immobilize biological macromolecules with excellent biological stability and activity [358, 359]. Seenivasan et al. created a novel immunosensor for the early detection of melanoma cancer using SiO2 NPs and polypyrrole nanocomposite [372].

There are some advantages of nano biosensors including their ability to identify cancer-related genetic alterations at an early stage, frequently before any outward signs occur. Early detection for prompt treatment and intervention. Targeting skin cancer biomarkers can be done using nano-biosensors. Accurate diagnosis of diseases by detecting even minute changes in these biomarkers due to their small size and great sensitivity. Information regarding a patient's reaction to treatment can be obtained through nano-biosensors, which enable alterations to treatment regimens based on the requirements of each patient, eliminating superfluous procedures and potential adverse effects. Since nano-biosensors have wireless data transmission capabilities, healthcare providers can monitor patients remotely. Patients in rural or underserved areas will particularly benefit from this. Various drug delivery systems can incorporate nano biosensors to enable targeted and localized treatment. This method maximizes the impact on malignant cells while minimizing the side effects or toxic effects on healthy tissue [1, 2].

Apart from the wide advantages they have some challenges including difficulty to manufacture the nano biosensors with excellent sensitivity, stability, and specificity. Patient privacy and the security of transmitted health data are raised by remote monitoring. Medical nano biosensors must undergo extensive testing and regulatory approval procedures to ensure their effectiveness and safety. Also, it is mandatory to ensure that nano biosensors are biocompatible and do not have negative effects when in touch with skin or tissue [1, 2]. There are some applications in which several biosensors are for the detection of several diseases Lactate dehydrogenase (LDH) is the earliest prognostic biomarker of melanoma as well as serum s100B level, BRAF V600, Tyrosinase mRNA are the biomarkers used for the detection of melanoma. Some of the wearable biosensors are used nowadays which are basically non-invasive microneedle sensors that electrochemically detect the tyrosinase level in the body. The catechol-loaded carbon paste is filled into the hollow microneedles which measure the concentration of tyrosinase in the body tissue [372].

Almawgani et al. designed a nano-biosensor with high sensitivity comprising photonic crystals for the detection of cancer. They used (Si/ SiO2)N/Defect/(Si/ SiO2)N as a cancer detector in the 1-D binary photonic crystal with a quality factor (QF) of 3745.64. The detector works on the principle of refractive index, in which the patient’s blood was used as a detector layer for the detection of cancerous cells. The shift in the refractive index at the higher wavelength was observed in the blood samples containing cancer cells in contrast to the normal blood sample. The sensitivity of the proposed nano biosensor was found to be 2400.08 nm/RU, when compared to the most modern biosensors, this sensitivity is incredibly high. The incidence angle and the detector thickness were the prime factors for the sensitivity of the proposed nano biosensor design. The suggested binary photonic crystal's photonic bandgap expands as the incidence angle widens, oscillating as the defect layer thickness extends [3].

##### Quantum dots

Quantum dots have attracted considerable interest in a variety of sectors, including medicine, because of their potential for use in therapies and diagnostics, particularly the treatment of skin cancer. Quantum dots may be functionalized with biomolecules, such as peptides or antibodies that target cancer cells or skin cancer-related biomarkers. These functionalized quantum dots can specifically bind to cancer cells when applied to the skin. Quantum dots can emit light of various hues when exposed to light wavelengths because of their special fluorescence characteristics. The reliable identification of skin cancer lesions using high-resolution imaging is possible with the help of this fluorescence. With the help of this technique, doctors can see the cancer's extent and make treatment plans. One can employ quantum dots to track how well a treatment is working [355]. Researchers and medical professionals can instantly monitor the efficacy of treatments by conjugating them with certain molecules that react to modifications in the tumor microenvironment. They can precisely deliver therapeutic cargo to malignant cells by functionalizing quantum dots with targeted molecules. This method of tailored medication administration boosts therapeutic effectiveness while minimizing adverse effects on healthy cells [356]. Quantum dots have some challenges including the determination of the proper light dosages and wavelengths for efficient treatment without harming healthy tissue. To ensure patient safety, a thorough study of the long-term effects of quantum dots on the skin and body is required. For the sake of reducing the possibility of hazardous or unfavorable reactions, the materials utilized to make quantum dots must be biocompatible [357].

These are nanocrystals having colloidal fluorescent semiconductors with a size range of 2-10 nm. They feature an extensive absorption band, often symmetrical, as well as a small emission band in the visible to near-infrared spectral region (NIR). The inner core of quantum dots is often made up of periodic table elements (groups II-VI) or III-V covered with a zinc-sulfate film (ZnS**)** [14]. The utilization of these semiconductor nanocrystals in a wide range of cancer management and therapeutic applications is very promising. These are highly successful because the quantum dots' wide absorption and narrow emission properties enable multicolor imaging with a single excitation source [373].

Nie et. al revealed that in animal models, prostatic membrane antigen-targeted quantum dots can simultaneously target and scan prostate cancers. To increase quantum dot solubility, sensitivity, selectivity, and visibility in the target tissue, the quantum dot surface may be designed or altered. However, due to their heavy metal content as well as a few instances of cytotoxicity, quantum dots have been subjected to toxicological investigation. The toxicity of quantum dots is determined by a number of parameters coming from both intrinsic physicochemical features and environmental settings. According to in vitro analysis it is specified that quantum dots may be harmful, with the majority of the toxicity related to surface coating [374].

Subsequent research found that surface modification with N-acetylcysteine decreased quantum dot toxicity, but unmodified quantum dots caused lipid peroxidation in cells. Several researchers have studied the issue of toxicity and discovered that biocompatible surface coatings such as PEG or micelle encapsulation can limit the release of harmful metals [373]. Fakhri Ali et. al. developed Cellulose fibers decorated Fe_3_O_4_-Ag_2_O Quantum dots on which Etoposide and methotrexate were grafted. These developed Fe_3_O_4_-Ag_2_O Quantum dots showed ferromagnetic properties with 78.94% and 63.84% release of Etoposide and Methotrexate respectively. The study results showed that the cytotoxicity of developed Fe_3_O_4_-Ag_2_O Quantum dots has low cytotoxicity over conventional drug therapy showing its safety to the body tissue [375].

The carboxy-coated quantum dots (QD655) were developed by Saulite et al. and later tested on nano-engineered mesenchymal stem cells (MSCs). The tri-lineage differentiation, cell viability assay, and Ki67 expression analysis were used to evaluate the QD655's efficacy. Additionally, utilizing endocytosis inhibitors and fluorescence microscopy, the cell uptake of the QD655 was investigated in terms of phagocytosis, micropinocytosis, clathrin- and caveolin-dependent endocytosis lipid-raft pathways. Which suggested that the primary cell uptake pathway for QD655 into MSCs was Claritin-dependent endocytosis. They were found in early endosomes after 6 h and in late endosomes and lysosomes after 24 h, where their concentrations ranged from 0.5—64 nM, making them an ideal candidate for targeted therapy [376].

##### Nanotubes

Carbon nanotubes are a kind of fullerene that is made up of coaxial graphite sheets twisted up into barrels or cylinders. With high cytoprotective and anti-oxidant activities, carbon nanotubes (CNTs) are the most stable molecules. A highly effective biomarker sensor for early-stage infection detection and skin melanoma diagnosis was created using the conductivity of CNTs. Shvedova et al. used immortalized keratinocyte cell culture for analyzing and screening the effects of carbon nanotubes. Researchers were able to investigate the effects of prolonged single-wall carbon nanotube (SWCNT) exposure on cellular toxicity and oxidative stress by evaluating cell survival, production of free radicals, and the accumulation of peroxide-related metabolites. In cultured skin cells, exposure to SWCNT also modifies their morphology and ultrastructure. It has been determined that skin contact with unrefined SWCNT may result in skin toxicity due to the skin's enhanced oxidative stress [377].

Using nanomaterials in photothermal treatment, Moon et al. assessed the effectiveness of the technique for focusing on cancer. SWNT is a viable and effective option for use as a photothermal agent. This work reveals in vivo tumor obliteration by SWNT targeting and NIR irradiation. The mice which are photothermally targeted reveal a full tumor cure for 6 months without a recurrence phase. In around two months, the majority of the SWNTs that intervened were removed from the mice. According to the studies, SWNTs are an effective photothermal agent and are a step toward future cancer therapies [190].

Sahoo et al. created multi-walled carbon nanotubes and graphene oxide generalized with extremely hydrophilic and biocompatible compounds in order to load and target the anti-cancer agent CPT camptothecin. Through pi-pi interactions, the Multiwall carbon nanotubes /polyvinyl alcohol (MWCNT-PVA) and graphene oxide/ polyvinyl alcohol (GO-PVA) were encapsulated and demonstrated the power to eradicate skin as well as breast cancer cells [378].

In order to kill the in-vitro A375 human skin cancer cells, Yan Mi et al. prepared multi-walled carbon nanotubes (MWCNTs) with a high aspect ratio and improved electrical properties. This combination of high field strength E (2–10 kV/cm) and nanosecond pulsed electric field (pulse width- (100–500 ns) and pulse number N (5–260)) reduced the high field strength E. The study's findings demonstrated that the field intensity, pulse number, and pulse width all have a favorable impact on the viability of A375 cells. The link between cell viability and pulse energy density was represented by an S-shaped graph. Additionally, converting the MWCNTs to a 1-dimensional structure with good electrical properties does not change the MWCNs' basic capabilities but can considerably improve the effectiveness of nanosecond pulsed electricity to kill cancer [379].

To investigate the uses of carbon nanotubes (CNTs) and functionalized carbon nanotubes (FCNTs) as drug delivery vesicles, Mirsalari et al. used molecular dynamics simulations to explore the encapsulation and adsorption of the anticancer medication dacarbazine I in aqueous medium. They studied four types of preparations viz., Dacarbazine incorporated into the inner surface of pristine and carbon nanotubes (DAC-CNT_in_), Dacarbazine incorporated into the inner surface of pristine and functionalized carbon nanotubes (DAC-FCNT_in_) in which one molecule of Dacarbazine at the center of the pristine single-walled and functionalized CNTs pore in the system, Dacarbazine incorporated onto outer wall of the pristine and Carbon nanotube (DAC-CNT_out_), and DAC-FCNT_out_ in which one dacarbazine molecule was on the exterior surface of the pristine and functionalized CNTs in the system. According to the data, after spontaneous encapsulation, pristine and carboxylated carbon nanotubes can provide an excellent contender for drug delivery vehicles for skin cancer medications. Additionally, the favorable position was suggested by dacarbazine being enclosed inside the CNTs and FCNTs rather than adsorbing it to their exterior surfaces [380].

### Nano-system in the therapy of skin cancer

#### Inorganic nanoparticle

##### Iron oxide

The Superparamagnetic iron-oxide NPs (SPION) have been explored for drug targeting and other biological application because of their improved drug encapsulating capacity, high magnetic responsiveness, and increased targeted delivery efficiency. According to the literature, the structure and composition of skin prevent materials with a molecular weight of more than 600 Da (> 600 Da) from passing through the skin layers. New advancements in nanomedicine have made it possible to create several forms of MNPs (magnetic NPs) with few nanometers [381].

Rao et al. created the EPI-SPION (epirubicin encapsulated iron oxide NPs) which was targeted to skin carcinoma transdermally. The SPION was modified to produce a drug carrier that used magnetism in the chemotherapy of skin cancer. The WM266 and HaCaT keratinocyte cell lines study of skin cancer revealed that SPION had high compatibility. The in vitro study assessed the ability of the EPI-loaded SPION to penetrate the skin. The study revealed that iron oxide NPs have the potential for efficient transdermal administration in skin melanoma [382].

**Fig. 12:**
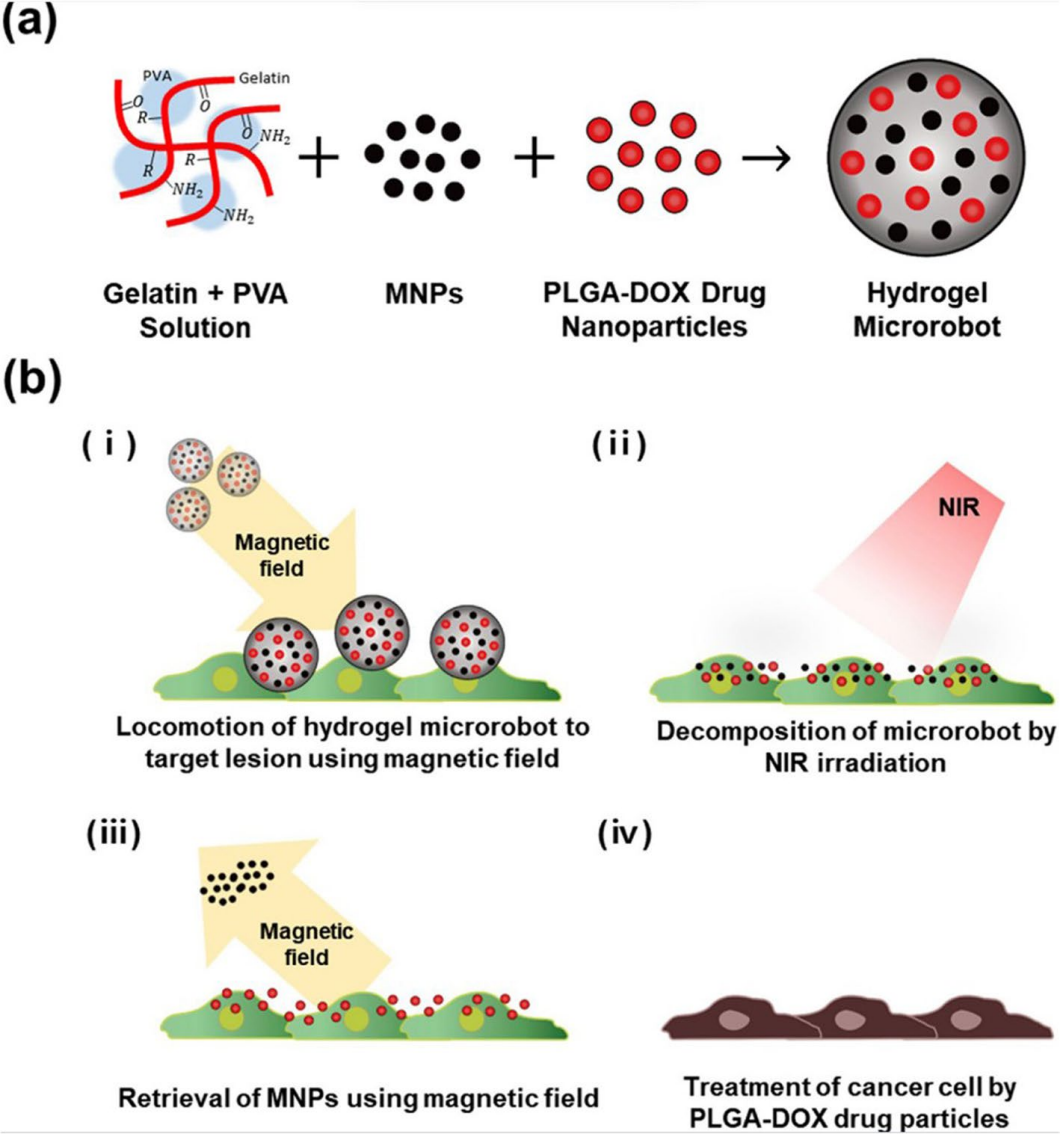
illustration of **A**) fabrication process of Gelatin/PVA solution, PLGA-DOX loaded nanoparticle consisting microbot hydrogel, **B**) Targeting and treatment process of microbot hydrogel with the aid of EMA and NIR for local cancer treatment. Adapted with permission from [383]

Cengelli et al. explored the biocompatible interactions of cationic amino ultra-small superparamagnetic iron oxide nanoparticles (USPIONs) with human cells in various dimensional cultures (3D and 2D) by applying biochemical and electron microscopy techniques. The observations revealed that human melanoma cells internalized the amino-SPIONs. The adopted route was clathrin regulated and localized within the lysosome, inducing the stimulation and lowering the expression of the cathepsin D and transferrin receptors in skin fibroblasts undergoing skin melanoma screening [Fig. 12(A), (B), (C)] [250].

Superparamagnetic iron oxide nanoparticles (SPIONs), which contain 5-Flurouracil as a chemotherapeutic medication, were created by Raviraj et al. On topically treated hairless mice with skin cancer, the produced SPIONs and the conventional drug were compared for skin penetration by fluorescence microscopy and tumor development. The study's findings showed that in immunocompetent mice bearing experimental melanoma, SPIONs containing 5-FU demonstrated increased skin penetration as well as enhanced tumor development retardation when compared to Doxorubicin. Additionally, topical use of nanoparticles increased cytotoxicity and immune cell infiltration, which were further promoted by co-administration of 5-FU, and decreased tumor vascularization independently of 5-FU [384].

##### Gold nanoparticles (AuNPs)

AuNPs are metal nanoparticles with adjustable electrical and optical characteristics that may be employed in a variety of applications such as photovoltaics and medicinal drug delivery systems [15].These particles contain a number of distinctive qualities that set them apart from other approaches currently used and provide very versatile nanoplatforms for biological applications such as the delivery of drugs and genes (Fig. 12(D)) [251]. AuNPs may combine with different other biomolecules without altering their biological characteristics since they are easy to fabricate in the nanosized range, having excellent biocompatibility, and function. The most popular and widely utilized platforms for diagnosing, treating, and keeping track of skin diseases, including skin cancers, are gold nanoparticles (AuNPs) [385].

Limon et al*.* produced gold nanoparticles (GNP) coated with thiolates that were highly solubilized in water. By utilizing confocal fluorescence microscopy, the fluorophore uptake and internalization coated AuNPs in keratinocytes of humans were embellished. The operationalized nanoparticles hinder the actions of intracellular KLK5 and HaCaT and minimized the IL-8 secretion which is stimulated by TLR-2 ligands. The present analysis reveals that GNPs have the capability to deliver drugs towards the novel intracellular regions and antibodies which are used as a therapeutic agent to treat skin cancer and other diseases, including Rosacea [386].

The Nimotuzumab-containing gold nanoparticles (AuNPs-NmAb) were created by Mohammad Anisuzzaman et al. by a coupling reaction. After being PEGylated functionalized, the AuNPs-NmAb were found to be stable. Even at high concentrations of both, the AuNPs and NmAb alone do not have the same anti-cancer effect as the AuNPs-NmAb. Due to the varying levels of EGFR expression in the two cell lines (A431 and A549), the AuNPs-NmAb showed greater cytotoxicity in skin cancer cell lines (A431) than in lung cancer cell lines (A549). AuNPs-NmAb and NmAb were discovered to have IC50 values of 142.7 and 163.6 g/mL on A431 and 561.3 and 1082.0 g/mL on A549. This is anticipated to significantly lower cell survivability. Overall, the study emphasizes the distinct therapeutic potential of AuNP-NmAb in the treatment of malignancies that specifically target the EGFR + receptor [387].

##### Titanium dioxide

It is widely accepted that titanium dioxide (TiO_2_) nanoparticles, which are used as inorganic physical sun blockers in sunscreens, can prevent skin malignancies caused due to sun exposure.

The combination of these particles ensures wide UV protection since zinc oxide (ZnO) and TiO_2_ are more effective in the UVA spectrum and UVB range, respectively [388].

TiO_2_ nanoparticles that have undergone photocatalysis may also have antitumor characteristics, which makes them particularly interesting for conventional anticancer treatments. To improve anticancer characteristics and eliminate in situ UV light introduction in human treatment, alcohol, amine, and carboxylic acid groups may be functionalized onto the surface of TiO_2_ nanoparticles. Functionalized TiO_2_ nanoparticles cause membrane rupture and consequent cell apoptosis in melanoma-derived cultured cells [389].

Without exposure to light, TiO_2_ NPs are nontoxic and stable for a long period within the body. Due to the inability to directly illuminate the tissue with UV or visible light in order to stimulate TiO_2_ nanoparticles, PDT has significant limits in treating tumors that are far from the skin, although it may be very effective in treating skin cancer. TiO_2_ nanoparticles responded well to this technique, which allows for the greatest tissue penetration in the near-infrared (NIR) region (700–1000 nm) [390]. The main reason titanium dioxide was used to treat skin cancer was that it produces reactive oxygen species (ROS) when exposed to light or UV radiation, however, it lacks selectivity and also damages nearby healthy cells. In order to specifically target cutaneous carcinoma, which has an overexpression of integrin receptors (αvβ3), Avraham Dayan et al. produced titanium dioxide nanoparticles (TiO_2_) coupled with Arg-Gly-Asp (RGD). The dihydrolipoamide dehydrogenase (DLDH), a mitochondrial enzyme, creates a strong and precise coordinative connection with TiO_2_, according to the study. Therefore, the modification of RGD moieties with DLDH results in the formation of a molecular bridge between photosensitive TiO_2_ and cancer cells that express integrin. The hybrid conjugate (TiO_2_-DLDH^RGD^) that is thus formed under UVA radiation produces controlled ROS, with Anatase TiO2 being the most photosensitive metastable form of TiO2. The formulation showed increased cytotoxicity after cell line experiments on B16F10 (mouse melanoma cells expressing the αvβ3 integrin), but not on normal cells missing the HEK293 (integrin). Overall, the research points to the viability of TiO_2_ with PDT, which exhibited zero toxicity in the absence of UV light [391].

##### Zinc Oxide Nanoparticle

For a variety of tumor forms, ZnO nanoparticles demonstrated their anticancer potential both in vitro and in vivo [392]. Recent studies have shown that the use of ZnO nanoparticles sensitizes tumor cells by increasing both mitotic (linked to cytogenetic damage) and interphase (apoptosis) death. This is accurate even though the intimate antitumor effect of ZnO nanoparticles varies in different tumor cells and is still not fully understood. ZnO nanoparticles trigger the development of micronuclei in cells, which causes them to act as genotoxic substances. Studies reveal that intracellular generation of ROS considerably rises in melanoma cancer cells following treatment with varied ZnO nanoparticle doses, suggesting that ZnO nanoparticles may be used in a variety of skin-related applications. Their antitumor action includes the induction of apoptosis (Fig. 12(E), (F) (G), (H)). According to research, treating tumor cells with these nanoparticles modulates the expression of critical apoptotic genes such as the tumor suppressor gene p53, bax, caspase-3, and Bcl-2 [252].

Recently, these were used to create therapeutic cancer vaccines, which are developing as part of a unique anticancer method that uses particular antigens to activate and modify the antitumor immune response through dendritic cells [393]. For this concept, iron oxide (Fe_3_O_4_)-ZnO core–shell nanoparticles with ZnO-binding peptides were created to transport tumor antigens in dendritic cells. In mice with generated subcutaneous tumors, injection of dendritic cells with the (Fe_3_O_4_)-ZnO core–shell nanoparticles-antigen combination resulted in delayed tumor development and improved survival rates. Furthermore, one shown benefit of utilizing ZnO nanoparticles in cancer treatment is a decrease in active medication dosage, with a corresponding reduction in side effects associated with chemotherapy [390]. Dhanya George et al. formulated the quercetin-loaded chitosan-cellulose hydrogel comprising green zinc oxide nanoparticles as a biocompatible and controlled release of anticancer drug. Dialdehyde cellulose was developed from sugarcane bagasse cellulose followed by crosslinking with chitosan hydrogen. The green zinc oxide nanoparticles were embedded in the previously formed hydrogel. After optimization by Taguchi design the drug loading of quercetin in ZnO loaded hydrogel was found by 91.36% compared to hydrogel without ZnO NPs. This formed Hydrogel showed maximum drug release at pH 5 which is the ideal condition in cancer. Also, the anticancer activity was established against the human skin cancer cell lines (A431) with enhanced cell uptake and decreased IC_50_ values [394].

##### Cerium Oxide Nanoparticle

Cerium oxide nanoparticles (CNPs) are a relatively new method in the treatment of cancer, with the "smart" ability to selectively trigger cellular apoptosis in irradiated cancer cells, making them appropriate for radiation therapy. A distinguishing feature of these nanoparticles is that they specifically target cancer cells while preserving surrounding tissue from radiation-induced damage and oxidative stress, functioning as both radio-protecting and radio-sensitizing agents at the same time [395]. Below mentioned Fig. 12(I) and (J) represents transmission electron microscopy (TEM) and field emission scanning electron microscopy (FESEM) image of cerium oxide nanoparticles.

The fact that these nanoparticles specifically induce oxidative stress and apoptosis in irradiated cancer cells is explained by the suppression of their intrinsic catalase-like activity that happens in acidic conditions, at a pH of 4.3. Another putative reason for CNPs' differential toxicity is their superoxide dismutase (SOD)-like activity. This enzyme functions as a radio-sensitizing agent, amplifying the DNA damage response, and it is thought that CNPs may also operate as radio-sensitizing agents through another biological mechanism that regulates the response to DNA damage. Although the impact might occur in a variety of malignancies, it was reported and extensively studied in melanoma cells [396]. Tannic acid-containing Cerium oxide nanoparticles (TA-CNPs) were made by Ragina G. Dare et al., and their photoprotective effect against L929 fibroblasts was examined. The CNPs and TA-CNPs are generated with a particle size of 5–10 nm, a zeta potential of + 23 and -19 mV, and superoxide dismutase activities of 3724 and 2021 units/mg. The goal of the study was to minimize UVB oxidative stress, which was not seen in TA-CNPs, as well as the cytotoxicity linked to dose-dependent free TA. Additionally, TA-CNPs' photoprotective activity was enhanced in comparison to that of CNPs, providing higher protection against the endogenous antioxidant defense (CAT and GSH), ROS scavenging activity, and lipid/DNA prevention. The TA-CNPs increased cell proliferation lowered TGF- and COX-2 expression, and decreased MMP-1 expression (the primary enzyme for collagen degradation), all of which led to a reduction in the inflammatory reactions linked to UVB radiation. Because of this, TA-CNPs are an effective nanoparticulate strategy for reducing photoaging, reducing UVB-related inflammation, and preventing photodamage [397].

##### Silver Nanoparticle

Light may be efficiently absorbed and scattered by silver nanoparticles (AgNPs). Nano-biosensor is a novel combinatorial nanotechnology and optical biosensor that is locating use in environmental monitoring, medical, drug screening, and food safety [398].

The nano biosensor based on localized surface plasmon resonance (LSPR) is a newer kind of optical biosensor technology created by stimulation when the incoming photon frequency is resonant with the collective oscillation of the conduction electrons. Recently, an LSPR biosensor based on AgNPs was developed for the measurement of p53 protein levels in HNSCC patients [399]. Agglomeration, dissolution rate, surface charge and coating, Shape, and LSPR are all physicochemical features of AgNPs. The surface charge and size of AgNPs affect their cytotoxicity. Because of their bigger surface area, smaller particles are more hazardous. Spherical AgNPs, nanowires, nanorods, nanoplates, and nanotubes are common silver nanostructures utilized in the biomedical field. The interaction of NP with diverse biomolecules at the target location is regulated by the surface charges of AgNP coatings. Formulation of drugs in the nano range can control the penetration of the drug into the skin based on the design and physicochemical qualities of the component [44]. Numerous types of research have emphasized the chemical as well as physical features of cubosomes in the development of anticancer drugs. The therapeutic application of nanodiamonds has been prompted by their potentially favorable qualities for labeling and drug administration, such as inertness, tiny size, and surface structure [400]. Silver nanoparticles were formulated by Nádia S. V. Capanema et al. and embedded in a carboxymethyl cellulose hydrogel system with doxorubicin (AgNP@CMC-DOX) for the treatment of melanoma skin cancer and antibacterial activities. Electrostatic interactions between the AgNP@CMC (core–shell nanostructure) and DOX were used to create a colloidal nanostructure. The chemical crosslinking of the resulting nanostructure using citric acid was then carried out. The silver nanoparticles discovered have a size range of 10 nm. The data shows that the hybrid hydrogel releases DOX across ethnic boundaries using tailored kinetics and kills melanoma skin cancer, demonstrating the synergistic effect of the hybrid hydrogel and AgNPs [401].

#### Polymeric Nano-micelles

Nano-micelles have been utilized to deliver drugs to the skin. Because they are only deposited in hair follicles, these nano-carriers have minimal limitations. They may not penetrate deep into the skin layer [402]. Kahraman et al. revealed that the nano-micelles containing terpenes face a lot of difficulties as well as challenges to find potential applications for skin delivery. The accumulation of the drug, as well as its permeation, was analyzed by the tape-stripping procedure in the skin. The obtained results reveal that nano-micelles containing terpinolene may become the reason for the accumulation of highly concentrated drugs in the striped skin as compared to the commercial product and micelles without terpene. The nano micelles containing terpinolene can be a more beneficial approach for drug delivery [403].

Wang et al. fabricated the polymer-based nano-carriers for transdermal administration of siRNA that target skin melanoma. The effectiveness of encapsulated siRNA in preventing the spread of skin cancer is reproduced. According to this research, cationic micelles work well as gene-delivery nanoplatforms for treating melanoma [404].

Xu et al. designed paclitaxel-loaded micelles, and hydrogel was formed while loading these nanoparticles for the treatment of cutaneous melanoma. The following formulation conducted a skin penetration and retention trial. The B16 melanoma cell lines under study exhibited more cellular absorption, and in vivo tests on the B16 cells revealed preferred anticancer activity compared to free taxol [405]. For the effective treatment of skin cancer, Lamch et al. created polymeric micelles loaded with zinc (II) phthalocyanine driven by the folate moiety. In the study, A block polymer called methoxypoly(ethylene oxide)-b-poly(L-lactide) (mPEG-b-PLLA) and its derivatives with folate attached to the PEG chain (FA-PEG-b-PLLA) were used as biocompatible micelles to incorporate the folate-directed Zinc (II) phthalocyanine (ZnPc), a well-known second-generation photosensitizer. After DLS, it was found that the synthesized polymeric micelles had particles which measured ~ 150 nm in size and a low PDI. The mPEG-b-PLLA resulted in improved cellular uptake and greater functionalization in vitro. When the polymeric micelles were functionalized with FA, the loading of ZnPc increased, increasing the ZnPc dose at the cancer cells that are overexpressing FA receptor. This makes polymeric micelles, together with PDT techniques, a promising nanocarrier in the treatment of skin cancer [406].

#### Polymeric Nanoparticles

Polymeric nanoparticles are extremely important for skin application due to their enhanced stability and controlled release and permeation via polymeric matrix to penetrate the skin. These polymeric nanoparticles conserve the design for a prolonged period of time. Natural polymeric NPs including chitosan, gelatin, albumin, and alginate are commonly employed for topical skin administration and targeting skin melanoma. Matsumura et al. (2004) were the first to synthesize polymeric nanocarriers, PEG-poly (amino acid) micelles that accumulated in solid tumor tissues and were subsequently used in clinical studies [407].

According to Dias et al., the anti-neoplastic agent IMQ is used to treat skin cancer. The increased frequency of local and systemic adverse side effects associated with lower skin penetration, on the other hand, may jeopardize therapeutic potential. The objective of this research was to examine the anti-angiogenic and anti-tumoral effectiveness of polymeric nanoparticles encapsulated with IMQ. In chicken chorioallantoic embryos, the anti-angiogenic performance of the produced combination was examined. In a mouse carcinogenesis model, its chemo-preventive potential was also examined. It was established that the proposed delivery method can be utilized to treat vascular developmental issues, as well as to increase skin efficiency and penetration of less soluble medications used to treat skin disorders such as skin melanoma [249].

Hasanovic et al. demonstrated that encapsulating acyclovir (anti-viral medication) into chitosan-TPP NPs resulted in increased drug incorporation, and minimal photo-degradation, with increased drug permeation through porcine skin [408]. Zhao et al. developed a cancer cell membrane camouflaged Dacarbazine loaded porous silica nanoparticles for the treatment of melanoma. The study showed that the cancer cell membrane-coated nanoparticles (CCM-CMSN) have great cancer-targeting properties along with an increased circulation half-life and good biocompatibility. This developed cancer cell membrane camouflaged Dacarbazine loaded porous silica nanoparticles (DTIC@CMSN) were given along with the anti-programmed cell death protein 1 antibody (aPD1) immunotherapy for better antitumor efficacy of the chemotherapy. The in-vitro cell line studies and in-vivo animal experiment results demonstrated that the DTIC@CMSN showed remarkable tumor suppression and increased lifespan/ survival due to targeted tumor killing compared to free DTIC after combining with the aPD1 immunotherapy. The in-vivo safety studies also represented that the DTIC@CMSN showed selective tumor accumulation with less systemic toxicity [281].

Chitosan-sodium triphosphate nanoparticles (CS-TPP-NPs) were formulated by Nursyafiqah et al. to target α- and β-arbutin into the skin. the crosslinking of TPP and CS to transform α- and β-arbutin into CS-TPP-NPs. The research points to chitosan nanoparticles as a promising choice for nanocarriers that can carry α- and β-arbutin through the skin for increased efficacy. The study suggests that chitosan nanoparticles could also serve as possible nanocarriers for administering chemotherapy medications to skin malignancies [409].

#### Lipid Nanoparticle

Lipid nanoparticles have been discovered to be among the most biocompatible nanoparticles researched for skin application. Nanoemulsions, liposomes, solid lipid nanoparticles, and nanostructured lipid cargos are among the most widely studied nanoparticles in skin permeation studies. Cubosomes, nanodispersion, and Niosomes are three additional common forms of lipid Nano formulations used for topical or transdermal skin administration that have been described in the literature. These drug delivery methods have been suggested because of their advantages associated such as occlusive properties, variations in release pattern, and enhanced skin perforation with fewer adverse effects [410].

The elastic variety of liposomes may change capillary flow through small skin pores. This lipid formulation is claimed to be capable of penetrating and delivering the drug deeper into the skin's layers. Liposomes encapsulated in dipotassium glycyrrhizinate have been used as an anti-inflammatory medication in the treatment of both acute and chronic dermatitis [411, 412].

Jain et al. discover improved transport of minoxidil encapsulated in neutral liposomes into hair follicles when compared to preceding formulations. Other medications, like finasteride, are used to treat androgenetic alopecia. Anti-androgens, such as RU 68841 myristate encapsulated solid lipid nanoparticles, have been demonstrated to improve active medicinal constituent penetration and delivery. Cyclosporine Liposomal formulations accelerated hair reproduction in rats, demonstrating a promising approach for addressing alopecia areata in humans [413].

By using a high-pressure homogenization technique, Gulin et al. created nano-lipid carriers (NLCs) and solid lipid nanoparticles (SLNs) loaded with 5-FU. The investigation revealed higher anticancer activity of the 5-FU loaded NLCs compared to the free 5-FU towards epidermal carcinoma cells and lower cytotoxicity towards human keratinocyte cells indicating that the NLCs were more effective at delivering the chemotherapeutic drug into the skin than the conventional hydrogel [411].

#### Nano-emulsions (Ne)

Nanoemulsions are stable systems with distinguished rheological characteristics and a clear appearance. It is the most often used delivery technique for the continuous release of API into the deep layer of the skin. The benefits of nano-emulsions over others are that they may effectively reduce transepidermal water loss (TEWL) by retaining the moisture of the skin and are more permeable to APIs. Because of their enhanced solubilization and kinetic stability, they are an advantageous formulation [414, 415].

Squamous cell carcinoma and basal cell carcinoma, two of the most common forms of cancer, were investigated by Severino et al. In similar circumstances, chemotherapeutics revealed nonselective targeting as well as substantial adverse effects. These nanoparticles demonstrated the chemical stability and sustained release of encapsulated medications. Their results suggests improved drug intracellular concentrations with reduced cytotoxicity [416].

Giacone et al. endeavored to develop and evaluate a nano-emulsion for the topical skin absorption of the chemotherapeutic drug piperine. The Chitosan coated nanoemulsion to target melanoma cells was prepared and the cytotoxicity analysis was around 2.8 times greater compared to conventional drug. The research showed the relevant application of chitosan-modified nanoemulsion loaded with piperine to skin diagnosis and management [417].

The cytotoxic agent piplartine (piperlongumine) was encapsulated in a nanoemulsion modified with chitosan or sodium alginate was synthesized by Daniela et al. and it was found that the piplartine nanoemulsion modified with chitosan had a 2.8-fold greater cytotoxicity against melanoma cells than the standard piplartine solution. Overall, the research points to the use of a chitosan modified nanoemulsion containing piplartine as a novel method for improving cytotoxicity and local control of skin cancer [417].

#### Liposomes

Liposomes are small, double-layered phospholipid vesicles with an inner hydrophilic core, comparable to a biological membrane. Liposomes are phospholipid and cholesterol-based nanostructures that have a size range from 50 to 100 nm. Their considerable result as a substitution for delivering the therapeutic constituent to the desired region has been employed to extend the therapeutic profile and minimize the risk of side effects associated with anticancer medications [418].

Liposomes are categorized according to their size and number of layers as multi-, oligo-, or unilamellar. The aqueous center of the liposome encapsulates water-soluble drugs, whereas its lipidic membrane delivers amphiphilic or hydrophobic medications. When injected into the bloodstream, special PEGylated liposomes are engineered to pass through the reticuloendothelial system (RES), minimizing the clearance rate of the active pharmaceutical molecule and enhancing the circulation half-life. Liposomes have excellent qualities for circulation, penetration, and diffusion. A lipidic nanoparticle comparative research for 5-FU NPs was performed, and topical administration of NPs not only improved performance but also reduced the IC50 on the human keratinocyte cells (HaCaT) cell line [419].

Jose et al. examined the effectiveness of co-encapsulated curcumin and anti-STAT3 siRNA against cutaneous melanoma employing cationic-charged liposomes. The entrapment efficiency, zeta potential, and particle size of the liposomes were all examined. Cell line tests on B16F10 viability studies on mouse melanoma cells demonstrated that co-encapsulation of both medicines suppressed cancer cell proliferation when compared to other formulations. Using an iontophoretic approach, positive liposomes encapsulating curcumin may penetrate into the skin to the appropriate depth [420]. In order to target the EGFR receptor, Raquel et al. prepared 5-FU-loaded liposomes using the iontophoresis technique and their in-vitro cell culture examinations result indicate that the cellular uptake of the cetuximab-immunoliposome by EGFR-positive SCC cells was 3.5 times more than that of control liposomes. Additionally, iontophoresis improved the penetration of 5-FU into viable epidermis compared to control liposomes, according to a depth permeation investigation which further lead to better in-vivo results in terms of tumor volume and area compared to conventional formulation. Overall, the research showed that topical administration and iontophoresis increased effectiveness when compared to subcutaneous 5-FU injection and control liposomes by a factor of two [421].

#### Dendrimers

Dendrimers are monodispersed, unimolecular, synthetic polymers that are less than 15 nm in size. They have layered architectures made up of a core which is present at the center, an internal region consisting of repetitive sections, and numerous groups which are terminal parts that determine the three-dimensional dendrimer elemental structures. Because of their desirable features, including multivalency, well-defined size, molecular weight, high degree of branching, monodispersity, number of available internal cavities, and high number of surface functional groups, dendrimers can be prepared for the delivery of nucleic acids, both hydrophobic and hydrophilic drugs, and imaging [422, 423].

The potential of dendrimer targeting ligands to cause the precise targeting and destruction of tumors is shown in several literature sources. They consist of folate and tumor-associated antigen in addition to oligosaccharides, polysaccharides, oligopeptides, and polyunsaturated fatty acids [424]. Although, it is still challenging to get drugs connected to dendrimers with a controlled release. A novel class of molecules known as dendronized polymers, which are linear polymers that carry dendrons at each repeat unit, has been created as a result of recent advances in polymer and dendrimer chemistry. These molecules benefit from improved drug delivery due to their prolonged circulation period. Since no covalent bonds are needed, this delivery method may carry several molecules of an anticancer medication without appreciably altering the antibody's capacity to target tumors. Additionally, they have undergone surface functionalization with receptor agonists and antagonists for tumor targeting. They have also been modified to transport gadolinium atoms for MRI of malignancies. Recently, a review on the use of carbon nanotubes in the detection and treatment of melanoma was published [425].

#### Magnetic Nanoparticle

In a B16F1 xenograft mouse model, Sato et al. developed a magnetite nanoparticle by conjugating N-propionyl-cysteinyl phenol with magnetite [426]. Electron microscopy revealed that this particle exclusively appeared in melanoma cells. It was discovered that melanoma cells were degraded when an external alternating magnetic field was used to raise the temperature of the tumor to 43 °C. The nanoparticle outperformed magnetite alone by a factor of 1.7 to 5.4. Curcumin was shown to improve the effectiveness of magnetite nanoparticles in recent research [182]. Dong-in et al. fabricated the microbots composed of gelatin or Polyvinyl alcohol (PVA) hydrogel and the Magnetic nanoparticles and PLGA (Poly-Lactic-Co-glycolic acid) previously matrixed with Doxorubicin (PLGA-DOX particles). The targeting of these microbots to the tumor site is facilitated by the external Near-IR integrated Electromagnetic Actuation (EMA). Firstly, with the aid of the EMA system, the microbots are targeted to the tumor site followed by disassembling of magnetic nanoparticles with the help of EMA. After the removal of magnetic particles from Microbots hydrogel only PLGA-DOX remained at the target site which then release the DOX into the target site in a controlled manner [383].

In one of many research studies with the similar nanoparticles, Reeju et al., developed iron oxide nanoparticles-loaded hydrogel for the topical photothermal therapy of skin cancer. The results of the study exhibited that gel had the desired viscosity which was favourable for the application on the skin. The findings of in vitro and in vivo demonstrated the evidence about the effective applicability of these s against skin cancer [427]. These magnetic nanoparticles have tremendous potential in different cancers including non-melanoma skin cancer, however, there is limited literature which endorses the application of these nanoparticles.

#### Nanostructured lipid carriers (NLCs)

NLCs are a better alternative against Solid-lipid nanoparticles (SLNs) for topical preparation because of numerous benefits due to the utilization of biodegradable lipids which ensure low systemic toxicity and low cytotoxicity as well. Small sizes of NLC particles could enhance the close contact of formulation with stratum corneum which amplifies the drug to flux through the skin and due to the presence of solid lipid into it, it also enhances the controlled release of API from the carriers [63–65]. Two types of lipid nanoparticles i.e. SLN and NLC were differentiated according to the constituents and internal structure, in which SLN (40-1000 nm diametric range) can be stipulated as the 1^st^ generation of lipid particle matrix after the emulsion which is performing as an alternative of emulsion system; having an amalgam of solid lipid (0.1%-30%w/w) which is stable and solid at the room as well as body temperature [257, 258]. SLN contains numerous limitations likely, drug loading ability, drug release tendency, and expulsion of the drug throughout the storage period which has to be overcome by NLC; which has respective convenience over SLN as NLC comprises of liquid lipid in addition to solid lipid which provides much stability and flexibility to nanoparticles, provide higher drug loading capacity, etc. [259]. However, the presently marketed formulating gels for skin treatment have various limitations which could be enhanced by integrating numerous upgradations in it; hence it needed serious attempts to do some progression of skin cancer therapeutics by evolving it into combinatorial form and doing some modifications in it [3, 260, 261].

In another study Kashif et al., developed a combinatorial chemotherapy for non-melanoma skin cancer. They codelivered 5-Flurouracil with a polyphenolic compound (resveratrol). The findings of the study demonstrate that the size and zeta potential of developed nanocarrier was within the required range of stable formulation. Interestingly, they showed that the successful encapsulation of these two therapeutics in this kind of nanovehicle provides a sustained release of APIs after the application topically. Further findings of confocal microscopy revealed that the developed nanocarrier and its loading into the gel can go into the dermal layer of the skin. In the extension of this study, they validated their proposal in vitro and in vivo and demonstrated that antiproliferative effects [428]. In another study from the same laboratory, Babar et al., developed silymarin loaded nanostructured lipid carrier for the treatment of skin cancer. The experiments were conducted on B16 melanoma cells and albino mice to confirm the chemotherapeutic efficacy of developed drug-loaded nanocarrier. The results exhibited that the tumor volume and level of IL-1α and TNF-α were significantly (*p* < 0.05) reduced after the application of silymarin-NLC gel as compared conventional gel. Moreover, similar study conducted by Nazeer et al., they investigated the role of cannabidiol and 5-FU in combination after loading in NLCs for the non-melanoma skin cancer. The findings demonstrated the significant potential against cancer cells in vitro and in vivo [3, 8]. Therefore, based on the findings it could be said that drug loaded nano-formulation shows better therapeutic efficacy compared to conventional formulations.

## Article summary

This article summarizes the different types of approaches for the prevention, management and treatment of skin cancer; a) Physical approaches, b) Dermal chemotherapy, c) combination of physical therapy and chemotherapy (Physico-chemical combination therapy), d) Dug-Drug combination therapies, e) Herbal drugs for the skin cancer management, f) nanotechnological approaches for the drug delivery to the skin cancer which are well tabulated in Table 7.

**Table 7:** Summarizes the different types of approaches for the prevention, management and treatment of skin cancer; a) Physical approaches, b) Dermal chemotherapy, c) combination of physical therapy and chemotherapy (Physico-chemical combination therapy), d) Dug-Drug combination therapies, e) Herbal drugs for the skin cancer management, f) nanotechnological approaches for the drug delivery to the skin cancer

**a: Physical methods used for the treatment of skin cancer**		
**Sr. No**	**Type of approach**	**Inference**
1	Surgery	
	a. Simple excision	Areas associated with tumours are completely cut out and removed, along with some of the body's normal cells or tissues, after which the dermatopathologist sutures the wound
	b. Mohs micrographic surgery	It is utilized more frequently for big area tumours, which combine the excision of tumorous tissue with microscopic analysis during the surgical procedure
	c. Electrodessication and Curette (ED&C)	The health specialist uses a curette, an instrument with sharp edges and a small scoop, to remove the tumor after first numbing the area by applying local anesthetic. To stop any further bleeding, they use a probe that uses electricity or electric current
2	Photodynamic therapy	When a specific wavelength of light is shone upon the affected area after a topical drug has been applied, the area becomes further stimulated. The impact of photochemical reactions on biological tissues is the key element of photodynamic therapy (PDT), a method of treating malignancies, including cancer. These processes' starter is light energy
3	Immunotherapy	Antigen–antibody interactions are the basis of the innovative cancer treatment method known as immunotherapy. Interferon is a key component of immunotherapy for the treatment of cancer and acts on target cells by tying up with receptors on those cells. Interferons have an antiproliferative effect on cancer cells and aid the immune system in combating substances that cause cancer
4	Targeted therapy	Targeted therapy drugs stop the growth of melanoma cells that have mutant serine/threonine-protein kinase B-Raf (BRAF), mitogen-activated protein kinase (MEK), or tyrosine-protein kinase Kit (C-KIT) genes. Only these mutant genes are subject to targeted therapy; however, not all melanoma cells carry these mutations
5	Chemical peel	Trichloroacetic acid (TCA) must be applied directly to malignant lesions during a chemical peel, which results in the top layer of skin being removed. It is mostly used for skin lesions that are precancerous
6	Radiotherapy	A successful and flexible non-surgical (or medical) method for tissue preservation is radiotherapy. For those for whom excision may not be viable (medically or technically inoperable) or may be seen as less ideal (e.g., cosmetic consequence), radiotherapy is an excellent alternative
**b: Dermal Chemotherapy Approaches for skin cancer treatment**		
**Sr. No**	**Type of approach**	**Inference**
Dermal Chemotherapy		
1	Topical chemotherapy	Dermal drug delivery is a general phrase used to describe the topical or transdermal application of dosage forms to the skin. In some cases, topical treatment may be more successful, particularly when the patient declines surgical intervention or when surgery is not an option
	a. 5- Fluorouracil (5-FU)	The pyrimidine analogue 5-FU is an antineoplastic antimetabolite that binds to thymidylate synthase with the help of the co-factor 5,10-methylene tetrahydrofolate. Inhibition of thymidine synthesis, impaired DNA replication, and consequent induction of apoptosis ensue from this. A typical topical treatment for superficial basal cell carcinoma (sBCC) is a 5% 5-FU cream or solution
2	Topical immunotherapy	
	a. Imiquimod (IMQ)	Low-molecular-weight IMQ is a synthetic substance. It belongs to the imidazoquinolinone family. Off-label, nBCC uses 5% cream of IMQ. To combat sBCC, it is a permitted medication, though
3	Other drug therapies	
	a. Retinoids	Retinoids can modify immunological response, keratinocyte differentiation, apoptosis, tumor cell proliferation, or a combination of these to exert their chemo-preventive effects. A clinical study that used the retinoic acid derivative tazarotene to treat BBCs proved successful. A clinical trial looked into the best way to treat sBCC or nBCC by applying 0.1% tazarotene gel once daily for up to 8 months
	b. Ingenol mebutate (IM)	One of the materials recovered from the sap of the Euphorbia peplus plant is IM. By encouraging the production of anti-tumor antibodies and proinflammatory cytokines, IM promotes cellular cytotoxicity and inhibits recurrence. topical IM 0.05% gel in BCC, which characterizes the inflammatory response
**c: Physico-chemical Combination therapies**		
**Sr. No**	**Type of approach**	**Inference**
1	Pembrolizumab + Postoperative radiotherapy	The study found no dose-related impact and a progression-free survival rate of 94.4%. 5.56% of the patients had some mild toxicities, such as anemia, rash, fever, edema, etc
2	liposomal gold nanoparticles encapsulating curcumin + Photothermal treatment	The absorption of gold nanoparticles with curcumin encapsulation was greatly enhanced. Because of the presence of curcumin, the group treated with curcumin gold nanoparticles shown increased cytotoxicity to cancer cells after laser irradiation
3	Titanium-dioxide-nanoparticle–gold-nanocluster–graphene + PDT	Treatment with TAG nanocomposites intravenously or intratumorally can significantly affect the clinical tumor tissue of mice with B16F1 tumor xenografts and decrease cancer progression
4	Photodynamic therapy (PDT) + Photothermal therapy (PTT)	Complete cell/tumor eradication has been observed in one as well as other in vivo as well as in vitro, suggesting that the combination PDT/PPTT activity generated by AuNRs/MoS2-ICG are substantially more effective than either one of synergistic PPTT or PDT alone
5	5-FU loaded immunoliposome + Iontophoresis	In comparison to identical therapy utilizing control liposomes, the study showed that 5-FU penetration into viable epidermis by iontophoresis of immunoliposomes increased that penetration
	Curcumin and Topotecan nanocapsules + Ultrasound	The effect on tumor growth was shown to be 3.5 and 14.8 times greater when ultrasound was employed in conjunction with this produced formulation as opposed to when ultrasound was not used and when only a physical mixing of medications was used, respectively
**d: Drug Combination therapy for the treatment of skin cancer**		
**Sr. No**	**Type of approach**	**Inference**
1	Ipilimumab + Interleukin-2 (IL-2)	A higher percentage of complete responses, a sustained survival without cancer progression, or an improved response rate are all preferred outcomes
2	Iron oxide magnetic nanoparticles (NPs) + TLR agonists	The vaccines were more effective on B16F10 melanoma cells that were aggressive. Additionally, the researchers were able to track the vaccine's path from the injection site to the lymph nodes and tumor using magnetic resonance and nuclear imaging
3	Ipilimumab + T-VEC	When employed in combination, a 38.8% Objective Response Rate (ORR) was attained. However, when ipilimumab was administered alone, the ORR was just 18.0%
4	Toll-like receptor-9 (TLR9) + CMP-001	By reducing the chronic inflammation brought on by systemic immunotherapies, TLR9 agonists may be used in combination therapies to combat immunological depletion and restrict metastases
5	Trametinib + Atezolizumab	Trametinib and atezolizumab together against BRAF-wild type melanoma demonstrated encouraging results in patients
6	Nivolumab + Ipilimumab	Nivolumab and ipilimumab in combination showed persistent, improved clinical outcomes than monotherapy in trial participants with advanced melanoma
7	5-FU topical therapy + Cetuximab	enhanced efficiency of topical 5-FU treatment for SCC. After incubating with A431 cells for 120 h, cetuximab had a high cytotoxic effect, while immunoliposomes containing cetuximab and 5-FU indicated synergism
8	Rapamycin + PHT-427	In comparison to vehicle controls, the combination led to a substantial decrease in tumor multiplicity. Combining topical Akt inhibitors (PHT-427) with mTOR inhibitors (rapamycin) may be a successful chemoprevention method for patients who have a high risk of developing cutaneous SCC
**e: Herbal drugs used for prevention and management of skin cancer**		
**Sr. No**	**Type of approach**	**Inference**
1	Quercetin	Flavanol quercetin can be identified by the presence of -OH groups at positions 3, 5, 7, and 3' and 4' of the flavanol framework. The main contributors to quercetin's anti-cancer effect are its anti-oxidant and anti-inflammatory capabilities. In murine melanoma cells (B16-BL6), quercetin decreased Bcl-2 expression and activity and increased the activity and potential of caspase-3, which caused the cells to die
2	Kaempferol	Kaempferol-rich meals have been linked to a lower incidence of pancreatic, gastric, lung, ovarian, and other cancers as well as cardiovascular disease. Kaempferol has been shown to disrupt the choroidal melanoma cell cycle's G2/M phase
3	Epigallocatechin-3-gallate	The primary sources of epigallocatechin-3-gallate (EGCG), also known as flavan-3-ols, are tea, red wine, strawberry, and cocoa goods. EGCG can stop the cell cycle and induce apoptosis in melanoma cells (A374 and Hs-294 T). It can have a number of effects, such as the induction of tumor-suppressor proteins (p16INK4a, p21WAF1/CIP1, and p2KP1), upregulation of the pro-apoptosis protein Bcl-2 associated X protein (Bax), activation of caspases-3, -7, and -9, and downregulation of proteins that inhibit apoptosis or promote cell survival
4	Apigenin	The flavonoid apigenin, also known chemically as 4',5,7, -trihydroxy flavone, is thought to prevent UV-induced skin cancer by triggering the AMPK pathway (AMP-activated protein kinase), which in turn suppresses baseline mTOR activity in keratinocytes grown in vitro
5	Daidzein	Daidzein, commonly known as soy isoflavone, was thought to have anticancer properties by preventing CDK2 (Cyclin dependent kinase) from activating during the G1 phase of the cell cycle
6	β-Carotene	Carrots, kale, pepper, spinach, pumpkin, sweet potatoes, and cantaloupe are some of the main sources. The inhibition of Bcl-2, p53, and caspase-3 in murine melanoma cells may be the mode of action of -carotene, which subsequently induces apoptosis and potency of -carotene upon tumour specific angiogenesis, which affects tumor growth
7	Resveratrol	Resveratrol, a naturally occurring group-A stilbene phytoalexin antioxidant, has been demonstrated to inhibit the lipopolysaccharide-induced epithelial to mesenchymal transition, most likely by modulating NF-B signaling as a result of its anti-metastatic effects
8	Curcumin	Curcumin's ability to inhibit tumor growth is aided by three distinct mechanisms: (i) Upregulation of tumor suppressor genes (like p53) and downregulation of anti-apoptotic proteins causes apoptosis to be induced in tumor cells. (ii) Matrix metalloproteinase (MMP) downregulation, which inhibits tumor invasion; and (iii) the anti-inflammatory action, which boosts its effectiveness against tumors
9	Sulforaphane	It can be found in cruciferous vegetables like cauliflower, radish, cabbage, and broccoli. It has been shown that sulforaphane possesses anticancer properties, including the potential to cause apoptosis, limit cell proliferation, and stop metastasis. Sulforaphane induces apoptosis by triggering the p53 protein, caspase 3, caspase 9, the Bax gene, and caspase 9
10	Hypericum perforatum	John's Wort, often known as St. Hypericum perforatum, is a penanthroperylene quinine with photosensitive properties. Due to its concentration and low dosage dependent photo-sensitizing properties, Hypericin is an excellent candidate to be employed in photodynamic therapy for skin cancer. In melanoma, the UVA activated hypericin produced cell demising by the necrosis and apoptosis pathways
11	Withania somnifera	Due to Withaferin A's capacity to down regulate Bcl-2 and trigger the generation of Reactive Oxygen Species (ROS), Withania Somnifera, also known as Ashwagandha or Indian ginseng, causes Melanoma cells to undergo apoptosis when administered alone
12	Melaleuca alternifolia	It is also known as tea tree oil, terpinen-4-ol, or tea tree oil, and studies in vitro have shown that it can slow the growth of melanoma cells by inducing apoptosis, cell cycle arrest, necrosis, and preventing cell proliferation at low dosages while being safe for normal fibroblast cells
13	Rosmarinus officinalis	The main component in R. officinalis extract is carnosol, a phenolic diterpene. Carnosol prevented metastatic murine melanoma cells from migrating into the basement membrane, and this result was linked to the suppression of MMP-9
14	Aloe Species	Emodin exhibits an anti-proliferation impact that is time-dependent and can stop MMP-9's anti-melanoma action. Aloe-emodin treatment has anti-metastatic effects by inhibiting murine melanoma cell adhesion, invasion, migration, and aggregation
15	Ingenol Mebutate	It is a modulator of eight PKC (Protein Kinase C isoenzyme) isoforms that are involved in signaling pathways such as cell angiogenesis, invasion, survival/cell death (survival/autophagy), and cell proliferation. It also functions as either a tumor suppressor or an oncogene
16	Zingiber officinale	The p38 MAP kinase-NF-B signaling pathway was normally blocked to prevent TPA's induction of COX-2 in mice, which is what caused the anticancer activity
17	Cannabinoids	In melanoma cells, the phytocannabinoid D9- Tetrahydrocannabinol (THC) induces autophagy, leads to apoptosis, and reduces cell viability. These effects were strengthened by the addition of cannabidiol, and they were demonstrated in a mouse model of melanoma with xenografts
18	Retinoids	The nucleic receptor (NR) of the Retinoic Acid Receptors (RAR) subfamily, including as RAR, RAR, and RAR, is where retinoids are expected to work
**f: Nanotechnological approaches to tackle skin cancer**		
**Sr. No**	**Type**	**Inference**
1	Nano-biosensors	Indicators of skin diseases are frequently identified using biosensor or nano-biosensor technologies. Nano-sensors are employed as a perceptual aid in the physical evidence of a living creature. Building immunosensors that are beneficial for cancer diagnosis requires the use of antibodies that are electrochemically transducer-anchored
2	Quantum Dots	These are nanocrystals with colloidal fluorescent semiconductors that range in size from 2 to 10 nm. Etoposide and methotrexate were grafted onto Fe3O4-Ag2O quantum dots that were adorned on cellulose fibers. These created Fe3O4-Ag2O quantum dots displayed ferromagnetic properties and released etoposide and methotrexate at rates of 78.94% and 63.84%, respectively
3	Nanotubes	Coaxial graphite sheets are twisted into cylindrical or barrel shapes to form carbon nanotubes, a type of fullerene. By measuring cell survival, free radical generation, and the buildup of metabolites associated with peroxide, researchers were able to explore the impact of extended single-wall carbon nanotube (SWCNT) exposure on cellular toxicity and oxidative stress
4	Iron Oxide Nanoparticles	Because of its improved drug encapsulating capacity, strong magnetic responsiveness, and improved targeted delivery efficiency, the superparamagnetic iron-oxide NPs (SPION) have been investigated for drug targeting and other biological applications
5	Gold nanoparticle	Gold nanoparticles (AuNPs) are the most commonly used and highly accepted platforms for identifying, treating, and monitoring skin conditions, including skin cancer
6	Titanium Dioxide	They serve as inorganic physical sun blockers in sunscreens and help stop skin cancer from being brought on by exposure to the sun. The potential for antitumor properties in photocatalyzed TiO2 nanoparticles makes them especially intriguing for use in traditional anticancer therapies
7	Zinc Oxide Nanoparticle	By enhancing both mitotic (related to cytogenetic damage) and interphase (apoptosis) death, the application of ZnO nanoparticles sensitizes tumor cells. Studies show that melanoma cancer cells' intracellular ROS production significantly increases after being treated with different doses of ZnO nanoparticles, indicating that ZnO nanoparticles may be employed in a range of skin-related applications
8	Cerium Oxide Nanoparticle	Cerium oxide nanoparticles (CNPs) are a relatively new approach to treating cancer. They are suitable for radiation therapy because of their "smart" ability to selectively trigger cellular apoptosis in radio-exposed cancer cells
9	Silver Nanoparticle	AgNPs' cytotoxicity is influenced by their surface charge and size. Smaller particles pose a greater threat because of their greater surface area
10	Polymeric Nano-micelles	Drugs have been applied to the skin using nano-micelles. polymer-based nano-carriers for siRNA delivery to the skin in order to treat melanoma. The ability of encapsulated siRNA to stop skin cancer from spreading is replicated. This study shows that cationic micelles are efficient gene delivery nanoplatforms for melanoma therapy
11	Polymeric Nanoparticles	Due to their improved stability, controlled release, and capacity to permeate the skin through a polymeric matrix, polymeric nanoparticles are crucial when applied to the skin. Cancer cell membrane coated nanoparticles (CCM-CMSN), which also have an extended circulation half-life and outstanding biocompatibility, offer excellent cancer targeting abilities
12	Lipid Nanoparticle	The most biocompatible nanoparticles studied for skin application are lipid nanoparticles, it has been found. Lipid formulation is said to have the ability to penetrate the skin and deliver the medication deeper into its layers
13	Nano-emulsions	Nano emulsions are dependable systems with distinct rheological traits and a transparent appearance. By keeping skin moisture in place and being more permeable to APIs, nano-emulsions have an advantage over other types of emulsions in reducing trans epidermal water loss (TEWL)
14	Liposomes	Small, double-layered phospholipid vesicles called liposomes, which resemble biological membranes in their inner hydrophilic core, are composed of phospholipids. When administered intravenously, unique PEGylated liposomes are designed to pass through the reticuloendothelial system (RES), reducing the rate at which the active pharmaceutical molecule is cleared and lengthening the circulation half-life
15	Dendrimers	Dendrimers are synthetic monodisperse, unimolecular polymers with a size of less than 15 nm. Several literature sources demonstrate the capability of dendrimer targeting ligands to cause the precise targeting and eradication of tumors. In addition to oligosaccharides, polysaccharides, oligopeptides, and polyunsaturated fatty acids, they also contain folate and cancer associated antigen
16	Magnetic Nanoparticle	By using electron microscopy, it was discovered that this particle only showed up in melanoma cells. When a 43 °C external alternating magnetic field was utilized to heat up the tumor, it was found that melanoma cells started to break down
17	Nanostructured lipid carriers	NLCs are a superior alternative to Solid-lipid nanoparticles (SLNs) for topical preparation due to several advantages associated with the use of biodegradable lipids, which guarantee low cytotoxicity and systemic toxicity. Due to the presence of solid lipid in it, small NLC particles may promote the intimate contact of the formulation with the stratum corneum, enhancing the drug's ability to pass through the skin. They may also enhance the controlled release of API from the carriers

## Conclusion

Skin carcinogenesis and its consequences show uncontrolled or aberrant expression of factors such as exposure to different genetic, epigenetic, drug, environmental, and pharmacological factors. The number of evidence indicates that NF-ĸB, STAT, and activator protein 1 promote genetic alterations while maintaining the stemness of cancer cells. Factors associated with skin cancer include exposure to UV rays, age, immunogenic responses, and viral agents. Various kinds of approaches are available till date such as chemotherapy, surgery, radiotherapy, chemical peel, immunotherapy, photothermal therapy, etc. Herein, we have outlined various examples of chemicals and pathways that appear to have promise for more efficient and secure therapies for several kinds of skin carcinoma. The dysregulation of several targeting sites orchestrates mediators and signaling pathways that converge toward skin cancer progression and metastasis, as evidenced by the complexity and heterogeneity of skin cancer cells. Therefore, it should be a top priority for research and clinical trials to identify novel therapeutics with multi-targeting potential from natural and non-natural resources that have fewer negative side effects. Nanotechnology is a growing approach that can overcome the stumbling block of existing approaches, such as undesired off-target effects, unspecific distribution, and suboptimal efficacy. Encouraging combination therapies using immunotherapy, phytocompounds, and chemotherapy can be a boon to overcoming the limitations of traditional therapy. Herein, we have discussed various kinds of chemotherapeutic agents, phytocompounds, and their combination for regulating the prognosis and metastasis cascade of skin cancer. To address the highlighted clinical problems in skin malignancies, more information regarding single-as well as combined therapy methods based on verified basic, translational, and clinical research are still desired.

## Data Availability

Not applicable.
